# FMO2 Promotes Angiogenesis via Regulation of N‐Acetylornithine

**DOI:** 10.1002/advs.202506618

**Published:** 2025-10-06

**Authors:** Jingyi Wang, Yinghui Xu, Xianpeng Wu, Mo Li, Changchen Xiao, Zaiyang Fu, Yongjian Chen, Qingju Li, Yating Ruan, Jing Zhao, Zhiwei Zhong, Jinghai Chen, Wei Zhu, Jinliang Nan, Cheng Ni, Xinyang Hu

**Affiliations:** ^1^ Department of Cardiology The Second Affiliated Hospital School of Medicine Zhejiang University Hangzhou 310009 China; ^2^ Breast Cancer Center Zhejiang Cancer Hospital Hangzhou 310022 China; ^3^ State Key Laboratory of Transvascular Implantation Devices Hangzhou 310009 China; ^4^ Heart Regeneration and Repair Key Laboratory of Zhejiang Province Hangzhou 310009 China; ^5^ Department of Cardiology The First Affiliated Hospital of Wenzhou Medical University Wenzhou 325000 China; ^6^ Transvascular Implantation Devices Research Institute Hang Zhou 310053 China; ^7^ Binjiang Institute of Zhejiang University Hangzhou 310053 China

**Keywords:** angiogenesis, endothelial cell metabolism, FMO2, N‐acetylornithine, NOTCH1

## Abstract

Endothelial cell (EC) metabolism is an emerging target for proangiogenic treatment of ischemic diseases; however, little is known about the metabolic alterations in ECs during ischemic diseases or vessel development stages. By conducting single‐cell transcriptome analysis, this work identifies flavin‐containing monooxygenase 2 (FMO2) as a pivotal regulator under multiple ischemic conditions. Targeted EC compensation of FMO2 in the genetic ablation model proved its proangiogenic function in various ischemic models and in the developing retina. Metabolomics combined with EC single‐cell sequencing revealed N‐acetylornithine as the top‐ranked altered metabolite regulated by FMO2, which inactivates NOTCH1 expression through the transcriptome regulation of activating transcription factor 3 (ATF3). N‐acetylornithine delivery displays a proangiogenic therapeutic effect in the ischemic models. The therapeutic effects of FMO2 and N‐acetylornithine can also be recapitulated in human ECs. These findings provide insights into the proangiogenic mechanisms underlying FMO2 and N‐acetylornithine, revealing potential targets to treat ischemic disease.

## Graphical abstract

This study identifies flavin‐containing monooxygenase 2 (FMO2) as a novel proangiogenic regulator in endothelial cells. Targeted FMO2 ablation impairs vessel sprouting, whereas its compensation potently enhances angiogenesis. Metabolomics and single‐cell sequencing reveal that FMO2 drives vascular growth via the N‐acetylornithine/ATF3/NOTCH1 axis. Therapeutic application of FMO2 or N‐acetylornithine improves outcomes in ischemic models, offering new therapeutic avenues for ischemic diseases.

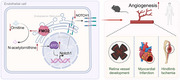

## 1 Introduction

Angiogenesis plays a crucial role in various physiological and pathological processes, including wound healing and tissue repair.^[^ ^1^, ^2^, ^3^, ^4^ ^]^ Endothelial cells (EC) can respond to ischemic stimuli to initiate angiogenesis.^[^ ^5^, ^6^, ^7^, ^8^ ^]^ Activated ECs undergoing a sprouting phenotype are called tip cells, which are central to new capillary bud formation to promote reparative angiogenesis.^[^ ^1^, ^9^, ^10^ ^]^ Hence, understanding the mechanisms underlying angiogenesis holds promise for developing interventions for ischemic diseases.

Metabolic reprogramming in ECs plays an essential role during angiogenesis by upholding increased energy demands and biosynthetic processes associated with vessel formation.^[^ ^11^, ^12^, ^13^, ^14^, ^15^ ^]^ According to classical opinions, metabolic pathways and metabolites are activated and cross‐talk to supply adequate energy and nutritious ingredients to fulfill the demand for EC bioactivities. In recent years, metabolites are considered to exert important biological effects in various pathological contexts through the direct modulation of downstream transcription factors or epigenetically reprogramming, such as tumor immune evasion and cardiomyocyte regeneration.^[^ ^16^, ^17^, ^18^ ^]^ However, such non‐canonical regulatory functions of metabolites during angiogenesis have rarely been reported. Therefore, it is of utmost significance to identify the key metabolites with dynamic alterations in ECs and hence uncover novel angiogenic mechanisms under ischemic pathological conditions.

Flavin‐containing monooxygenase 2 (FMO2), which uses NADP+ and FAD as coenzymes and cofactors to catalyze the oxidative metabolism of many xenobiotics,^[^ ^19^ ^]^ was suggested to exert a strong anti‐fibrotic effect after myocardial infarction (MI) in our previous study.^[^ ^20^ ^]^ What raises much interest is that FMO2 is expressed in ECs at a considerable level and conveys a significant reduction under ischemic conditions from a single‐cell dataset. However, the role of FMO2 in modulating angiogenesis has not been investigated. Thus, the role of FMO2 in orchestrating angiogenesis in ischemic diseases requires further investigation.

In this study, we identified a consistent reduction in FMO2 levels in ECs across multiple ischemic disease models. Genetic ablation of FMO2 specifically in ECs results in impaired angiogenesis under both MI and hindlimb ischemia (HLI) conditions. In contrast, targeted reinforcement of FMO2 in ECs exerts a significant proangiogenic function, as evidenced by enhanced vessel formation and blood flow perfusion. Mechanistically, by combining single‐cell RNA sequencing (scRNA‐seq) and non‐targeted metabolomics, we identified N‐acetylornithine as an important metabolite regulated by FMO2, which positively regulates activating transcription factor 3 (ATF3) transcription and inhibits NOTCH1 expression in an enzymatic activity‐dependent manner. More importantly, the direct delivery of N‐acetylornithine after MI and HLI exerted proangiogenic and organ recovery functions. In a clinical context, significantly reduced N‐acetylornithine concentrations can also be recaptured in patients with ST‐segment elevation myocardial infarction (STEMI) and peripheral artery disease (PAD) compared to non‐ischemic controls. Taken together, our data reveal the function of FMO2 and N‐acetylornithine in the coordination of the angiogenic process during ischemic conditions and vessel development across species, which endows potential clinical applications targeting angiogenesis in ischemic diseases.

## 2 Results

### 2.1 Endothelial FMO2 Promotes Vascular Development and Ischemic Angiogenesis

**Figure 1:**
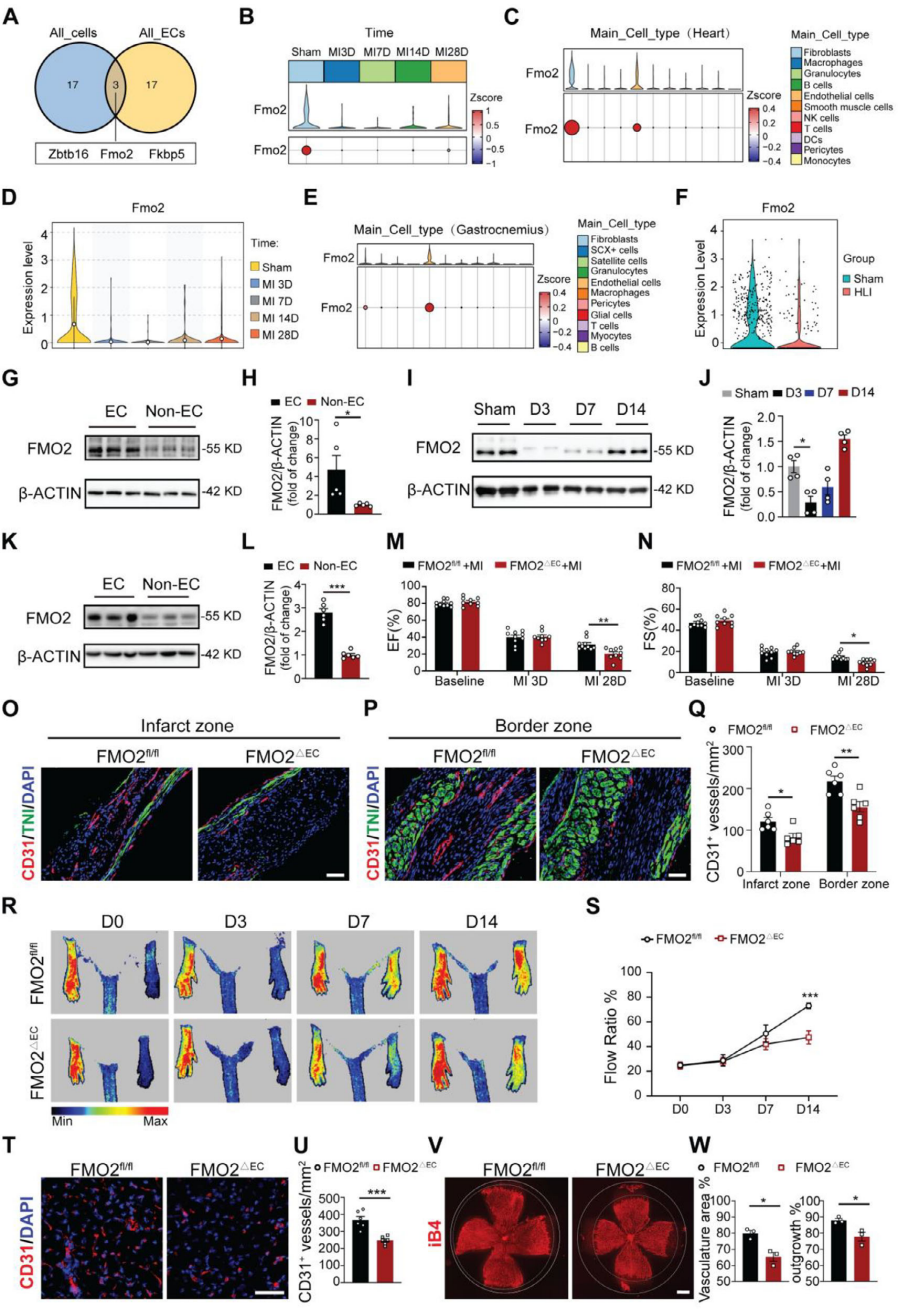
Endothelial‐specific FMO2 deficiency significantly impaired angiogenesis. A) The Venn diagram presents the cross‐correlation outcomes of the top 20 differentially expressed genes in whole cells and endothelial cells, which were analyzed using single‐cell RNA sequencing (scRNA‐seq) at diverse time points post‐MI. B) scRNA‐seq analysis showing the expression level of FMO2 in the whole heart of mice from sham and different time points post‐MI (3D, 7D, 14D and 28D). C) scRNA‐seq analysis displaying FMO2 expression in the heart among all cell types. D) scRNA‐seq analysis showed the altered expression of FMO2 in ECs from sham hearts and at different time points post‐MI (3D, 7D, 14D and 28D). E) scRNA‐seq analysis showed that FMO2 exhibited a high‐ranked in ECs from the gastrocnemius among all cell types. F) scRNA‐seq analysis showed that FMO2 in the ECs of mice gastrocnemius was downregulated post‐HLI compared to the sham group. G–H) Protein expression of FMO2 measured via western blot in gastrocnemius muscles from mice in the ECs and non‐ECs; results for two groups were normalized to β‐actin measurements and then to the mean of non‐ECs (*n =* 5 per group). I,J) Protein expression of FMO2 in ECs from the gastrocnemius muscle at 3, 7, and 14 days after HLI, and quantitative analysis are shown in (J) (*n =* 4 per group). K,L) Protein expression of FMO2 in ECs and non‐ECs from the retina, and quantitative analysis are shown in (L) (*n =* 6 per group). M,N) Echocardiographic assessments of left ventricular ejection fraction (EF) (M) and fractional shortening (FS) (N) were conducted at baseline, 3 days and 28 days after MI injury (FMO2^fl/fl^ +MI group, *n =* 10; FMO2^△EC^ +MI group, *n =* 9). O–Q) Immunostaining of the endothelial marker CD31 in sections from the infarct zone (O) and border zone (P) of FMO2^△EC^ and FMO2^fl/fl^ mice post‐MI. Cardiomyocytes were stained with troponin I (TNI), and the nuclei were co‐stained with DAPI. Scale bar, 50 µm. Vascular densities of the infarct and border zones were quantified in (Q) (*n =* 6 per group). R,S) HLI was surgically induced in 10‐week‐old FMO2^△EC^ mice and their control littermates. On Day0, Day3, Day7, and Day14 after HLI, blood flow was evaluated via laser Doppler imaging and quantified for each animal as the ratio of measurements of the injured (HLI) and uninjured (non‐HLI) limbs (FMO2^fl/fl^ group, *n =* 8, FMO2^△EC^ group, *n =* 10) (*** *P <* 0.001, FMO2^△EC^ versus FMO2^fl/fl^). T,U) Immunofluorescence staining for CD31 in the gastrocnemius muscles of FMO2^△EC^ and FMO2^fl/fl^ mice 14 days after HLI. Quantification of CD31‐positive vessels per mm^2^ (*n =* 6 per group). Scale bar, 50 µm. V–W) Whole‐mount P6 retinas from FMO2^fl/fl^ or FMO2^△EC^ pups were probed for isolectin B4 (iB4), and a statistical summary of the vascular area and length of the retinas is shown in (W) (*n =* 3 per group). Scale bar, 800 µm. Quantified data are presented as mean ± SEM. Unpaired two‐tailed Student's *t*‐test was conducted in H, L, U and W. One‐way ANOVA followed by Tukey's post hoc multiple comparisons test was conducted in J. Two‐way ANOVA followed by Tukey's post hoc multiple comparisons was conducted in M, N, Q, and S. ns *p >* 0.05, * *p <* 0.05, ** *p <* 0.01, and *** *p <* 0.001.

Given the essential role of angiogenesis in maladaptive remodeling following myocardial infarction (MI), we used this model to uncover the important molecular mechanisms responsible for post‐MI angiogenesis. Single‐cell RNA sequencing on heart tissues from mice subjected to MI at different timepoints was conducted and analyzed (Figure S1A, Supporting Information), and global cell distribution was visualized by uniform manifold approximation projection (UMAP) dimensionality reduction plots (Figure S1B, Supporting Information). To probe the hinge molecule involved in angiogenesis post‐ischemia, we identified the top 20 differentially expressed genes in both whole heart cells and specifically in cardiac ECs. Cross‐analysis of the differentially expressed gene sets revealed that only three genes (*Fmo2, Zbtb16*, and *Fkbp5*) were consistently and significantly downregulated in all cell types as well as in ECs (**Figure** **1**A, Table S1, Supporting Information). Further mRNA validation demonstrated that only *Fmo2* showed a significant reduction at 28 days post‐MI compared to the control group, whereas *Zbtb16*, *Fkbp5* did not change significantly (Figure S1C, Supporting Information). To clarify the expression pattern of *Fmo2* post‐MI, we analyzed the changes in *Fmo2* expression at different time points in whole cells. The results showed that *Fmo2* was significantly downregulated post‐MI compared with that in the sham group and retained low‐expression levels up to 28 days (Figure 1B). The expression of *Fmo2* was ranked highly in ECs among all cell types (Figure 1C), and in accordance with that in whole heart tissue, significantly reduced after MI and sustained for 28 days (Figure 1D, Figure S1D, Supporting Information).

To verify the alteration of *Fmo2* in other ischemic diseases, we used a publicly available database containing scRNA‐seq data of hindlimb ischemia (HLI) models^[^ ^21^ ^]^ (Figure S1E, Supporting Information). Analysis also indicated a significant reduction in *Fmo2* following HLI (Figure S1F, Supporting Information). Among the various cell types, ECs exhibited the highest levels of *Fmo2* (Figure 1E), and its expression was notably decreased post‐HLI (Figure 1F). The protein level confirmed that ECs from murine gastrocnemius muscle also exhibited enriched FMO*2* expression relative to non‐EC counterparts (Figure 1G,H), and FMO2 level in ECs was acutely downregulated in response to acute ischemia (Figure 1I,J). Furthermore, a similar FMO2 expression pattern was observed in ECs and non‐ECs of the murine retina on postnatal day 6 (P6) (Figure 1K,L), indicating the global effect of FMO2 on vessel growth and angiogenesis. Consistently, hypoxic treatment of ECs in vitro significantly reduced FMO2 protein expression versus controls (Figure S1G,H, Supporting Information).

To investigate the role of FMO2 in angiogenesis under ischemic conditions, we generated *Fmo2* global knockout (FMO2^−/−^) mice using CRISPR/Cas9. Fourteen days post‐HLI, FMO2^−/−^ mice displayed impaired blood perfusion (Figure S2A,B, Table S2, Supporting Information) and significant reductions in CD31^+^ vessels (Figure S2C,D, Supporting Information) compared to control animals, suggesting that FMO2 deficiency hinders angiogenesis in the hindlimb under ischemic conditions. To evaluate the role of FMO2 in maintaining vessel homeostasis, we observed early vascular development in the neonatal retina by staining with the endothelial marker isolectin B4 (iB4). The vascular network coverage and vessel length in FMO2^−/−^ mice were much lower than those in the control groups during development (Figure S2E‐G, Supporting Information), indicating that FMO2 deficiency postponed vascular development.

To further address whether FMO2‐mediated vascular regulation is EC‐specific, we generated EC‐specific *Fmo2* knockout mice (FMO2^△EC^) by crossbreeding FMO2^fl/fl^ mice with *Tie2*‐Cre transgenic mice (Figure S3A,B, Supporting Information). Following MI surgery, the recovery of cardiac performance was worse in FMO2^△EC^ mice than in FMO2^fl/fl^ mice, as evidenced by the reduced ejection fraction (EF) and fractional shortening (FS) evaluated at 28 days post‐MI (Figure 1M,N, Figure S3C, Table S3, Supporting Information). Moreover, CD31^+^ vessel density was much lower in the infarct and border zones of FMO2^△EC^ mice compared to controls, while remaining comparable in remote areas (Figure 1O–Q, Figure S3D,E, Supporting Information). Detailed analysis of vascular maturation revealed decreased pericyte coverage rate, proliferation rate and total pericyte proportion in FMO2^△EC^ infarcted hearts (Figure S3F–J, Supporting Information), along with markedly reduced α‐SMA^+^ vessel density in both infarct and border zones (Figure S3K,L, Supporting Information). These observations collectively demonstrated that EC‐specific FMO2 deletion impairs angiogenesis post‐MI. Consistent with these findings, FMO2^△EC^ mice in the HLI model exhibited sparser perfusion recovery (Figure 1R,S, Table S4, Supporting Information) and reduced CD31^+^ vessel density in the ischemic area below ligation (Figure 1T,U), accompanied by diminished pericyte coverage, suppressed proliferation, and decreased α‐SMA^+^ vessel density (Figure S3M–S, Supporting Information), further confirming the angiogenesis defect caused by FMO2 deficiency. To verify endothelial FMO2's role in ischemic myopathy, we performed histological analyses on murine ischemic gastrocnemius muscles. HE staining showed more extensive necrotic areas in FMO2^△EC^ mice, indicating impaired angiogenesis‐mediated muscle regeneration (Figure S3T,U, Supporting Information). In addition, the neonatal retinal vessel coverage area and length were also reduced in FMO2^△EC^ mice compared to those in control mice (Figure 1V,W). Taken together, these results demonstrate that the absence of FMO2 in ECs prominently disrupts angiogenesis in various ischemic diseases and during vascular development.

### 2.2 Endothelial‐specific FMO2 Compensation Reinforces Angiogenesis

**Figure 2:**
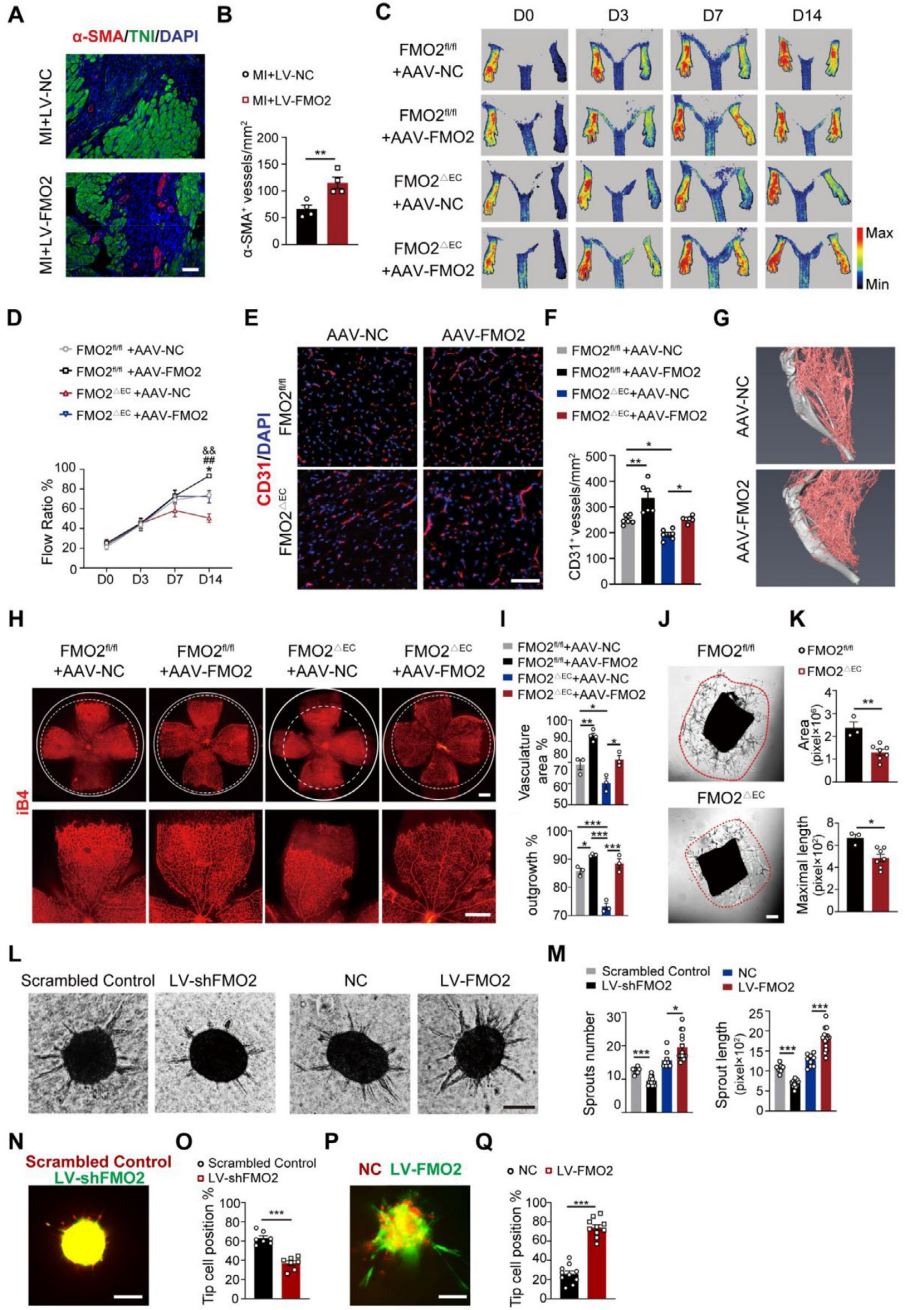
Impaired angiogenesis in FMO2^△EC^ mice can be rescued by the targeted administration of FMO2. A,B) Immunostaining of α‐SMA, TNI, and DAPI on the border zone of the hearts from mice intramyocardially injected with LV‐FMO2 or LV‐NC post‐MI. Scale bar, 50 µm. The α‐SMA‐positive vascular density of the border zone was quantified in (B) (*n =* 4 per group). C,D) Representative Doppler flow images after injection of AAV‐NC and AAV‐FMO2 in FMO2^fl/fl^ and FMO2^∆EC^ groups. Quantitative summary of the change in the rate of recovery of perfusion (Flow Ratio%) in each group is shown in (D) (FMO2^fl/fl^ +AAV‐NC group, *n =* 6; FMO2^fl/fl^ +AAV‐FMO2 group, *n =* 7; FMO2^△EC^+AAV‐NC group, *n =* 9; FMO2^△EC^ +AAV‐FMO2 group, *n =* 6); (* *P <* 0.05, FMO2^fl/fl^+AAV‐NC versus FMO2^fl/fl^ +AAV‐FMO2; ## *P <* 0.01, FMO2^fl/fl^+AAV‐NC versus FMO2^∆EC^+AAV‐NC; && *P <* 0.01, FMO2^∆EC^+AAV‐NC versus FMO2^∆EC^+AAV‐FMO2). E,F) CD31 staining on gastrocnemius sections from mice of FMO2^fl/fl^+AAV‐NC, FMO2^fl/fl^ +AAV‐FMO2, FMO2^∆EC^+AAV‐NC, and FMO2^∆EC^+AAV‐FMO2 groups post‐HLI. Vascular density was quantified as the number of capillaries per mm^2^ (*n =* 6 per group). Scale bar, 50 µm. G) Representative micro‐computed tomography (micro‐CT) images of the AAV‐NC and AAV‐FMO2 groups. H,I) Whole‐mount retinas from FMO2^fl/fl^ or FMO2^∆EC^ pups supplemented with AAV‐NC or AAV‐FMO2 were probed for iB4. Vascular area and length of retinas were quantified in (I). Scale bar, 800 µm. (FMO2^fl/fl^ +AAV‐NC group, *n =* 3; FMO2^fl/fl^ +AAV‐FMO2 group, *n =* 4; FMO2^△EC^+AAV‐NC group, *n =* 3; FMO2^△EC^ +AAV‐FMO2 group, *n =* 3). J,K) Representative images of sprouts from aortic rings. Quantification of maximal sprout length and vasculature area of aortic rings is shown in (K) (FMO2^fl/fl^, *n =* 3; FMO2^△EC^, *n =* 7). (L‐M) Representative images of spheroids after infection with LV‐shFMO2 or LV‐FMO2. The number of sprouts per spheroid and total sprout length of spheroids were quantified in (M) (Scrambled Control, *n =* 7; LV‐shFMO2, *n =* 13; NC, *n =* 9; LV‐FMO2, *n =* 15). N,O) Mosaic spheroids containing a 1:1 mixture of Scrambled Control^RED^ and FMO2‐silenced (LV‐shFMO2^GFP^) ECs showed a decreased number of LV‐shFMO2^GFP^ ECs at the tip position. The quantification of the fraction of tip cells with the indicated FMO2 genotypes is shown in (O), revealing the competitive disadvantage of FMO2^KD/GFP^ ECs in reaching the tip (*n =* 7 per group). P,Q) Morphological quantification of spheroid sprouting revealed that FMO2‐OE (FMO2^OE/GFP^) ECs generated tip cell sites at a higher frequency (*n =* 10 per group). Quantified data are presented as mean ± SEM. Unpaired two‐tailed Student's *t*‐test was conducted in B, K, M, O, and Q. Two‐way ANOVA followed by Tukey's post hoc multiple comparisons was conducted in D, F, and I. ns *p >* 0.05, * *p <* 0.05, ** *p <* 0.01, and *** *p <* 0.001.

To explore the therapeutic potential of FMO2 in ischemic conditions, FMO2‐overexpressing lentivirus (LV‐FMO2) or vector (LV‐NC) was intramyocardially injected into mice after MI surgery (Figure S4A,B, Supporting Information). Twenty‐eight days after MI, we observed substantially increased α‐SMA^+^ vessels localized around the border zone in the LV‐FMO2 group compared with the NC group (**Figure** **2**A,B), indicating that boosting FMO2 expression led to reinforcement of vascular density under ischemic conditions. To precisely determine the proangiogenic function of endothelial FMO2 augmentation, we injected both FMO2^△EC^ and control mice with a gastrocnemius muscle‐local injection of EC‐specific FMO2 overexpressing adeno‐associated virus with *Tie2* promoter (AAV‐FMO2) or control virus (AAV‐NC) four weeks before hindlimb ischemia surgery (Figure S4C, Supporting Information). We validated the endothelial‐specific transduction of AAV‐FMO2 by sorting ECs and non‐ECs from the injected muscle tissues. Immunoblotting confirmed higher FMO2 overexpression efficiency in sorted ECs than in non‐endothelial populations (Figure S4D–G, Supporting Information). Restoration of FMO2 in ECs in FMO2^△EC^ mice considerably enhanced hindlimb blood perfusion (Figure 2C,D, Table S5, Supporting Information) and remarkably augmented CD31^+^ vessels (Figure 2E,F) compared with FMO2^△EC^ mice injected with AAV‐NC. Micro‐CT reconstruction of the ischemic hindlimb also revealed a substantial improvement in hindlimb vascularity in AAV‐FMO2 treatment mice compared to AAV‐NC controls (Figure 2G). Moreover, retinas harvested at P5 exhibited a conspicuous regain of vessel coverage area and vessel length in the AAV‐FMO2 group relative to the AAV‐NC group (Figure 2H,I, Figure S4H,I, Supporting Information). These data demonstrate that FMO2 compensation in ECs is sufficient to facilitate developmental vessel formation and promote angiogenic repair processes under ischemic conditions.

To gain a deeper understanding of how FMO2 regulates angiogenesis in ECs, we conducted an aorta ring assay to evaluate the sprouting ability. Aorta isolated from FMO2^△EC^ mice exhibited substantial attenuation of endothelial cell extension, accompanied by a lower maximum length of sprouting compared to FMO2^fl/fl^ controls after 7 days in vitro culture (Figure 2J,K). In addition, spheroid sprouting and tube formation assays showed that FMO2 overexpression boosted the angiogenic capacity of ECs, whereas FMO2 silencing caused the opposite effect (Figure 2L,M, Figure S4J‐O, Supporting Information). These in vitro results comprehensively confirmed that FMO2 facilitates vessel sprouting.

Tip cells serve as central regulators during the process of sprouting, including guiding the direction of vessel growth through extracellular cellular matrix (ECM) sensing, proteolytic remodeling, and paracrine signaling.^[^ ^1^, ^9^ ^]^ To explore whether FMO2 facilitated sprouting ability was associated with tip‐cell transformation, we performed a mosaic spheroid assay on ECs transfected with lentivirus expressing shFMO2 and EGFP (FMO2^KD/GFP^) or the vector labeled with Pkh26 (NC^RED^) and co‐cultured these two types of lentiviral‐infected ECs at a ratio of 1:1. We observed that FMO2^KD/GFP^ cells were less extended at the tip than NC^RED^ cells after 8 h of culture (Figure 2N,O). In contrast, ECs with FMO2 overexpression lentivirus (FMO2^OE/GFP^) exhibited strengthened sprouting ability as GFP^+^ cells occupied the majority at the tip relative to NC^RED^ cells, illustrating that FMO2^OE/GFP^ significantly enhanced the potential of tip‐cell transformation (Figure 2P,Q). Taken together, these experiments indicate that FMO2 in ECs endows their ability to acquire the tip‐cell phenotype, which promotes vessel sprouting.

### 2.3 FMO2 Promotes the Sprouting Ability of Blood Vessels by Repressing NOTCH1

**Figure 3:**
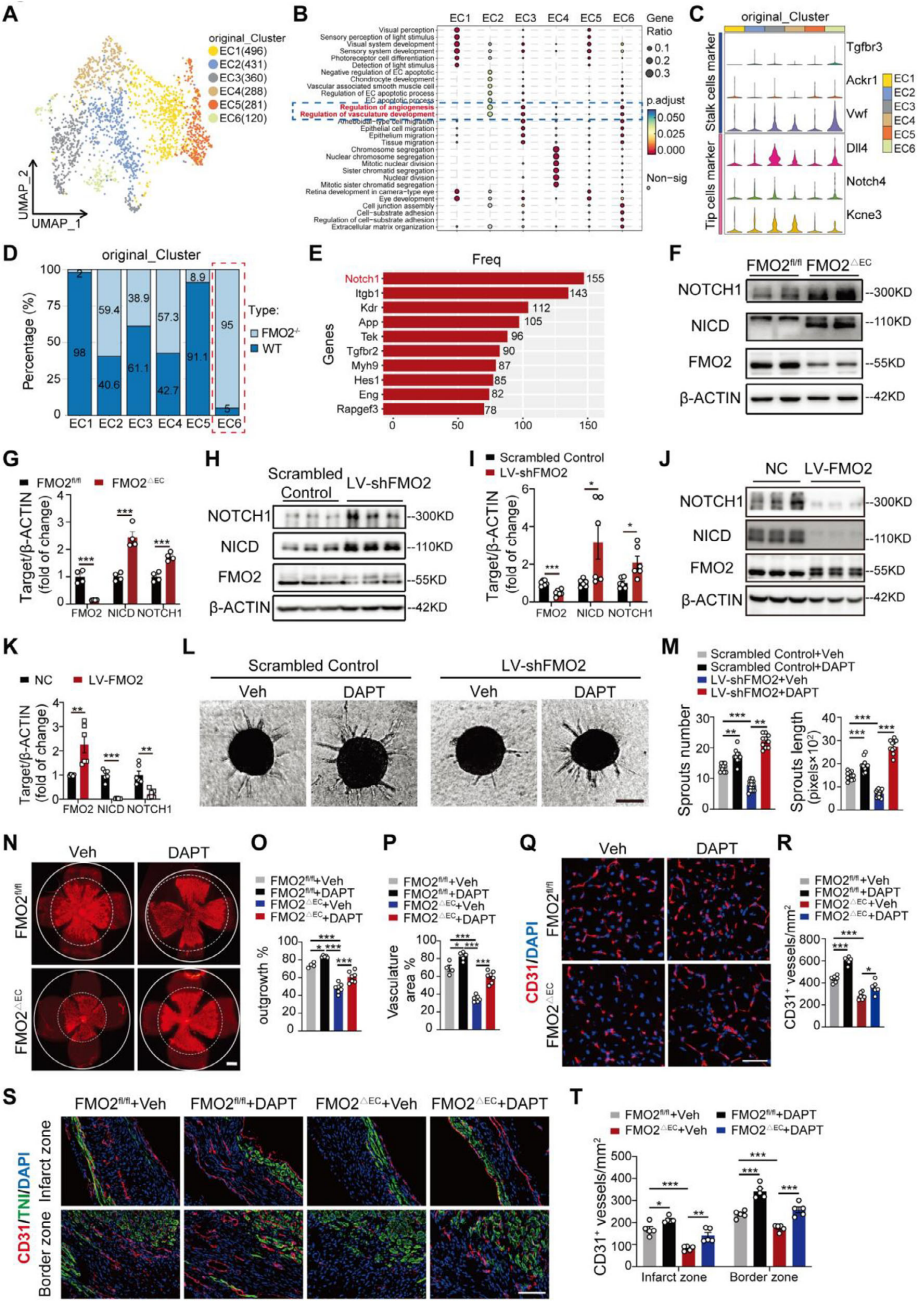
FMO2 promotes angiogenesis by inhibiting NOTCH1. A) UMAP of combined EC cells revealed six subclusters based on transcriptional similarity. B) GO enrichment analysis of the six clusters. C) Expression levels of different endothelial cell markers in the six clusters. D) The respective proportions of each EC cluster in the WT and FMO2^−/−^ groups. E) Gene score analysis of the EC6 cluster. F,G) Western blot analysis of FMO2, NICD and NOTCH1 expression in ECs from FMO2^fl/fl^ and FMO2^△EC^ mice after HLI, and quantitative analysis was plotted in (G) (*n =* 4 per group). H,I) Western blot analysis of FMO2, NICD and NOTCH1 levels in ECs infected with Scrambled Control or LV‐shFMO2, and quantitative analysis was plotted in (I) (*n =* 6 per group). J,K) Western blot analysis of FMO2, NICD and NOTCH1 levels in ECs infected with NC or LV‐FMO2, and quantitative analysis were plotted in (K) (*n =* 6 per group). L,M) Representative images and quantitative analysis of spheroids in the scramble control or LV‐shFMO2 after vehicle or DAPT treatment. Scale bars, 50 µm. (Scrambled control+Vehicle group, *n =* 10; Scrambled control+DAPT group, *n =* 10; LV‐shFMO2+Vehicle group, *n =* 14; LV‐shFMO2 +DAPT group, *n =* 10). N–P) Whole‐mount P6 retinas from FMO2^fl/fl^ or FMO2^△EC^ pups were probed for iB4 after administration of DAPT. Vascular area and length of retina were quantified in (O) and (P). Scale bar, 800 µm. (FMO2^fl/fl^+Vehicle group, *n =* 4; FMO2^fl/fl^+DAPT group, *n =* 5; FMO2^△EC^+Vehicle group, *n =* 7; FMO2^△EC^+DAPT group, *n =* 7). Q,R) CD31 staining of gastrocnemius sections from mice of the FMO2^fl/fl^+Vehicle, FMO2^fl/fl^+DAPT, FMO2^∆EC^+Vehicle, and FMO2^∆EC^+DAPT groups post‐HLI. Vascular density was quantified as the number of CD31‐positive capillaries per mm^2^ (*n =* 6 per group). Scale bar, 50 µm. S,T) CD31 staining in the infarct and border zones of MI hearts of FMO2^fl/fl^+Vehicle, FMO2^fl/fl^+DAPT, FMO2^∆EC^+Vehicle, and FMO2^∆EC^+DAPT groups. Cardiomyocytes were visualized by staining for cardiac troponin I (TNI), and nuclei were counterstained with DAPI. The vascular densities of the infarct and border zones were quantified in (T) (*n =* 5 per group). Scale bar, 100 µm. Quantified data are presented as mean ± SEM. Unpaired two‐tailed Student's *t*‐test was conducted in G, I, and K. Two‐way ANOVA followed by Tukey's post hoc multiple comparisons was conducted in M, O, P, R, and T. ns *p >* 0.05, * *p <* 0.05, ** *p <* 0.01, and *** *p <* 0.001.

To delineate how FMO2 regulates tip‐cell transformation of ECs, we sorted ECs from the retinas of FMO2^−/−^ and control neonatal mice, followed by scRNA‐seq. All ECs were subclustered into six subsets (EC1‐EC6), which were visualized using UMAP dimensionality reduction plots (**Figures** **3**A, S5A,B, Supporting Information). Gene Ontology (GO) analysis on six subclusters uncovered that EC3 and EC6 were highly enriched with angiogenesis‐related signaling (Figure 3B), hinting that these two subclusters may have an intimate relationship with angiogenic ability. Through marker annotation, we identified that EC6 highly expressed stalk cell‐enriched genes such as *Tgfbr3*, *Vwf and Ackr1*, but low‐expressed tip cell‐enriched genes such as *NOTCH4, Kcne3, and Dll4*. (Figure 3C), whereas the latter genes were highly enriched in EC3. Pseudo‐time analysis of all clusters indicated that EC6 can be transformed into EC3 through two tracks (Figure S5C, Supporting Information). These results identified that the EC6 subpopulation exhibits a stalk cell‐like phenotype and characteristics. Strikingly, we discovered that the EC6 subset was nearly unique to FMO2^−/−^ ECs (Figure 3D); therefore, we speculated that EC6 may play a fundamental role in hindering angiogenesis. To analyze the detailed molecular pathway responsible for stalk cell phenotypic transformation, we tested the gene frequency in the significantly enriched pathways within EC6. The results showed that *NOTCH1* had the highest frequency in EC6 among all enriched genes (Figure 3E). More importantly, *NOTCH1* expression was the highest (Figure S5D, Supporting Information) in EC6 compared to other subsets. These results strongly indicate that *NOTCH1* in EC6 plays a vital role in sustaining stalk cell formation and inhibits angiogenesis.

To validate FMO2‐mediated regulation of NOTCH1 in ECs, we conducted qPCR and confirmed elevated *NOTCH1* mRNA expression in ECs isolated from the retina of FMO2^△EC^ compared to FMO2^fl/fl^ at P6 (Figure S5E, Supporting Information). Moreover, FMO2 knockdown in ECs increased *NOTCH1* mRNA in vitro (Figure S5F, Supporting Information), while FMO2 overexpression decreased *NOTCH1* transcription (Figure S5G, Supporting Information). At protein levels, both NOTCH1 and NOTCH1 intracellular domain (NICD) were upregulated in ECs from FMO2^△EC^ gastrocnemius muscle upon HLI (Figure 3F,G), and this effect was reversed by AAV‐FMO2 delivery (Figure S5H,I, Supporting Information). In addition, we visualized active Notch1 (NICD) using fluorescence staining on the frozen sections from ischemic gastrocnemius muscles and infarcted hearts, showing a higher proportion of NICD^+^ ECs in FMO2^△EC^ mice post‐HLI or MI versus controls (Figure S5J–M, Supporting Information). *In vit*ro, FMO2 knockdown similarly elevated NOTCH1 and NICD protein levels (Figure 3H,I), whereas FMO2 overexpression suppressed them (Figure 3J,K).

To further confirm whether NOTCH1 is highly involved in FMO2‐mediated angiogenesis, we used DAPT, a classic γ‐secretase inhibitor, to suppress NOTCH1 signaling.^[^ ^22^ ^]^ The application of DAPT restored the impaired sprouting ability of ECs following FMO2 knockdown (Figure 3L,M). Moreover, in FMO2^△EC^ mice, DAPT administration not only restored the diminished vascular coverage but also significantly elongated the vessel structures in the retina (Figure 3N–P). In the HLI model, DAPT‐mediated NOTCH1 inhibition effectively ameliorated blood perfusion deficits (Figure S6A,B, Table S6, Supporting Information) and increased CD31^+^ vessel density (Figure 3Q,R) in FMO2^△EC^ mice compared to controls. DAPT treatment further reduced the elevated NOTCH1 and NICD levels in FMO2‐deficient ECs (Figure S6C,D, Supporting Information). At 28 days post‐MI, DAPT treatment substantially rescued the decline in EF and FS observed in FMO2^△EC^ mice (Figure S6E,F, Table S7, Supporting Information), concomitantly with increased CD31^+^ vessel density in the border and infarct zones (Figure 3S,T). In addition, western blot analysis demonstrated that DAPT treatment normalized the elevated levels of NOTCH1 and NICD in FMO2‐deficient mice (Figure S6G,H, Supporting Information), highlighting the substantial effect of NOTCH1 inhibition in promoting angiogenesis when FMO2 was ablated. Collectively, these findings underscore the importance of NOTCH1 pathway in FMO2 regulation during angiogenesis.

### 2.4 Endothelial FMO2 Suppresses NOTCH1 through N‐acetylornithine

Previous studies have established that vascular endothelial growth factor A (VEGFA), vascular endothelial growth factor C (VEGFC), delta like canonical Notch ligand 4 (DLL4), and C‐X‐C motif chemokine receptor 4 (CXCR4) function as Notch‐dependent signaling molecules, which interact with NOTCH1 through hierarchical regulatory networks to coordinate angiogenesis.^[^ ^23^, ^24^ ^]^ However, our data revealed no significant alterations in their protein expression levels following FMO2 overexpression or knockdown in vitro (Figure S7A–P, Supporting Information) as well as in ECs isolated from FMO2^fl/fl^ or FMO2^△EC^ mice (Figure S7Q–V, Supporting Information), suggesting that FMO2 likely regulates NOTCH1 through alternative mechanisms distinct from direct modulation of these canonical factors.

**Figure 4:**
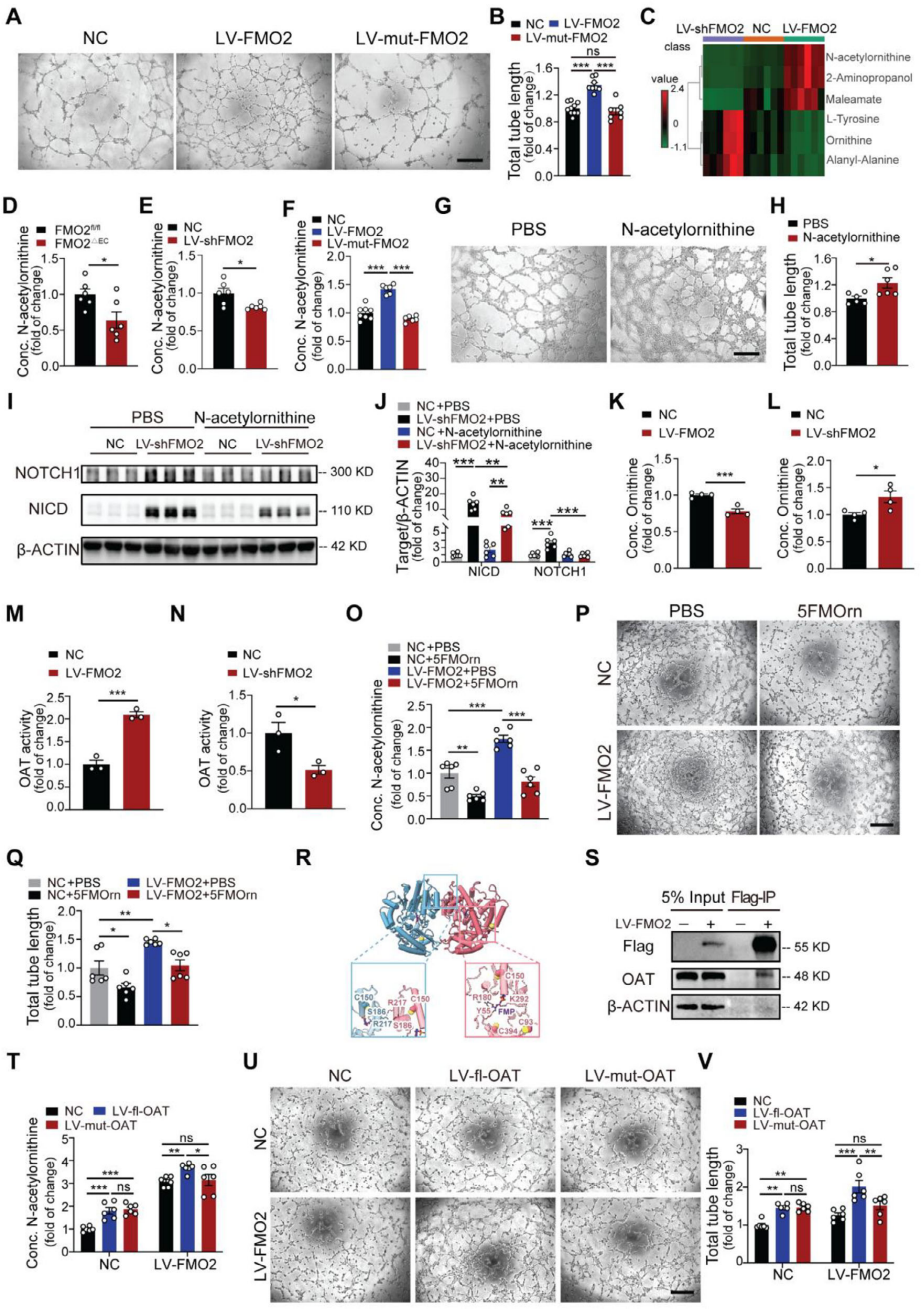
N‐acetylornithine generated by endothelial FMO2 represses NOTCH1. A,B) Representative images of tube formation and quantitative statistics of the total tube length of ECs infected with NC (*n =* 10), LV‐FMO2 (*n =* 8), and LV‐mut‐FMO2 (*n =* 7). Scale bar, 200 µm. C) Heatmap of metabolomics including all significant altered metabolites. (left: LV‐shFMO2 group, middle: NC group, and right: LV‐FMO2 group) D) Quantitative analysis of N‐acetylornithine levels by UHPLC–MS/MS in the FMO2^fl/fl^ and FMO2^△EC^ gastrocnemius after HLI (*n =* 6 per group). E) Quantitative analysis of N‐acetylornithine levels by UHPLC–MS/MS in the scrambled control and LV‐shFMO2 groups (*n =* 6 per group). F) Quantitative analysis of N‐acetylornithine levels by UHPLC–MS/MS in the NC (*n =* 8), LV‐FMO2 (*n =* 6) and LV‐mut‐FMO2 (*n =* 6) groups. G,H) Representative images of tube formation and quantitative statistics of the total tube length of ECs formed after PBS or N‐acetylornithine treatment (*n =* 6 per group). Scale bar, 200 µm. I,J) Western blot analysis of NICD and NOTCH1 levels in ECs infected with Scrambled Control or LV‐shFMO2, followed by PBS or N‐acetylornithine treatment (*n =* 6 per group). K) Quantitative analysis of Ornithine levels by ELISA in the NC and LV‐FMO2 groups (*n =* 4 per group). L) Quantitative analysis of Ornithine levels by ELISA in the NC and LV‐shFMO2 groups (*n =* 4 per group). M) Enzymatic activity of OAT in the LV‐FMO2 and NC groups (*n =* 3 per group). N) Enzymatic activity of OAT in the LV‐shFMO2 and NC groups (*n =* 3 per group). O) Quantitative analysis of N‐acetylornithine levels by UHPLC–MS/MS in the NC+PBS, NC+5FMOrn, LV‐FMO2+PBS, and LV‐FMO2+5FMOrn groups (*n =* 6 per group). P,Q) Representative images of tube formation and quantitative statistics of the total tube length of ECs in the NC+PBS, NC+5FMOrn, LV‐FMO2+PBS, and LV‐FMO2+5FMOrn groups (*n =* 6 per group). Scale bar, 500 µm. R) Overall structure of the OAT dimer with two OAT molecules (PDB ID: 2OAT). The two molecules are shown as pink and blue cartoons, respectively. The lower left panel shows the interaction between S186 and R217 in the dimer interface, whereas the lower right panel is a schematic drawing of the interaction of the FMP adduct (purple) with the active site residues (Y55, R180 and K292). In both panels, hydrogen bonds are represented by black dashed lines, and the cysteine sites (C93, C150 and C394) are marked as yellow spheres. S) Immunoblot analysis of OAT in Flag‐tagged immunoprecipitates from the lysates of ECs infected with either scrambled control or LV‐FMO2. T) Quantitative analysis of N‐acetylornithine levels by UHPLC–MS/MS in ECs transfected with NC or LV‐FMO2 and further infected with NC, LV‐fl‐OAT, or LV‐mut‐OAT (*n =* 6 per group). U,V) Representative images of tube formation and quantitative statistics of the total tube length of ECs transfected with NC or LV‐FMO2 and further infected with NC, LV‐fl‐OAT, or LV‐mut‐OAT (*n =* 6 per group). Scale bar, 500 µm. Quantified data are presented as mean ± SEM. Unpaired two‐tailed Student's *t*‐test was conducted in D, E, H, K, L, M, and N. One‐way ANOVA followed by Tukey's post hoc multiple comparisons test was conducted in B and F. Two‐way ANOVA followed by Tukey's post hoc multiple comparisons was conducted in J, O, Q, T, and V. ns *p >* 0.05, * *p <* 0.05, ** *p <* 0.01, and *** *p <* 0.001.

Given that FMO2 is an NADP^+^‐ and FAD‐dependent enzyme, to validate whether the effect of FMO2 on angiogenesis is enzymatically dependent, we generated lentivirus harboring FMO2 segment with enzymatic activity deficiency mutation (LV‐mut‐FMO2),^[^ ^20^ ^]^ structural mimicking identified that the deletion of enzymatic activity segments did not affect the correct fold of FMO2 protein (Figure S8A,B, Supporting Information). We discovered that the loss of enzymatic activity abolished the proangiogenic functions of FMO2, which was reflected by impaired tube formation and tip‐cell sprouting (**Figure** **4**A,B, Figure S8C,D, Supporting Information), indicating that FMO2 regulates angiogenesis in an enzyme activity‐dependent manner. Based on these findings, we hypothesized that FMO2 regulates NOTCH1 through the action of certain bioactive metabolites. To this end, we conducted untargeted metabolomics analysis of ECs subjected to either LV‐FMO2 or LV‐shFMO2 lentivirus treatment. Through pairwise comparisons, three metabolites (N‐acetylornithine, 2‐Aminopropanol, and Maleamate) exhibited significant elevation in the LV‐FMO2 group and reduced levels in the LV‐shFMO2 group, whereas three metabolites (L‐Tyrosine, Ornithine and Alanyl‐Alanine) exhibited significant reduction in the LV‐FMO2 group with increased levels in the LV‐shFMO2 group (Figure 4C, Figure S8E–G, Table S8, Supporting Information). Among all the differential metabolites, we discovered that ornithine and N‐acetylornithine were simultaneously enriched in the ornithine metabolic pathway. In addition, the change in the content of upregulated N‐acetylornithine with downregulated ornithine also conformed to the catalyzing procedure in the ornithine pathway. Thus, we verified the content of N‐acetylornithine both in vivo and in vitro, and the content of N‐acetylornithine in the gastrocnemius muscle was found to be significantly decreased in FMO2^△EC^ mice upon HLI compared with controls (Figure 4D). Consistently, the concentration of N‐acetylornithine in cultured ECs was significantly upregulated along with FMO2 overexpression and decreased when FMO2 was knocked down or subjected to truncated enzymatic inactivity (Figure 4E,F). To verify whether N‐acetylornithine and other metabolites facilitate angiogenesis, we added N‐acetylornithine, 2‐Aminopropanol and Maleamate, which were significantly upregulated after LV‐FMO2 treatment, to the culture medium of ECs. We observed that only N‐acetylornithine treatment dramatically increased the tube formation capacity of the ECs (Figure 4G,H, Figure S8H,I, Supporting Information). Furthermore, the addition of N‐acetylornithine repressed the upregulation of NOTCH1 and NICD expression induced by FMO2 interference (Figure 4I,J, Figure S8J,K, Supporting Information).

In contrast, the content of ornithine, which acts as an upstream metabolite of N‐acetylornithine, was significantly increased after FMO2 knockdown but was critically inhibited by FMO2 overexpression, further validating the results from metabolomics (Figure 4K,L). However, the glutamate content showed no alteration under FMO2 modulation, and the expression of key enzymes catalyzing glutamate to N‐acetylornithine transition also remained unchanged, indicating that the metabolic pathway from glutamate to N‐acetylornithine was not affected (Figure S8L‐N, Supporting Information). Taken together, these findings indicate that FMO2 regulates the metabolic pathway from ornithine to N‐acetylornithine. In the context that ornithine aminotransferase (OAT) acts as an important enzyme that plays an important role in converting ornithine to L‐glutamate‐5‐semialdehyde, which is the primary precursor for the synthesis of N‐acetylornithine, we further tested the enzymatic activity and expression level of OAT. The results indicated that FMO2 compensation effectively promoted OAT enzymatic activity, whereas the expression level remained unaffected. In contrast, FMO2 knockdown inhibited the activity of OAT (Figure 4M,N, Figure S8N, Supporting Information), indicating that FMO2 regulates OAT‐mediated conversion of ornithine to N‐acetylornithine. To further confirm whether the catalytic activity of OAT is necessary during FMO2‐mediated proangiogenic function, we next added 5‐Fluoromethylornithine dihydrochloride (5FMOrn), a well‐established OAT inhibitor, to cultured ECs under FMO2 compensation. The increased N‐acetylornithine content due to FMO2 overexpression was significantly inhibited in the context of 5FMOrn treatment, whereas the decreased ornithine content was dramatically upregulated (Figure 4O, Figure S8O, Supporting Information). In addition, 5FMOrn processing in cultured ECs significantly blocked the proangiogenic function of FMO2 overexpression, as revealed by the inhibition of tube formation (Figure 4P‐Q). The protein expression of NOTCH1 and NICD was also significantly elevated in cultured ECs by 5FMOrn administration, even after FMO2 compensation (Figure S8P,Q, Supporting Information).

Knowing that OAT plays a pivotal role in FMO2‐mediated metabolism, we next investigated how FMO2 regulates OAT enzymatic activity. Our investigations into the structure‐function relationship of OAT protein have revealed intriguing insights into the critical role of cysteine residues in maintaining enzymatic activity. This structure shows that wild‐type OAT functions as a dimer, with a stable hydrogen bond interaction between R217 and S186 at the dimer interface. The catalytic center, composed of Y55, R180, and K292, is located distal to the interface. The cysteine residues of interest are strategically positioned within these crucial regions: C150 is proximal to the dimer interface, whereas all three cysteine residues (C93, C150, and C394) are near the catalytic center. These structural observations led us to hypothesize that mutations in these cysteine residues may impair OAT activity by disrupting dimer formation or altering the catalytic center conformation (Figure 4R). In addition, cysteine residues commonly act as central regulators in balancing oxidation‐reduction reaction conditions in mammals. Our previous studies also indicated that FMO2 can lead to protein oxidative modifications, including the oxidation of thiols (oxidation of the thiol group (‐SH) in cysteine residues to disulfide bonds (‐S‐S‐).^[^ ^25^ ^]^ Based on the above theoretical basis, we speculate that FMO2 may affect the oxidation of cysteine residues in OAT, which promotes its catalytic activity and eventually results in an increased N‐acetylornithine content. Furthermore, co‐immunoprecipitation assays confirmed the potential interaction between FMO2 and OAT (Figure 4S). Hence, we generated lentiviruses harboring OAT with C93, C150, and C394 mutations (LV‐mut‐OAT) and transfected them into cultured ECs. We discovered that LV‐mut‐OAT diminished the augmented content of N‐acetylornithine under FMO2 overexpression conditions compared to LV‐full length OAT (LV‐fl‐OAT) (Figure 4T). More importantly, LV‐mut‐OAT transfection in ECs also inhibited the prolonged tube formation compared with the LV‐full length OAT processing group (Figure 4U,V), and NICD expression was also reversed to normal levels in ECs transfected with LV‐mut‐OAT, under FMO2 compensation (Figure S8R–T, Supporting Information). These results indicate that FMO2 potentially oxidizes the C93, C150, and C394 residues in OAT to enhance its enzymatic activity, thus promoting the conversion of ornithine to N‐acetylornithine.

In conclusion, we confirmed that FMO2 regulates the synthesis of N‐acetylornithine by modulating the ornithine metabolism pathway in an enzymatic activity‐dependent manner, which suppresses NOTCH1 expression and subsequently facilitates angiogenesis.

### 2.5 N‐acetylornithine Exerts a Potent Therapeutic Effect in Ischemic Diseases

**Figure 5:**
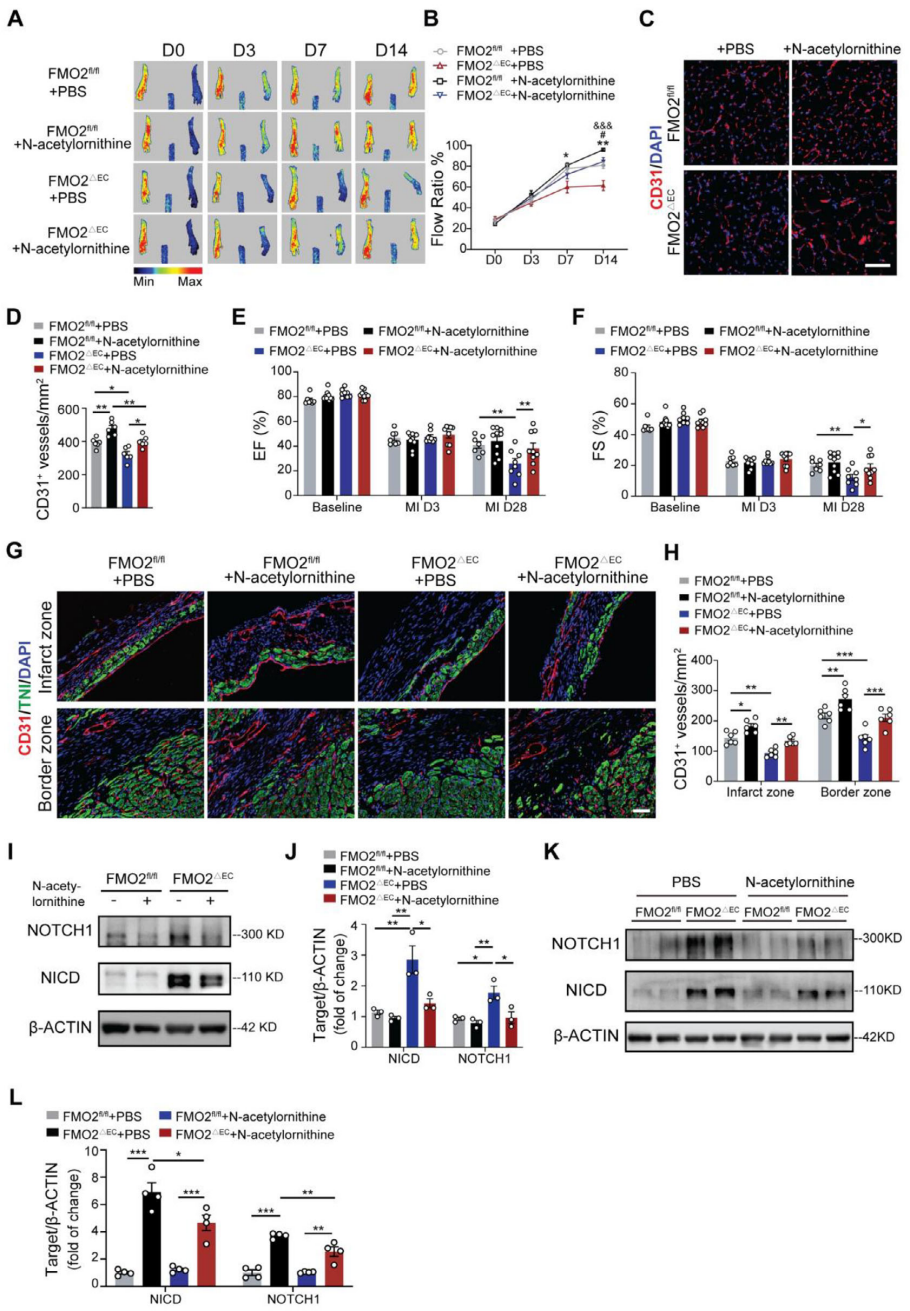
N‐acetylornithine exerts potent therapeutic effects in ischemic diseases. A,B) Representative images showing Doppler flow images after injection of N‐acetylornithine and PBS in the FMO2^fl/fl^ and FMO2^∆EC^ groups. Quantitative analysis of the change in the rate of recovery of perfusion (Flow Ratio%) in each group is shown in (B) (FMO2^fl/fl^+PBS, *n =* 7; FMO2^∆EC^+PBS, *n =* 9; FMO2^fl/fl^+N‐acetylornithine, *n =* 9; FMO2^∆EC^+N‐acetylornithine, *n =* 9). (*P < 0.05, **P < 0.01, FMO2^∆EC^+PBS versus FMO2^fl/fl^+PBS; #*P <* 0.05, FMO2^fl/fl^+N‐acetylornithine versus FMO2^fl/fl^+PBS; &&&*P <* 0.001, FMO2^∆EC^+N‐acetylornithine versus FMO2^∆EC^+PBS). C,D) CD31 staining on gastrocnemius sections from mice of FMO2^fl/fl^+PBS, FMO2^fl/fl^ +N‐acetylornithine, FMO2^∆EC^+PBS, and FMO2^∆EC^+N‐acetylornithine groups post‐HLI. Vascular density was quantified as the number of capillaries per mm^2^ (*n =* 6 per group). Scale bar, 50 µm. E,F) Echocardiographic assessments of left ventricular ejection fraction (EF) and fractional shortening (FS) were conducted at baseline, 3 days and 28 days after MI and injection of N‐acetylornithine and PBS in the FMO2^fl/fl^ and FMO2^∆EC^ groups, respectively (FMO2^fl/fl^+PBS group, *n =* 7; FMO2^fl/fl^+N‐acetylornithine group, *n =* 10; FMO2^∆EC^+PBS group, *n =* 8; FMO2^∆EC^+N‐acetylornithine group, *n =* 9). G,H) Immunofluorescent staining for CD31 in the infarct and border zones of MI hearts in each group. Cardiomyocytes were visualized by staining with troponin I (TNI), and nuclei were counterstained with DAPI. Scale bar, 50 µm. The vascular densities of the infarct and border zones were quantified in (H) (*n =* 6 per group). I,J) Immunoblots showing NICD and NOTCH1 protein expression in gastrocnemius ECs following PBS or N‐acetylornithine treatment of FMO2^fl/fl^ and FMO2^∆EC^ mice after HLI (*n =* 3 per group). K,L) Immunoblots showing NICD and NOTCH1 protein expression in cardiac ECs following PBS or N‐acetylornithine treatment of FMO2^fl/fl^ and FMO2^∆EC^ mice after MI (*n =* 4 per group). Quantified data are presented as mean ± SEM. Two‐way ANOVA followed by Tukey's post hoc multiple comparisons was conducted in B, D, E, F, H, J, and L. ns *p >* 0.05, * *p <* 0.05, ** *p <* 0.01, and *** *p <* 0.001.

To explore the translational potential of N‐acetylornithine in treating ischemic diseases, we delivered N‐acetylornithine to mice after HLI surgery via intramuscular injection. First, we constructed FITC‐N‐acetylornithine and processed it with cultured ECs for 24 h. Flow cytometry confirmed a significantly increased immunofluorescence intensity in ECs compared to in the vehicle group, indicating that N‐acetylornithine penetrated ECs (Figure S9A, Supporting Information). N‐acetylornithine supplement resulted in an accelerated recovery rate post‐surgery, as evidenced by the increased flow ratio detected by Doppler (**Figure** **5**A,B, Table S9, Supporting Information). N‐acetylornithine administration in FMO2^△EC^ mice efficiently diminished FMO2 deficiency‐induced microvessel impairments, as reflected by CD31 staining (Figure 5C,D), suggesting that N‐acetylornithine replenishment can counteract the detrimental effects of FMO2 deficiency, promoting angiogenesis even in compromised conditions.

Beyond the impact of N‐acetylornithine on peripheral vasculature, we also discovered that N‐acetylornithine administration in FMO2^△EC^ mice improved cardiac function, as revealed by improved EF and FS of the hearts subjected to MI (Figure 5E,F, Figure S9B, Table S10, Supporting Information). In addition, N‐acetylornithine treatment increased the micro vessel density around the infarct zone and border zone (Figure 5G,H) in FMO2^△EC^ mice 28 days after MI surgery, highlighting the critical role of N‐acetylornithine in enhancing myocardial vascularization and cardiac function recovery after infarction. N‐acetylornithine administration in FMO2^△EC^ mice also resulted in lower NOTCH1 and NICD levels in gastrocnemius ECs after HLI (Figure 5I,J, Figure S9C,D, Supporting Information) and cardiac ECs subjected to MI (Figure 5K,L). Furthermore, exogenous injection of N‐acetylornithine was safety in both FMO2^△EC^ and FMO2^fl/fl^ mice without affecting liver and kidney function, including alanine aminotransferase (ALT), aspartate aminotransferase (AST), globulin, and creatinine (Figure S9E–N, Supporting Information). Moreover, N‐acetylornithine administration in an ischemia/reperfusion model confirmed the cardioprotective effect, as reflected by improved heart function compared with the control group, validating the application of N‐acetylornithine in real clinical circumstances (Figure S9O,P, Supporting Information).

N‐acetylornithine administration demonstrated significant therapeutic effects in promoting angiogenesis and enhancing the recovery of organ function in cardiac and hindlimb ischemia models. These findings suggest that N‐acetylornithine holds considerable promise for clinical application as a novel proangiogenic therapeutic agent for ischemic disease.

### 2.6 N‐acetylornithine Restrains NOTCH1 via Activating ATF3

**Figure 6:**
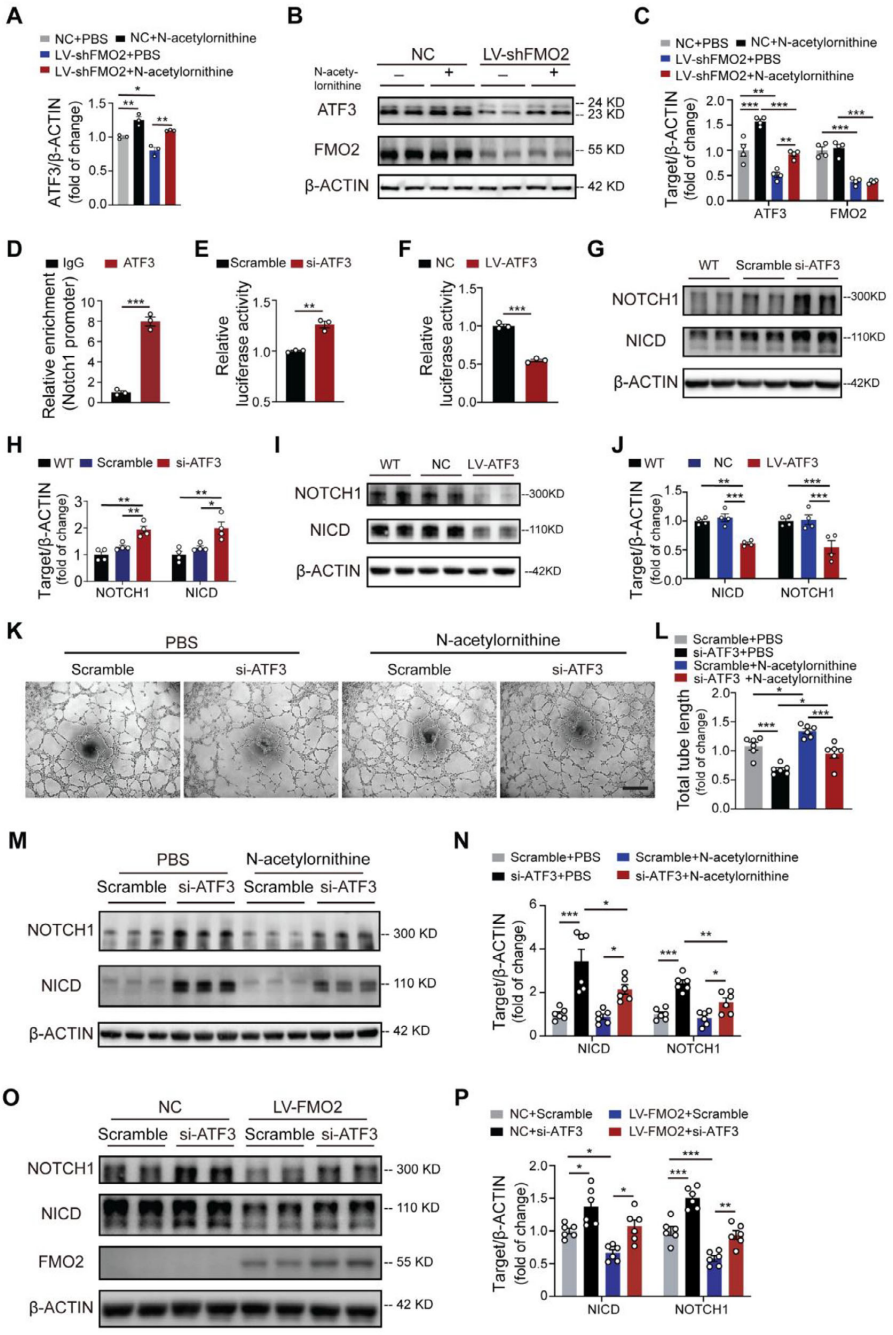
**N**‐acetylornithine modulates NOTCH1 by activating ATF3. A) mRNA expression of ATF3 in ECs following PBS or N‐acetylornithine treatment after infection with LV‐shFMO2 or NC (*n =* 3 per group). B,C) Immunoblots showing FMO2 and ATF3 protein expression in ECs following PBS or N‐acetylornithine treatment after infection with NC or LV‐shFMO2 (*n =* 4 per group). D) Chromatin immunoprecipitation (ChIP) assay and real‐time quantitative PCR showing the binding efficiency of ATF3 to the NOTCH1‐promoter in ECs (*n =* 3 per group). E) Comparison of the relative luciferase intensity of the NOTCH1‐promoter in ECs from the control and ATF3 knockdown groups (*n =* 3 per group). F) Comparison of the relative luciferase intensity of the NOTCH1‐promoter in ECs from the control and ATF3 overexpression groups (*n =* 3 per group). G,H) Immunoblots showing NICD and NOTCH1 protein expression in ECs after ATF3 silencing (*n =* 4 per group). I,J) Immunoblots showing NICD and NOTCH1 protein expression in ECs after ATF3‐overexpressing (*n =* 4 per group). K,L) Representative images of tube formation and quantitative statistics of the total tube length of ECs following PBS or N‐acetylornithine treatment after ATF3 silencing (*n =* 6 per group). Scale bar, 200 µm. M,N) Immunoblots showing NICD and NOTCH1 protein expression in ECs following PBS or N‐acetylornithine treatment after ATF3 silencing (*n =* 6 per group). O,P) Immunoblots showing FMO2, NICD and NOTCH1 protein expression in ECs following NC or LV‐FMO2 treatment after ATF3 silencing (*n =* 6 per group). Quantified data are presented as mean ± SEM. Unpaired two‐tailed Student's *t*‐test was conducted in D, E, and F. Two‐way ANOVA followed by Tukey's post hoc multiple comparisons was conducted in A, C, H, J, L, N, and P. ns *p >* 0.05, * *p <* 0.05, ** *p <* 0.01, and *** *p <* 0.001.

To elucidate the detailed mechanism by which N‐acetylornithine influences *NOTCH1* expression, we conducted a comprehensive screening of the pathways associated with the regulation of *NOTCH1* transcription. First, conserved transcription factor (TF) binding sites on the *NOTCH1* gene promoter integrated with the ENCODE ChIP‐seq peak database were used to screen potential TFs that could modulate *NOTCH1* transcription, and 21 candidates were screened (Figure S10A, Supporting Information). To identify the TF regulated by FMO2 and N‐acetylornithine, we evaluated the expression levels of all candidates and found that only ATF3 in ECs significantly declined at both mRNA and protein levels upon FMO2 knockdown, but could be restored by the addition of N‐acetylornithine (**Figure** **6**A–C), whereas the other 20 candidates did not show downregulation after FMO2 knockdown with restoration after N‐acetylornithine replenishment (Figure S10B, Supporting Information). Therefore, we selected ATF3 as the key TF that regulates NOTCH1 and can be modulated by N‐acetylornithine treatment. The binding sites of ATF3 on *NOTCH1* were predicted using the JASPAR database (Figure S10C, Supporting Information).

To confirm ATF3's direct transcriptional regulation of *NOTCH1*, we used chromatin immunoprecipitation (ChIP)‐qPCR, which revealed the specific binding of ATF3 to the *NOTCH1* promoter in ECs (Figure 6D). Furthermore, dual‐luciferase reporter assays showed that ATF3 knockdown enhanced *NOTCH1* promoter‐driven luciferase activity (Figure 6E, Figure S10D,E, Supporting Information), whereas ATF3 overexpression by lentivirus (LV‐ATF3) suppressed it (Figure 6F, Figure S10F,G, Supporting Information). These results suggest that ATF3 may inhibit the transcriptional activity of *NOTCH1* by directly binding to its promoter region, negatively regulating the expression of NOTCH1. Moreover, ATF3 silencing in ECs resulted in upregulated *NOTCH1* transcriptional levels and augmented NOTCH1 and NICD expression, compared with the control groups (Figure 6G,H, Figure S10H, Supporting Information), whereas ATF3 overexpression downregulated NOTCH1 at transcriptional and protein levels, with a concomitant decrease in NICD expression (Figure 6I,J, Figure S10I, Supporting Information), further confirming the transcriptional regulatory effect of ATF3 on NOTCH1 expression. In the rescue experiment, dual‐luciferase reporter assays revealed that FMO2 knockdown enhanced *NOTCH1* transcriptional activity, which was suppressed by N‐acetylornithine supplementation (Figure S10J, Supporting Information).

Consistently, ATF3 silencing neutralized the proangiogenic effect of N‐acetylornithine in ECs in vitro, as shown by a significant reduction in tube length in the si‐ATF3 group compared to the control group, even after N‐acetylornithine treatment (Figure 6K,L). Furthermore, the deficiency of ATF3 in ECs efficiently limited the effects of N‐acetylornithine or FMO2 overexpression in reducing NOTCH1 and NICD expression (Figure 6M–P, Figure S10K,L, Supporting Information). We observed markedly downregulated endothelial ATF3 protein expression in the ischemic microenvironment post‐MI and HLI (Figure S10M–P, Supporting Information), suggesting that ischemia‐induced ATF3 deficiency may exacerbate NOTCH1 hyperactivation in disease contexts. To sum up, we demonstrated that N‐acetylornithine negatively modulates NOTCH1 expression through the transcriptional regulation of ATF3.

### 2.7 Proangiogenic Effect of FMO2 and N‐acetylornithine in Human Ischemic Diseases

**Figure 7:**
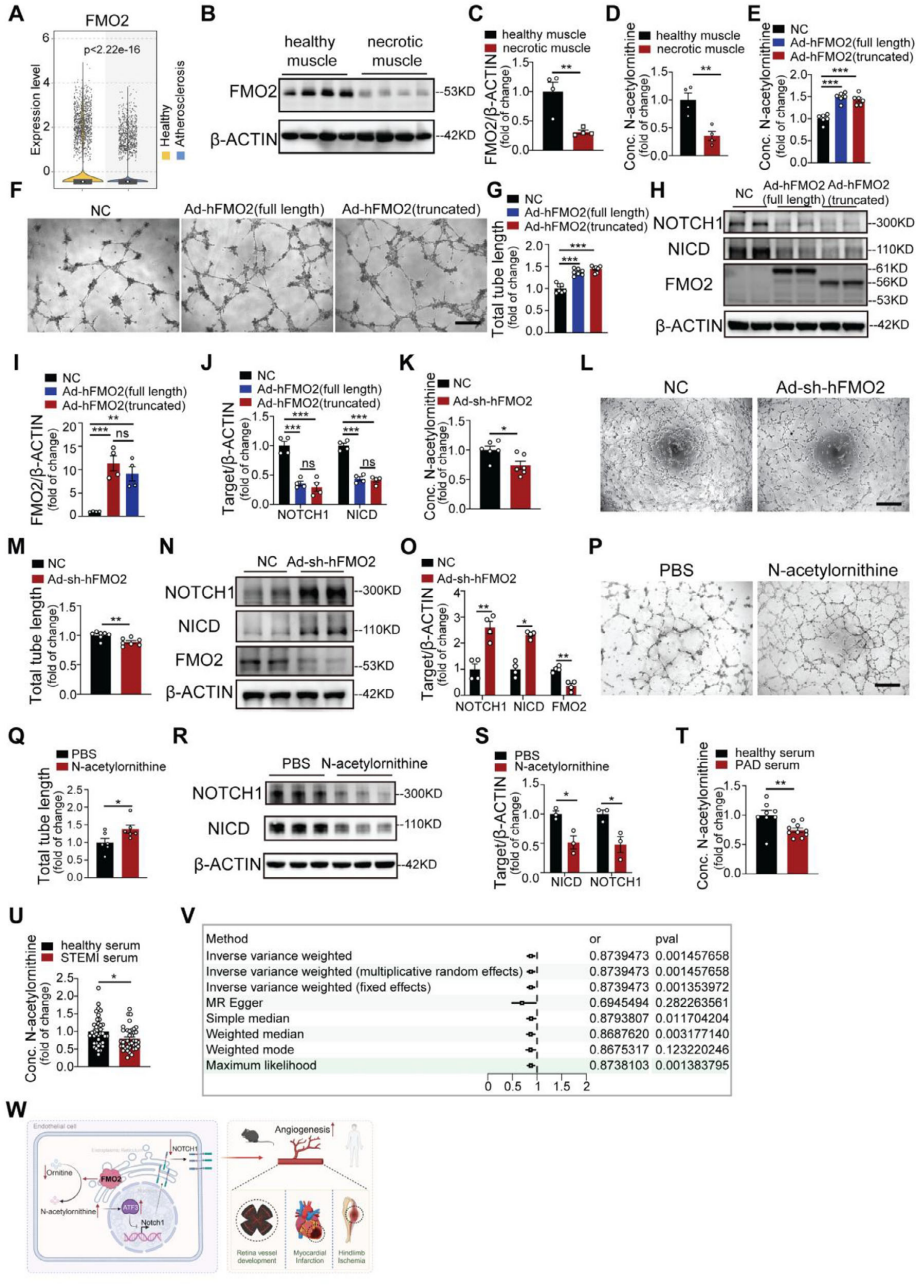
Clinical relevance and utilization of FMO2 and N‐acetylornithine in primary human endothelial cells and ischemic diseases. A) Single‐cell RNA‐seq analysis showing reduced *Fmo2* transcript levels in atherosclerotic plaque tissues compared to healthy vascular tissues. B,C) Protein levels of FMO2 were measured in healthy human gastrocnemius tissue and necrotic tissue from patients with PAD subjected to amputation (*n =* 4 per group). D) Quantitative analysis of N‐acetylornithine levels by UHPLC–MS/MS in healthy human gastrocnemius tissue and necrotic tissue from patients with PAD (*n =* 4 per group). E) Quantitative analysis of N‐acetylornithine levels by UHPLC–MS/MS in primary HUVECs infected with NC, Ad‐hFMO2(full length), or Ad‐hFMO2(truncated) (*n =* 6 per group). F,G) Representative images of tube formation and quantitative statistics of the total tube length of primary HUVECs infected with NC, Ad‐hFMO2(full length), or Ad‐hFMO2(truncated) (*n =* 7 per group). H–J) Immunoblots showing FMO2, NICD and NOTCH1 protein expression in primary HUVECs infected with NC, Ad‐hFMO2(full length), or Ad‐hFMO2(truncated) (*n =* 4 per group). K) Quantitative analysis of N‐acetylornithine levels by UHPLC–MS/MS in primary HUVECs infected with NC or Ad‐sh‐hFMO2 (*n =* 6 per group). L,M) Representative images of tube formation and quantitative statistics of the total tube length of primary HUVECs infected with NC or Ad‐sh‐hFMO2 (*n =* 7 per group), Scale bar, 500 µm. N,O) Immunoblots showing FMO2, NICD and NOTCH1 protein expression in primary HUVECs infected with NC or Ad‐sh‐hFMO2 (*n =* 4 per group). P,Q) Representative images and total tube length of tube networks formed by primary HUVECs treated with PBS and N‐acetylornithine (*n =* 6 per group). Scale bar, 200 µm. R,S) Immunoblots showing NICD and NOTCH1 protein expression following PBS or N‐acetylornithine treatment of primary HUVECs (*n =* 3 per group). T) Quantitative analysis of N‐acetylornithine levels by UHPLC–MS/MS in the serum of healthy controls (*n =* 8) and patients with PAD (*n =* 10). U) Quantitative analysis of N‐acetylornithine levels by UHPLC–MS/MS in the serum of healthy controls (*n =* 36) and patients with STEMI (*n =* 39). V) Analysis of the odds ratio between N‐acetylornithine and clinical outcomes of patients with myocardial infarction. W) Schematic illustration of the current study. Quantified data are presented as mean ± SEM. Unpaired two‐tailed Student's *t*‐test was conducted in C, D, K, M, O, Q, S, T, and U. One‐way ANOVA followed by Tukey's post hoc multiple comparisons test was conducted in E, G, I, and J. ns *p >* 0.05, * *p <* 0.05, ** *p <* 0.01, and *** *p <* 0.001.

To establish the clinical translational potential of our findings, we analyzed single‐cell RNA sequencing data from a comprehensive human atherosclerotic plaque atlas.^[^ ^26^ ^]^ Consistent with our experimental observations, endothelial *Fmo2* expression was significantly downregulated in atherosclerotic plaques compared to that in matched normal vascular tissues (**Figure** **7**A), suggesting its potential role in atherosclerotic diseases. To explore the pathophysiological role of FMO2 in human ischemic diseases, we collected gastrocnemius muscle tissue from necrotic and non‐ischemic healthy parts from patients with peripheral arterial disease (PAD) subjected to amputation. Strikingly, the necrotic muscle from patients with PAD exhibited approximately a 70% decrease in FMO2 proteins relative to healthy tissue (Figure 7B,C, Table S11, Supporting Information). Consistently, the concentration of N‐acetylornithine in the necrotic muscle of patients with PAD was only ≈40% of that in healthy parts (Figure 7D), indicating the general alteration pattern of FMO2 and N‐acetylornithine from rodents to humans under ischemic conditions.

To determine the clinical potential of FMO2 and N‐acetylornithine as therapeutic approaches, we isolated primary human umbilical vein endothelial cells (HUVECs) and treated them with adenovirus‐mediated FMO2 overexpression (Ad‐hFMO2) or vector control (NC). Because a polymorphic allele of FMO2 has been reported in humans that expresses a truncated isoform with a 64 amino‐acid deletion at its C‐terminus,^[^ ^27^ ^]^ we constructed another adenovirus harboring the human truncated FMO2 form simultaneously (Ad‐hFMO2‐truncated). As shown in the data, elevated FMO2, both in its full length and truncated forms, was sufficient to increase the cellular content of N‐acetylornithine (Figure 7E), accompanied by substantially augmented tube formation of primary HUVECs (Figure 7F,G). Overexpression of human FMO2 also inhibited the expression of NOTCH1 and NICD in primary HUVECs (Figure 7H–J). Conversely, knocking down FMO2 in HUVECs using adenovirus (Ad‐sh‐hFMO2) led to decreased N‐acetylornithine levels (Figure 7K), sparser tube formation (Figure 7L,M), and elevated NOTCH1 and NICD expression (Figure 7N,O) compared with the vector group. To further evaluate the significance of FMO2 enzymatic activity in promoting angiogenesis, we generated a human FMO2 overexpression adenovirus with mutations in the enzymatic region (Ad‐mut‐hFMO2) and transduced primary HUVECs with it. These results implied that overexpression of FMO2 without enzymatic activity did not alter the N‐acetylornithine concentration, tube formation capacity, or NOTCH1 and NICD expression in primary HUVECs compared to the vector control (Figure S11A–E, Supporting Information). Furthermore, the addition of N‐acetylornithine enhanced the tube formation ability of primary HUVECs (Figure 7P,Q), accompanied by reduced expression of NOTCH1 and NICD (Figure 7R,S). Moreover, ATF3 silencing in HUVECs efficiently limited the effects of N‐acetylornithine on NOTCH1 and NICD expression. (Figure S11F–I, Supporting Information). N‐acetylornithine treatment of HUVECs following ATF3 silencing also revealed impaired angiogenesis compared with N‐acetylornithine treatment with scramble siRNA alone via tube formation assay (Figure S11J,K, Supporting Information), indicating the necessity of ATF3 downstream of N‐acetylornithine in NOTCH1 regulation and proangiogenic effect in human ECs.

Finally, we evaluated the N‐acetylornithine content in the blood serum of patients diagnosed with PAD or previous STEMI and found that the N‐acetylornithine content was significantly reduced in the serum of patients with PAD and STEMI compared with normal controls with non‐stenosis in the peripheral or coronary arteries (Figure 7T,U, Table S12 and S13, Supporting Information), suggesting that N‐acetylornithine may serve as an important serum biomarker of ischemic diseases. To further confirm the clinical relevance of N‐acetylornithine content and the outcome of ischemic disease, we analyzed the odds ratio of two publicly accessible datasets using Mendelian randomization. The results indicated that N‐acetylornithine protects against adverse clinical outcomes in patients with MI (Figure 7V).

In conclusion, we uncovered a novel proangiogenic role of FMO2 through the metabolic regulation of N‐acetylornithine by transcriptionally inhibiting the ATF3‐NOTCH1 pathway (Figure 7W), highlighting the translational relevance of FMO2 and its metabolite N‐acetylornithine as novel therapeutic targets for treating ischemic diseases.

## 3 Discussion

The importance of metabolism in ECs extends beyond energy production and biosynthesis, regulating signaling pathways and transcriptional programs essential for both angiogenesis and vascular homeostasis.^[^ ^28^, ^29^, ^30^ ^]^ Metabolites can directly influence gene expression by modulating the activities of TFs or epigenetic regulators. In the current study, we identified FMO2‐regulated N‐acetylornithine as an upstream regulator of ATF3, a transcription factor involved in stress responses and vascular remodeling,^[^ ^31^ ^]^ and it affects the expression of its downstream target NOTCH1, resulting in a proangiogenic effect under ischemic conditions. In contrast, EC‐specific FMO2 knockout mice exhibited impaired angiogenic capacity during ischemia and developmental stages. The angiogenic effects were enhanced by compensating for FMO2 or N‐acetylornithine in mice or human ECs. The concentration of N‐acetylornithine has also been validated to strongly correlate with ischemic disease conditions in patients with PAD and STEMI. Therefore, our findings elucidate the functional importance and translational potential of FMO2 and a previously uncharacterized metabolite, N‐acetylornithine, which can be exploited for future therapeutic development.

FMO2 is an enzyme known for its role in xenobiotic metabolism and drug detoxification.^[^ ^19^, ^32^, ^33^ ^]^ However, recent studies have suggested additional functions for FMO2, including the metabolism of endogenous substrates.^[^ ^34^, ^35^ ^]^ Our findings confirmed the metabolic conversion exerted by FMO2 to produce N‐acetylornithine, which harnesses the downstream molecular signaling that contributes significantly to angiogenesis. These consequences suggest that metabolism in ECs is not only essential for cellular energy production and biosynthesis but also plays a critical role in regulating signaling pathways and transcriptional programs involved in multiple biological processes under pathological and physiological conditions. Metabolites such as N‐acetylornithine can also exert non‐metabolic functions by modulating the activity of transcription factors such as ATF3, downregulating NOTCH1 signaling, and potentially impacting vascular remodeling and disease processes.

In contrast, our metabolomics data revealed that FMO2 compensation reduced the ornithine content while enhancing N‐acetylornithine levels. This finding suggests that FMO2 may promote the conversion of ornithine to N‐acetylornithine. OAT plays an important role in the metabolic pathway from ornithine to N‐acetylornithine. The OAT protein surface contains multiple cysteine residues, which are potential sites for oxidative modifications. Given that FMO2 functions as an oxidation‐reduction‐related enzyme,^[^ ^36^ ^]^ it is plausible that FMO2 expression may modify the cysteine residues in OAT, enhancing its catalytic activity. This could promote the conversion of ornithine to N‐acetylornithine, leading to the metabolic changes observed in our study. These findings provide a precedent for the potential role of FMO2 in modulating OAT activity via oxidative modifications. However, future biochemical studies focusing on the direct interaction between FMO2 and OAT are necessary to validate our proposed mechanism.

The translational significance of metabolites in ischemic diseases lies in their potential as novel therapeutic targets or biomarkers for improving diagnosis, prognosis, and treatment outcomes.^[^ ^37^ ^]^ Building on previous relevant studies, the identification of N‐acetylornithine as a regulator of EC function in ischemic disease pathways presents promising prospects for clinical application. In the context of MI or PAD, quantification of N‐acetylornithine levels in serum or tissue samples may serve as a diagnostic or prognostic biomarker. Further clinical studies correlating N‐acetylornithine levels with infarct size, limb salvage rates, angiographic outcomes or treatment response may validate its utility as a biomarker and inform clinical decision‐making.^[^ ^38^, ^39^, ^40^, ^41^ ^]^ Moreover, although the therapeutic potential of N‐acetylornithine has not been reported previously, our studies in MI and HLI models validate its role as a therapeutic modulator. By integrating N‐acetylornithine‐related findings with other relevant studies on EC heterogeneity, metabolic reprogramming, and angiogenic signaling pathways, we provide a comprehensive framework for developing targeted therapeutic interventions. In addition, based on N‐acetylornithine, it is possible to investigate low‐molecular‐weight compounds for adopting clinical indications in ischemic diseases such as MI and PAD. However, the clinical translation of N‐acetylornithine‐based interventions requires rigorous evaluation of efficacy and safety in preclinical and clinical settings, with integration into existing therapeutic approaches for ischemic disease management. By leveraging insights from previous studies and emphasizing both efficacy and safety considerations, N‐acetylornithine‐based therapies offer promising prospects for improving outcomes in patients with ischemic disease like MI and PAD.

Our investigation revealed the essential role of FMO2 in the intricate orchestration of angiogenesis, particularly through its inhibition of the NOTCH1 pathway. The Notch signaling cascade, a fundamental regulator of angiogenesis, orchestrates various pivotal aspects of vascular development, including EC fate determination and vessel sprouting.^[^ ^42^ ^]^ By scrutinizing ECs lacking FMO2, we observed a notable increase in NOTCH1 pathway activity, indicating a possible involvement of FMO2 in modulating NOTCH1 signaling. This heightened NOTCH1 activity in FMO2‐deficient ECs suggests a nuanced interplay between FMO2 and NOTCH1 signaling, which influences the behavior of ECs crucial for sprouting during angiogenesis. Our findings delve into the complexity of vascular development and remodeling, shedding light on a previously unrecognized regulatory mechanism mediated by FMO2. This discovery adds a layer of complexity to our understanding of the molecular pathways governing angiogenesis, opening avenues for further exploration of the precise mechanisms underlying vascular development and the potential therapeutic implications for angiogenesis‐related disorders.

## 4 Conclusion

Our study identified FMO2 as a novel proangiogenic regulator. Targeted ablation of FMO2 in ECs impairs vessel sprouting, whereas compensation of FMO2 significantly enhances the angiogenic process. By comprehensively combining metabolomics and single‐cell sequencing, we uncovered that FMO2 and N‐acetylornithine can regulate the ATF3‐NOTCH1 axis in a non‐classical manner, eventually promoting angiogenesis under ischemic conditions. The therapeutic utilization of FMO2 and N‐acetylornithine can be replicated in human ischemic disease model, and the N‐acetylornithine concentration has been validated to have a strong relationship with the prognosis of patients with ischemic diseases. These findings collectively implicate FMO2 and N‐acetylornithine in future therapeutic discoveries for ischemic diseases.

## 5 Experimental Section

### Animals and Ethics Statement

All experiments with live animals were approved by the Institutional Animal Care and Use Committee of Second Affiliated Hospital, Zhejiang University School of Medicine (Approval No. 2025–189), and were performed according to the National Institutes of Health Guide for the Care and Use of Laboratory Animals (NIH Publication No.85‐23, revised 1996). Male C57BL/6J mice (neonatal pups and 8–12 weeks old) were purchased from Shanghai Slac Laboratory Animal Technology Corporation. Mice were housed under specific pathogen‐free (SPF) conditions with free access to food and water and maintained with a 12:12‐h light/dark cycle. All efforts were made to minimize the number of animals used and their distress.

### Human Sample and Ethics Statement

Surgical samples were obtained from patients who suffered from PAD leading to distal limb necrosis and needed amputation. The necrotic muscle tissue came from the distal necrotic part of the amputation, while the control muscle tissue came from healthy proximal muscles of the amputation. The detailed information of the patient can be found in Table S6, Supporting Information. Sections were frozen in liquid N_2_ and stored at −80 °C until used. Serum samples were obtained from healthy patients as control and patients who suffered from PAD (Table S7, Supporting Information) or STEMI (Table S8, Supporting Information). Sample preparation was done at 4 °C. Sample procurement and preparation were performed according to a human research subject protocol. All subjects were duly informed and written consent by the patient or their relatives. All studies were approved by the Ethics Review Committee from the Second Affiliated Hospital of Zhejiang University (Approval No.2024‐0718).

### Isolation of Adult Gastrocnemius Endothelial Cells

Gastrocnemius muscles were dissected from ischemic and unoperated limbs, respectively. Mice were harvested, pooled, minced with scissors, and then digested in 10 mL collagenase IV (0.2%) at 37 °C for 40 min with pipetting every 10 min. The suspension was then filtered through a 70 µm filter and diluted with an equal volume of washing/blocking buffer (20% FBS in PBS). After centrifugation (400 g, 10 min), the pellet was resuspended in 3 mL RBC lysis buffer for 5 min at RT, then quenched with 30 mL washing buffer and filtered through a 40‐µm filter. Cells were collected by centrifugation (400 g, 10 min). The single‐cell suspension was enriched for ECs by MACS depletion of CD45 positive cells and subsequent enrichment of CD31 positive cells. The CD31^+^CD45^−^ cells were resuspended in an EC‐specific complete culture medium (ECM), and then plated in a 0.1% gelatin‐coated 24 well‐plate at 37 °C with 5% CO_2_.

### Isolation of Neonatal Retinal Endothelial Cells

Eyes from one litter (6 to 9 pups) of 6‐day‐old mice were harvested. The retinas were dissected out aseptically under a dissecting microscope and kept in cold PBS containing 1% penicillin/streptomycin. Retinas (12 to 18 from one litter) were pooled together, minced into small pieces in a 60 mm tissue culture dish using sterilized razor blades, and digested in 5 mL of collagenase type IV (2 mg mL^−1^ in serum‐free DMEM) for 40 min at 37 °C. Following digestion, DMEM with 10% FBS was added and the cellular digests then were filtered through a double layer of sterile 40 µm nylon mesh, centrifuged at 400 g for 10 min, and cells were resuspended with DMEM containing 10% FBS. The CD31^+^CD45^−^ cells of neonatal pups were sorted as described above and were frozen in liquid N_2_ and stored at −80 °C until used.

### Isolation and Culture of Primary Human Umbilical Vein Endothelial Cells

The umbilical vein was rinsed with PBS and injected with pre‐warmed collagenase IV (0.2% wt vol^−1^). After 10 min incubation, the collagenase suspension containing ECs was collected, filtered through a 40 µm nylon cell strainer, and spun down. The ECs were plated on 0.1% gelatin‐coated dishes in M199 medium (1 mg mL^−1^ D‐glucose) supplemented with 20% fetal bovine serum (FBS), 2 mM L‐glutamine, 30 mg L^−1^ endothelial cell growth factor supplements (EGCS), 10 U mL^−1^ heparin, 50 IU mL^−1^ penicillin and 50 µg mL^−1^ streptomycin, or in endothelial basal medium (EBM‐2) supplemented with endothelial growth medium. Primary HUVECs were used between passages 1 and 5 and routinely maintained in 5% CO_2_ at 37 °C.

### N‐Acetylornithine Treatment

For in vivo studies of N‐acetylornithine treatment in MI and IR models, mice were administered with N‐acetylornithine at a dose of 1 mg kg^−1^ body weight via ultrasound‐guided intramyocardial injection. The compound was delivered in a total volume of 30 µL, evenly distributed across three sites (10 µL per site) at the border zone of the infarct. These injections were performed at days 3, 7, 10, and 14 post‐MI/IR surgery. For HLI model, N‐acetylornithine (1 mg kg^−1^ body weight) was administered via intramuscular injection using a microinjector (total volume: 20 µL per injection). The injections were performed at days 1, 3, 5, and 7 after HLI surgery. For in vitro studies, ECs were stimulated with 1 mM N‐acetylornithine for 24 h.

### FITC‐N‐Acetylornithine Treatment

To examine the uptake of N‐acetylornithine by ECs, we synthesized FITC‐conjugated N‐acetylornithine. ECs were cultured with 1 mM FITC‐N‐acetylornithine for 24 h, followed by cell digestion and flow cytometry analysis.

### 5‐FMOrn Dihydrochloride Treatment

ECs were stimulated with 0.5 mM 5‐Fluoromethylornithine (5‐FMOrn) dihydrochloride for 24 h.

### DAPT Treatment

The γ‐secretase inhibitor, DAPT (N‐ [N‐ (3,5‐Difluorophenacetyl) ‐L‐alanyl] ‐S‐phenylglycine t‐butyl ester, 50 µM) was used to inhibit NOTCH1 signaling in ECs in vitro. For in vivo studies in MI or HLI models, DAPT was injected administered via intraperitoneal injection at a dose of 10 mg kg^−1^ of mouse body weight, performed on postoperative days 1, 3, 5, and 7.

### Tube Formation Assay

96‐well plates were coated with 50 µL of growth factor‐reduced Matrigel and incubated for 30 min at 37 °C. ECs were seeded at 20 000 cells per well in a 96‐well plate coated with 50 µL of growth factor‐reduced Matrigel. Where indicated, DAPT or N‐acetylornithine were added to the media. Tube‐like structures was photographed by light microscopy following a 12‐h incubation. The total tube length was calculated using Image‐Pro Plus software.

### Spheroid Sprouting Assay

ECs were suspended in an EGM‐2 medium containing 20% methylcellulose and were incubated overnight in hanging drops to form spheroids. When required, DAPT, N‐acetylornithine, or DMSO was added to this medium. Spheroids were then embedded in collagen gel and cultured for 20 h to induce sprouting as described previously.^[^ ^43^ ^]^ Cultures were fixed with 4% PFA at room temperature and imaged under light microscopy. Analysis of the number of sprouts per spheroid and the total sprout length (cumulative length of sprouts and branches per spheroid) of 8–10 spheroids per group was done on images using the Fiji/ImageJ analysis software.

### Lentivirus Construction and Transfection

For lentivirus‐mediated gene transfer, lentivirus encoding the overexpressed FMO2 gene (LV‐FMO2), FMO2 with the its enzymatic activity crippled (LV‐mut‐FMO2), FMO2 knockdown lentivirus (LV‐shFMO2), and respective negative controls (NC) were purchased from Genechem Co., Ltd. (Shanghai, China). Specifically, the LV‐mut‐FMO2 was constructed through comprehensive deletion of all cofactor‐binding domains, including FAD‐binding sites (residues 9–13, 32, 40–41, and 61–62) and NADP⁺‐binding sites (residues 60–61 and 195–198). To optimize transduction, an MOI of 20–25 was chosen for lentiviral infection. Transfection was performed when cell confluency reached 50–60% in the six‐well plate. After transduction, the cells were replenished with fresh complete culture media 6 h post‐transduction, and experiments were initiated on day 3 or 4. Finally, the efficiency of overexpression and knockdown was evaluated at the protein level by comparison to the negative control vector

### Mosaic Spheroid Sprouting Assay

Control ECs were stained with the CellTracker Red according to the manufacturer's guidelines. And ECs (LV‐FMO2, LV‐shFMO2, and LV‐mut‐FMO2) were generated as described in the Lentivirus transfection strategy section, which fluorescently labeled by GFP. Briefly, control (Red) and treated (GFP) ECs were mixed at an equal ratio (1:1, 200 000 cells in total) and used for spheroid formation and sprouting as described above. Using a Leica DMi8 microscope, 7–10 spheroids were imaged per replicate and per condition. Using the Fiji/ImageJ analysis software package, the contribution to the tip position of each sprout was quantified and represented as the percentage of green (GFP) or red (Red) stained ECs occupying the tip position.

### Co‐Immunoprecipitation

Cells were extracted and lysed in native lysis buffer containing phosphatase and protease inhibitors. After centrifuged for 12 000 rpm, 20 min at 4 °C, the cell lysates were incubated with Anti‐Flag Magnetic Beads overnight at 4 °C. Finally, the beads were washed for three times followed by Western Blot.

### Quantitative RT‐PCR Analysis

EC RNA was extracted from tissue using the Trizol reagent method, and quantified by NanoDrop spectrophotometer.  PCR conditions included initial denaturation at 95 °C for 5 min, 95 °C for 45 s, and 60 °C for 1 min for 45 cycles. Samples were subjected to melting curve analysis to confirm amplification specificity. mRNA expression was analyzed by expressing the cycle threshold (Ct) value as 2‐ΔΔCt, relative to the levels of β‐actin, and further normalized as a fold change to control treatments. All samples were run in duplicate. The primers of genes are listed in Table S14, Supporting Information.

### Chromatin Immunoprecipitation

ChIP was conducted according to the manufacturer's protocol. Briefly, cells were crosslinked with formaldehyde, quenched with glycine, and lysed to isolate nuclei, followed by chromatin fragmentation via sonication. Immunoprecipitate chromatin overnight at 4 °C using antibodies with Protein A/G magnetic beads, including controls (Input, IgG, and positive controls like H3). Finally, reverse crosslinks, purify DNA, and quantify target regions via qPCR. The primers are listed in Table S14, Supporting Information.

### Immunoblotting

Cells were extracted using cell lysis buffer, which contains phosphatase and protease inhibitors. Protein concentration in the supernatant was assessed using the bicinchoninic acid protein assay. Standard sodium dodecyl sulfate‐polyacrylamide gel electrophoresis (SDS‐PAGE) protocols were employed to identify protein expression. Antibodies utilized in this study are listed in Table S15, Supporting Information.

### Dual‐Luciferase Reporter Assay

Cells were lysed with Lysis Buffer, followed by room temperature incubation and centrifugation. For detection, transfer 20 µL cell lysate to a 96‐well plate, first measure Firefly luciferase activity by adding 100 µL Firefly Working Solution, then quench and measure Renilla luciferase activity by adding 100 µL Renilla Working Solution. Accurate transcriptional activity comparison was normalized Firefly signals to Renilla after background subtraction.

### Adenovirus Construction and Transfection

Adenoviruses encoding the following human FMO2 variants were generated by Genechem Co., Ltd. (Shanghai, China): full‐length (Ad‐hFMO2(full length)), truncated (Ad‐hFMO2(truncated)), enzymatically inactive mutant form (Ad‐mut‐hFMO2), and knockdown (Ad‐sh‐hFMO2), alongside their respective negative controls. Specifically, the Ad‐mut‐hFMO2 was constructed through comprehensive deletion of all cofactor‐binding domains, including FAD‐binding sites (residues 9–13, 32, 40–41, and 61–62) and NADP⁺‐binding sites (residues 60–61 and 195–198). An MOI of 20–25 was chosen for transduction. The cells were given a fresh complete culture media 6 h following the transduction and experiments started on day 2 or 3. The efficiency of overexpression was evaluated at the protein level in comparison to a negative control vector.

### *Tie2*‐Promoter Endothelial‐Specific FMO2 Adeno‐Associated Virus Construction

Endothelial‐specific FMO2 AAVsig was generated with a *Tie2* promoter added before the FMO2 sequence to specifically target ECs. The efficiency of FMO2‐overexpression in ECs was evaluated at the protein level in comparison to a negative control vector.

### siRNA Transfection

Small interfering RNAs (siRNA) against *ATF3* in mouse or human were synthesized by GenePharma Co., Ltd (Shanghai, China). The sequences of siRNA are listed in Table S17, Supporting Information. Transfection is performed when there is a 50–60% fusion rate of cells in the six‐well plate. First, 100 nmol siRNA was added to 250 µL of serum‐free growth medium and gently mixed. Second, 5 µL of the mixed Lipofectamine RNAiMAX was added to 250 µL of complete medium, diluted, and incubated for 5 min at room temperature. The diluted siRNA and Lipofectamine RNAiMAX were mixed and incubated at room temperature for 10 min. Subsequently, the original medium in the six‐well plate was removed and 1.5 mL of fresh serum‐free growth medium was added. Finally, the mixture was added to the six‐well plate in a final volume of 2 mL per well with gentle shaking. After transfection, the cells were incubated in 5% CO_2_ at 37 °C for 6 h, after which the serum‐containing medium was replaced and the cells were incubated for 48 h.

### Transgenic Mouse Generation

FMO2^△EC^ Mice: To generate endothelial‐specific FMO2 knockout mice, FMO2^fl/fl^ mice were crossed with Tie2‐Cre mice. For adult HLI: Cre activity was induced in 8‐week‐old mice by 5 consecutive daily intraperitoneal injections of tamoxifen (1 mg per mouse, in corn oil), and all littermates injected with the same dose to generate the necessary controls. HLI in 10‐week‐old mice after tamoxifen treatment as above and littermate animals were used as controls. For the postnatal retinal angiogenesis model: Tamoxifen (1 mg mL^−1^, 50 µL /each injection) is used to inject each pup from P1 to P4. Retinas were harvested at P6.

### Murine MI or IR Model and Subsequent FMO2 Lentivirus Treatment

Murine MI and IR models were conducted according to protocols as previously described.^[^ ^44^, ^45^ ^]^ Mice were anesthetized by intraperitoneal administration of sodium pentobarbital (1%, 60 mg kg^−1^). After confirming anesthesia by pedal reflex loss, mice were intubated and mechanically ventilated. A left thoracotomy at the third intercostal space exposed the left anterior descending (LAD) coronary artery. To establish the I/R model, the LAD artery was occluded transiently using a 7‐0 silk suture threaded through PE‐10 tubing, ≈2 mm from its origin. Myocardial ischemia was verified by visible myocardial blanching, akinesis of the downstream myocardium, and ST‐segment elevation on the electrocardiogram. Following 45 min of ischemia, reperfusion was induced by loosening the suture, evidenced by the return of normal tissue color, restored contractility, and normalization of the ST‐segment on ECG. For MI model, the LAD coronary artery was permanently ligated. Sham‐operated controls underwent identical surgical procedures but without LAD occlusion. Post‐procedural closure involved suturing of the muscle and skin layers sequentially. For in vivo study involving the FMO2 lentivirus in MI model, mice in the NC and LV‐FMO2 groups were treated with 1 × 10^6^ PFU of lentivirus in 25 µL DMEM immediately after ligation. The solution was injected into five sites (5 µL per site): four sites at the border of the infarct and one site at the apex.

### Murine Hindlimb Ischemia Model with AAV‐FMO2 Administration

10‐week‐old C57 mice were anesthetized by intraperitoneal administration of sodium pentobarbital (1%, 60 mg kg^−1^) and placed on a heated blanket to maintain body temperature. Both the proximal portion of the femoral artery and the distal portion of the saphenous artery was ligated with a 7–0 suture. Branches between the two sites were ligated and all arteries in between were excised. Noninvasive laser Doppler imaging was used to assess hindlimb blood flow immediately after undergoing HLI, with subsequent imaging at 0, 3, 7, and 14 days post‐HLI. For in vivo studies with AAV‐FMO2, mice were administered 1 × 10^9^ PFU adeno‐associated virus per mice by microinjector, intramuscular injection with a total volume of 20 µL 4 weeks before HLI.

### Whole‐Mount Immunofluorescence Staining of Retinas

P6 pups were euthanized and the eyes were removed and prefixed in 4% PFA for 30 min at room temperature. The retinas were dissected out and blocked overnight at 4 °C in a blocking buffer (PBS containing 5% FBS, 1% bovine serum albumin, and 1% Triton X‐100). After washing with a washing buffer (1% Triton X‐100 in PBS), the retinas were incubated with Alexa‐conjugated isolectin GS‐IB4 (1:100; Invitrogen) for vessel staining. Neonatal mice were achieved by intraperitoneal injection of DAPT, or PBS once daily from P2 to P4. Retinas were harvested at P5.

Neonatal mice in the FMO2^fl/fl^ group and FMO2^△EC^ group were treated with a total of 1 × 10^9^ PFU adeno‐associated virus (AAV‐NC or AAV‐FMO2) in 10 µL DMEM by intraperitoneal injection from P1 to P4. Retinas were harvested at P5.

### Immunofluorescence Staining

Freshly isolated whole gastrocnemius muscle samples were mounted in O.C.T. compound, placed in an isopentane bath, and cooled in liquid nitrogen. Frozen tissue sections were fixed in 10% formaldehyde for 8 h and permeabilized with 0.5% Triton X‐100 in PBS. After blocking with PBS containing 5% bovine serum albumin, sections were respectively incubated overnight at 4 °C with primary antibodies (1:200), followed by incubation with respective secondary antibodies (1:400). Nuclei were counterstained with DAPI. Images were captured using fluorescence microscope.

### Active Notch1 (NICD) Staining

NICD was visualized by Tyramide Signal Amplification (TSA)‐based immunofluorescence staining.^[^ ^46^ ^]^ Frozen sections were fix with 4% PFA, permeabilize with 0.3% Triton X‐100/PBS containing 5–10% goat serum for 1 h, then incubate with primary antibodies followed by HRP‐conjugated secondary antibodies. Finally, signals were amplified using TSA fluorophores, with DAPI counterstaining for nuclei.

### Histological Examination of Ischemic Limbs

Gastrocnemius muscles obtained from ischemic limbs were subjected to histological assessment 14 days following HLI induction. Following serial sections of the entire muscle were prepared, and hematoxylin‐eosin staining was carried out. Necrotic areas, defined by the presence of necrotic myocytes, inflammatory infiltrates, and interstitial cells, were identified. Quantification of necrotic area fraction (%) was conducted via digital image analysis (Image J).

### Micro‐CT Angiography

Mice were anesthetized with ketamine and xylazine; then, heparinized saline (50 U mL^−1^, 100 µL mouse^−1^) was injected through the peripheral vein. The heart was removed and perfused through the aorta with vasodilator buffer [papaverine (4 mg L^−1^) + adenosine (1 g L^−1^) in PBS] and 4%PFA; then, the Microfil solution (Flow Tech, MV‐122) was perfused into the aortic root and allowed to spread throughout the whole vasculature. The gastrocnemius muscle was collected and fixed in 4% PFA; then, micro‐CT imaging was conducted by using a Scanco micro‐CT 100 scanners. 3D morphometric quantification was performed with Amira 6.0.1 software.

### Single‐cell RNA‐sequencing (scRNA‐seq)—Library Preparation and Sequencing

ECs were MACS‐bead sorted (as described above) from the P6 retina from FMO2^−/−^ group (*n =* 14) and WT group (*n =* 20). Single‐cell suspensions were resuspended in PBS containing 0.04% ultra‐pure BSA. scRNA‐seq libraries were prepared using the Chromium Single Cell 3′ Reagent Kits v3 (10× Genomics; Pleasanton, CA, USA) according to the manufacturer's instructions. Generated libraries were sequenced on an Illumina HiSeq 6000, followed by de‐multiplexing and mapping to the mouse genome using CellRanger (10× Genomics, version 7.1.0).

### In Silico EC Selection

Sample data were aggregated using CellRanger software and raw data were processed further in R (version 4.3.1). The following quality control steps were performed: (i) genes expressed by less than 10 cells or with a row average of < 0.002 were not considered; (ii) cells that expressed fewer than 300 genes (low quality), and cells that expressed over 8000 genes (potential doublets) were excluded from further analysis; (iii) cells in which over 25% of unique molecular identifiers (UMIs) were derived from the mitochondrial genome were removed. The data were normalized using the NormalizeData function as implemented in the Seurat package (version 4.3.0.1, Satija et al., 2015).

After a log‐normalization, Seurat was applied to integrate all data sets with anchor genes, to correct the batch effect among different samples. To perform a principal component analysis (PCA), we selected the top 2000 genes with the highest variability in expression. Subsequently, we utilized the Uniform manifold approximation and projection (UMAP) for dimension reduction based on the top 50 principal components. Graph‐based clustering (resolution 0.5) was performed according to their gene expression profiles as implemented in Seurat. EC clusters were annotated based on the expression of known EC and non‐EC marker genes. Contaminating cell clusters (non‐ECs) were removed, and all downstream analysis was performed on ECs only. Single‐cell transcriptome profiles of mouse heart and gastrocnemius tissue samples were obtained from the Gene Expression Omnibus (GEO) database.

### Functional and Statistical Analysis

As described in previous research, the gene expression, GO pathway enrichment and pseudotime analyses were performed using the SCP R package (version 0.5.1). To identify differentially expressed genes (DEGs) between different identities, FindMarkers (Seurat R Package) with default parameters was used with the Wilcoxon test. The Benjamini–Hochberg method was employed to adjust the p values (adjust‐*P <* 0.05 was considered significant).

### Metabolomics

Accurately prepare 6 parallel replicate samples per group (NC, LV‐shFMO2, LV‐FMO2), and the cells were collected from the petri dishes into 15 mL centrifuge tubes along with the culture medium. Subsequently, the cells were centrifuged at 800 g for 10 min at room temperature using a low‐speed centrifuge. After centrifugation, the supernatant was separated from the cells and placed at −80 degrees Celsius for storage for untargeted metabolomics testing.

### UPLC–MS/MS Determination of N‐acetylornithine

Ultra‐high performance liquid chromatography‐tandem mass spectrometry (UHPLC–MS/MS, AB SCIEX QTRAP 5500) was used for the determination of N‐acetylornithine in muscle tissue, serum and cell lysates. Tissue samples were prepared by using stable‐isotope dilution by mixing 20 µL of tissue with 80 µL of 10 µmol L^−1^ d2‐N‐acetylornithine in MeOH. Serum samples were prepared by using stable‐isotope dilution by mixing 100 µL of serum with 400 µL of 10 µmol L^−1^ d2‐N‐acetylornithine in MeOH. The mixture was then vortexed and mixed, left to stand on ice for 10 min, and centrifuged at 13 000 g (4 °C) for 30 min. The supernatant after centrifugation was collected, dried and then stored at −80 °C for further use. Instrument setting parameters are as follows: for N‐acetylornithine, +175 →+158 m/z; for d2‐N‐acetylornithine, +177 →+160 m/z. The detected peak area was used to calculate the concentration of each target analyte in the sample and normalized using peak area of internal standards.

### Determination of Ornithine

The concentration of ornithine was tested by Mouse Ornithine ELISA Kit purchased by Shanghai COIBO BIO. Cells were collected and then lysed by ultrasonic. After adding standard and sample into wells, 100 µL of HRP‐conjugate reagent was added to each well, and incubated with an adhesive strip for 60 min. Then aspirate each well and wash, repeating the process four times for a total of five washes. Subsequently, chromogen solution A 50 µL and chromogen solution B 50 µL were added to each well. Gently mix and incubate for 15 min. Last, Optical Density (O.D.) at 450 nm was read using a microtiter plate reader within 15 min after adding 50 µL Stop Solution to each well.

### Determination of Ornithine‐Aminotransferase Enzyme Activity

The enzyme activity of OAT was tested by Ornithine aminotransferase activity detection kit purchased by Shanghai mlbio. Cells were collected, counted and finally lysed by ultrasonic on ice. Then detection reagents and samples were added into 96‐well plate. Optical Density (O.D.) at 340 nm was read using a microtiter plate reader immediately after mixing (A1) and 10 min after incubation at 37 °C(A2), respectively. The O.D. difference between two time points (A1‐A2) was used to calculate enzyme activity of OAT and normalized by number of cells.

### Determination of Glutamate

The concentration of glutamate was evaluated by Glutamate Assay Kit purchased from Sigma‐Aldrich. First, cells were homogenized in the Glutamate Assay Buffer and centrifuged at 13 000 g for 10 min to remove insoluble material. Then 100ul of the appropriate reaction mix was added to each of the wells according to the manufacturer's instructions. After incubating the reaction for 30 min at 37 °C, absorbance at 450 nm was measured using a microtiter plate reader.

### Single‐Cell Suspension Preparation and Flow Cytometry

Following euthanasia, tissues were dissected, minced on ice, and enzymatically digested in a buffer containing Dispase II (2 mg mL^−1^), Collagenase IV (2 mg mL^−1^), DNase (1 U mL^−1^), and CaCl_2_ (2 mM) at 37 °C for 25 min with intermittent shaking. The reaction was quenched with 20% FBS in HBSS, and the suspension was sequentially filtered through 100‐µm and 70‐µm strainers to remove debris. Then single‐cell suspensions were blocked and incubated with fluorescently labeled anti‐mouse antibodies at RT for 20 min. After washing with PBS containing 2% BSA, the cells were analyzed on a BD LSR II flow cytometer or sorted using the BD FACS Aria II cell sorter according to the manufacturer's protocol. Antibodies utilized in this study are listed in Table S15, Supporting Information.

### Statistical Analysis

GraphPad prism 8 was used for statistical analyses and graph generation. All data were presented as mean ± SEM for all experiments. The normality of data was assessed using Shapiro‐Wilk test. The exact sample size (*n*), representing independent biological replicates, is provided in each figure legend. Unless otherwise noted, differences between two groups were analyzed using unpaired two‐tailed Student's *t*‐test, whereas multi‐group comparisons employed one‐way ANOVA (for one factor) or two‐way ANOVA (for two factors) followed by Tukey's post hoc multiple comparisons test. *p <* 0.05 was considered statistically significant. (ns *p >* 0.05, * *p <* 0.05, ** *p <* 0.01, and *** *p <* 0.001).

## Conflict of Interest

The authors declare no conflict of interest.

## Author Contributions

J.W., Y.X., and X.W. contributed equally to this work. C.N. and X.H. designed and analyzed all experiments. J.W., Y.X. and Y.C performed in vivo experiments. X.W. Z.Z. and Y.R. performed in vitro experiments. M.L. and Z.F. performed bioinformatics analysis and implemented the online databases. C.X. provided human samples. Q.L. and J.Z. prepared scRNA‐seq samples. M.L. processed scRNA‐seq data. Z.F. analyzed the metabolomics database. W.Z., J.N. and J.C. provided advice and discussed results. C.N. and X.H. wrote the manuscript. X.H. conceptualized the study. All authors have read and approved the article.

## Supporting information



Supporting Information

## Data Availability

The data that support the findings of this study are available in the supplementary material of this article.
