# Supplementary material for: FMO2 Promotes Angiogenesis via Regulation of N‐Acetylornithine
Source: Adv Sci (Weinh). 2025 Oct 6;12(48):e06618. doi: 10.1002/advs.202506618 (PMC12752610; doi:10.1002/advs.202506618)
Supplement: Supplementary file 1 — Supporting Information [file ADVS-12-e06618-s001.docx]

Supplementary Materials for

**FMO2 Promotes Angiogenesis via Regulation of N-acetylornithine**

Jingyi Wang *et al.*

*Corresponding author. Email: hxy0507@zju.edu.cn

**This PDF file includes:**

Figs. S1 to S11

Tables S1 to S17

Figure S1. Reduced endothelial FMO2 expression in ischemic diseases

Figure S2. Global FMO2 depletion impairs angiogenesis

Figure S3. ​Endothelial-Specific FMO2 knockout attenuates angiogenesis in ischemia models

Figure S4. Overexpression of FMO2 promotes angiogenesis

Figure S5. FMO2 promotes angiogenesis by inhibiting NOTCH1

Figure S6. DAPT restores angiogenesis in FMO2^△EC^ ischemic mice

Figure S7. Expression of VEGFA, VEGFC, CXCR4 and DLL4 following FMO2 modulation both *in vivo* and *in vitro*.

Figure S8. FMO2 promotes angiogenesis via N-acetylornithine regulation

Figure S9. N-acetylornithine promotes angiogenesis in ischemic diseases

Figure S10. N-acetylornithine inhibits NOTCH1 to promote angiogenesis by regulating ATF3

Figure S11. Pro-angiogenic effect of FMO2 and N-acetylornithine in human ischemic diseases

Table S1. Top 20 downregulated genes in whole heart cells and ECs post MI

Table S2. Flow ratio values in WT and FMO2^-/-^ mice subjected to HLI

Table S3. Echocardiography values in FMO2^△EC^ and FMO2^fl/fl^ mice subjected to MI

Table S4. Flow ratio values in FMO2^△EC^ and FMO2^fl/fl^ mice subjected to HLI

Table S5. Flow ratio values in FMO2^△EC^ and FMO2^fl/fl^ mice following AAV-NC or AAV-FMO2 injection in HLI model

Table S6. Flow ratio values in FMO2^△EC^ and FMO2^fl/fl^ mice following vehicle or DAPT treatment in HLI model

Table S7. Echocardiography values in FMO2^△EC^ and FMO2^fl/fl^ mice following vehicle or DAPT treatment in MI model

Table S8. List of compounds detected in LV-FMO2 or LV-shFMO2 groups through pairwise comparisons

Table S9. Flow ratio values in FMO2^△EC^ and FMO2^fl/fl^ mice following PBS or N-acetylornithine treatment in HLI model

Table S10. Echocardiography values in FMO2^△EC^ and FMO2^fl/fl^ following PBS or N-acetylornithine treatment in MI model

Table S11. Baseline characteristics of patients with PAD subjected to amputation

Table S12. Baseline characteristics of patients with PAD and healthy controls

Table S13. Baseline characteristics of patients with STEMI and healthy controls

Table S14. Primers

Table S15. Primary antibodies

Table S16. Chemicals and Reagents

Table S17. Oligonucleotides


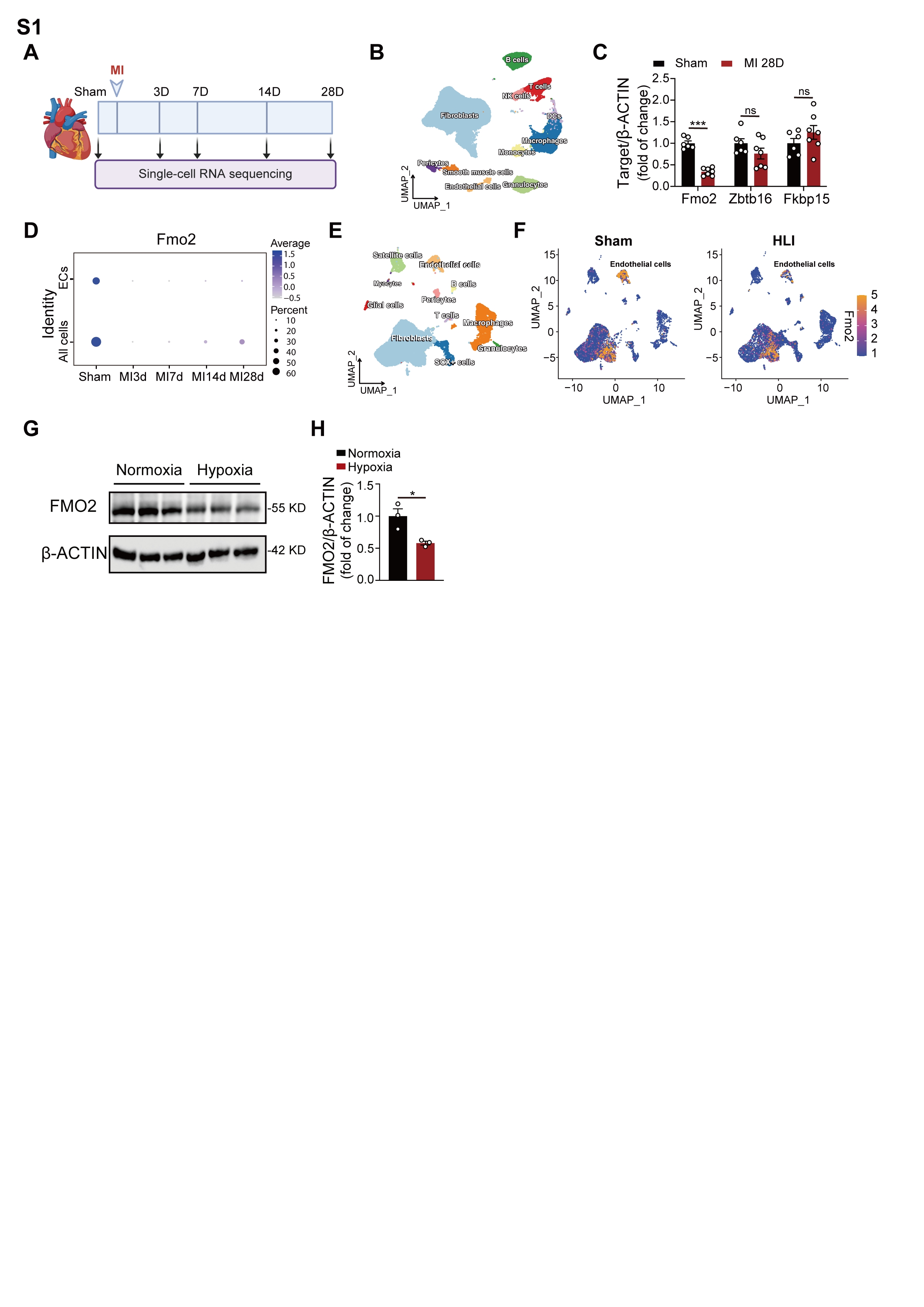


**Figure S1.** **Reduced endothelial FMO2 expression in ischemic diseases**

**(A)** Schematic flow of single-cell sequencing from murine hearts subjected to MI.

**(B)** The uniform manifold approximation projection (UMAP) of combined cells revealed 11 clusters based on transcriptional similarity from murine hearts.

**(C)** The mRNA level of *Fmo2*, *Zbtb16* and *Fkbp15* in the heart in sham (*n =* 6) and MI groups (*n =* 7).

**(D)** The expression of *Fmo2* displayed reduction after MI and sustained to 28 days in all cells and ECs according to sc-RNA seq.

**(E)** The UMAP of combined cells revealed 11 clusters based on transcriptional similarity from murine gastrocnemius.

**(F)** Expression of *Fmo2* in all clusters in sham (left) and HLI (right) groups.

**(G-H)** Western blot analysis of FMO2 expression in endothelial cells under normoxic or hypoxic conditions (*n =* 3 per group).

Quantified data are presented as mean ± SEM. Unpaired two-tailed Student’s *t*-test was conducted in **C** and **H**. ns *P >* 0.05, * *P <* 0.05, ** *P <* 0.01, and *** *P <* 0.001.

**
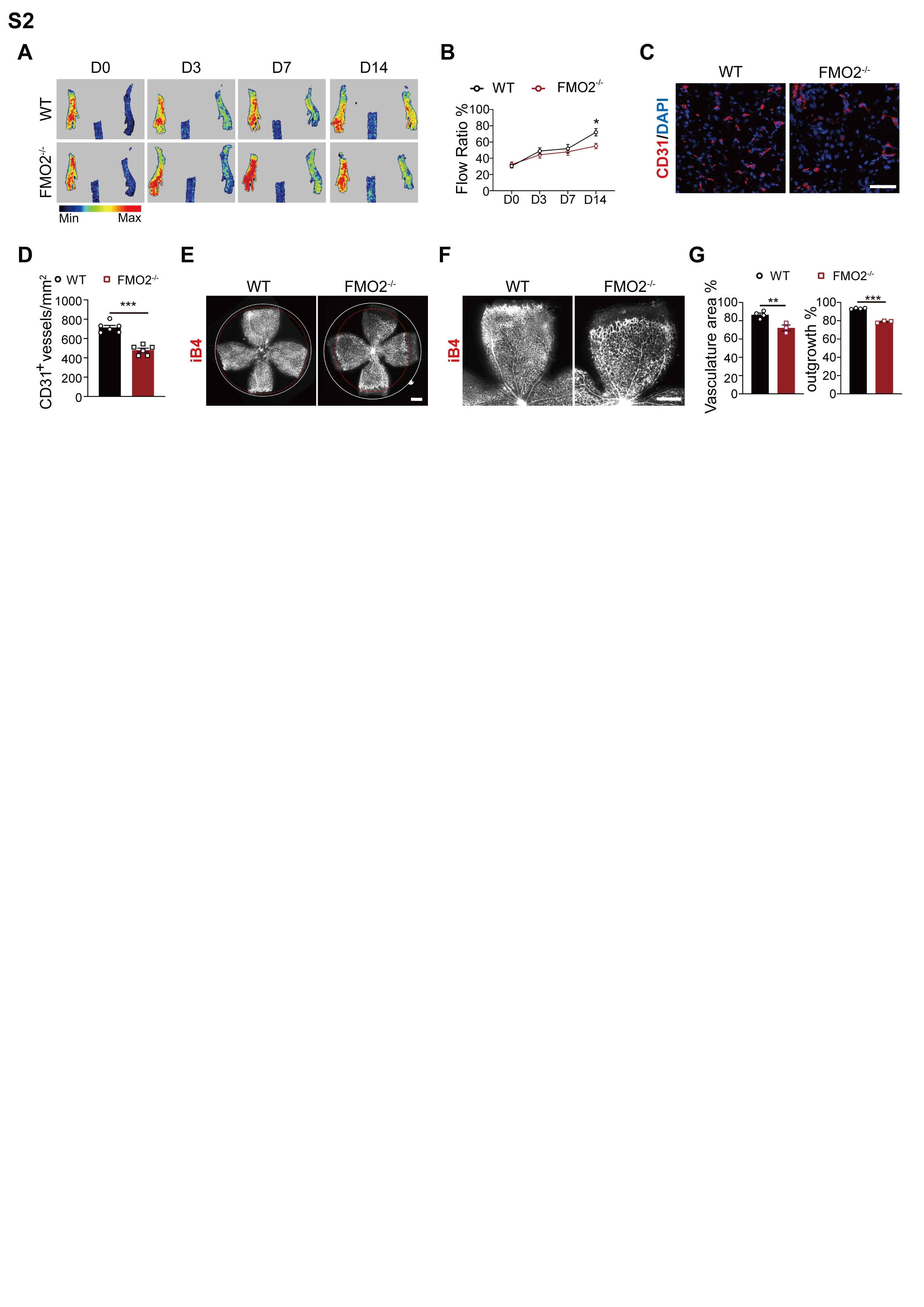
**

**Figure S2. ​Global FMO2 Depletion Impairs Angiogenesis**

**(A-B)** HLI was surgically induced in 10-week-old FMO2^-/-^ mice and their littermates. On Day 0, Day 3, Day 7, and Day 14 after HLI, blood flow was evaluated via laser Doppler imaging and quantified for each animal as the ratio of measurements of the injured (HLI) and uninjured (non-HLI) limbs (WT group, *n =* 9; FMO2^-/-^ group, *n =* 12) (* *P <* 0.05, FMO2^-/-^ vs. WT)

**(C-D)** Immunofluorescence staining for CD31 in gastrocnemius muscles on FMO2^-/-^ mice and their littermates 14 days after subjected to HLI. scale bar, 50 μm. Quantification of CD31 positive vessels per mm^2^ (*n =* 6 per group).

**(E-G)** Whole-mount P6 retinas from WT or FMO2^-/-^ pups were probed for isolectin B4 (iB4), and statistical summary of vascular area and length of retinas. Scale bars, 800 μm. (WT group, *n =* 4; FMO2^-/-^ group, *n =* 3)

Quantified data are presented as mean ± SEM. Unpaired two-tailed Student’s *t*-test was conducted in **D** and **G**. Two-way ANOVA followed by Tukey’s post hoc multiple comparisons was conducted in **B**. ns *P >* 0.05, * *P <* 0.05, ** *P <* 0.01, and *** *P <* 0.001.


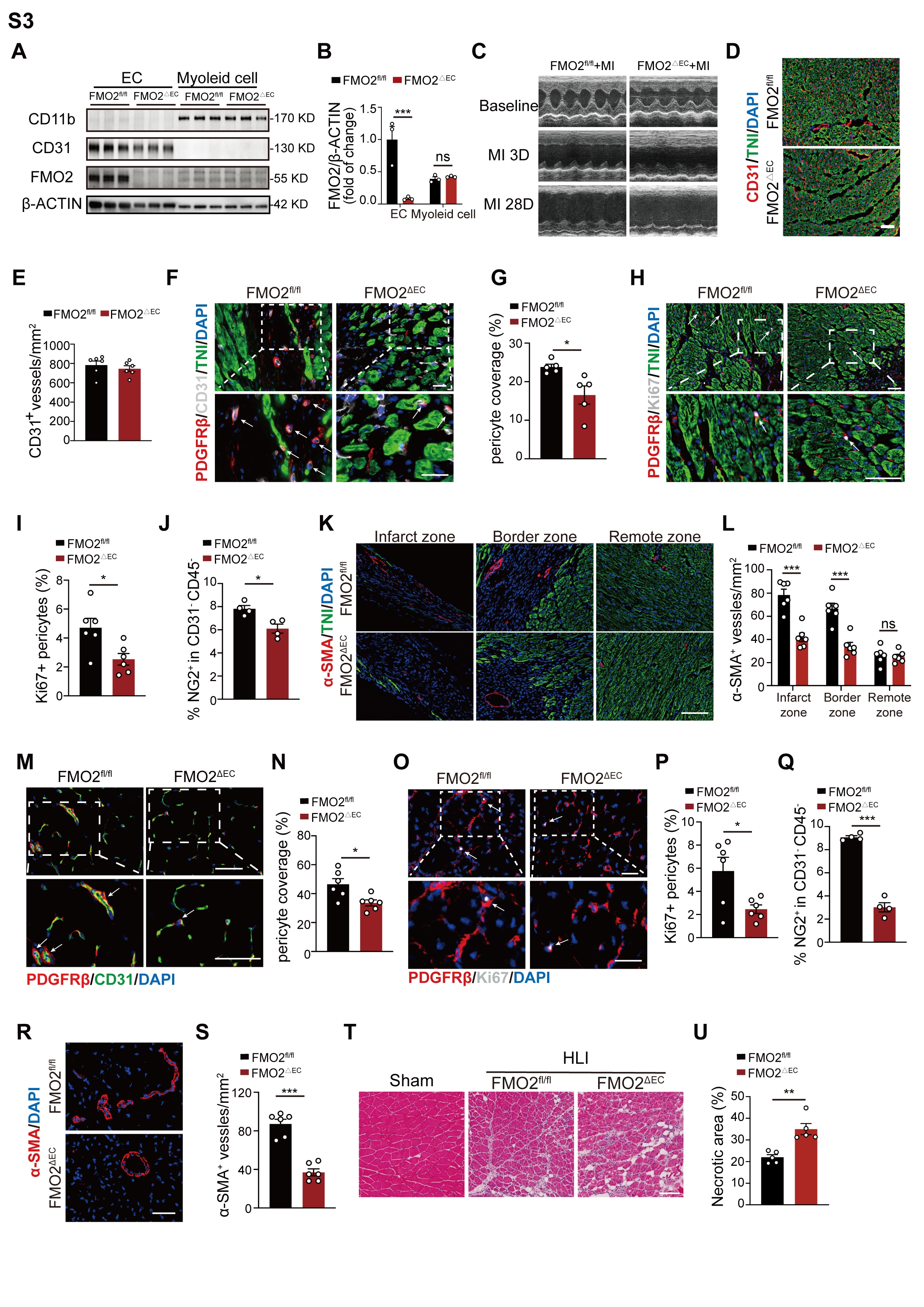


**Figure S3. ​Endothelial-Specific FMO2 knockout attenuates angiogenesis in ischemia models**

**(A-B)** Western blot analysis of FMO2 expression in ECs and myeloid cells from FMO2^△EC^ and control mice (*n =* 3 per group).

**(C)** Representative cardiac ultrasound M-mode images of FMO2^△EC^ mice and FMO2^fl/fl^ mice at baseline, 3 and 28 days after myocardial infarction.

**(D-E)** Immunostaining of endothelial marker CD31 on sections from the remote zone of FMO2^△EC^ mice and FMO2^fl/fl^ mice post-MI, cardiomyocytes were stained by cardiac troponin I (TNI), and nuclei were co-stained with DAPI. Quantification of CD31 positive vessels per mm^2^ was shown in **(E)** (*n =* 6 per group). Scale bar, 50 μm.

**(F-G)** Representative immunofluorescence images showing PDGFRβ (red), CD31 (green) and DAPI (blue) staining in myocardial infarction tissues from FMO2^fl/fl^ and FMO2^△EC^ mice **(F)**, with the statistical analysis of pericyte coverage rates, indicated by the proportion of CD31^+^ vessels covered by PDGFRβ^+^ pericytes, shown in **(G)** (*n =* 5 per group). Scale bar, 50 μm.

**(H-I)** Representative immunofluorescence images showing PDGFRβ (red), Ki67 (white) staining in myocardial infarction tissues from FMO2^fl/fl^ and FMO2^△EC^ mice. Cardiomyocytes were visualized by staining for cardiac troponin I (TNI), and nuclei were counterstained with DAPI. The percentage of Ki67^+^ pericytes of total pericytes were quantified in **(I)** (*n =* 6 per group). Scale bar, 50 μm.

**(J)** Flow cytometry analysis of NG2^+^ pericyte proportion in CD31^-^CD45^-^ population from FMO2^fl/fl^ and FMO2^△EC^ infarcted myocardium post-MI (*n =* 4 per group).

**(K-L)** Representative immunofluorescence images showing α-SMA (red) on sections from the infarct**,** border and remote zones of FMO2^△EC^ mice and FMO2^fl/fl^ mice post-MI, cardiomyocytes were stained by cardiac troponin I (TNI) and nuclei were co-stained with DAPI. Quantification of α-SMA positive vessels per mm^2^ was shown in **(L)** (*n =* 6 per group). Scale bar, 50 μm.

**(M-N)** Representative immunofluorescence images showing PDGFRβ (red), CD31 (green) and DAPI (blue) staining in gastrocnemius tissues post-HLI from FMO2^fl/fl^ and FMO2^△EC^ mice **(M)**, with the statistical analysis of pericyte coverage rates shown in **(N)** (*n =* 6 per group). Scale bar, 50 μm.

**(O-P)** Representative immunofluorescence images showing PDGFRβ (red) and Ki67 (white) staining in hindlimb ischemia tissues from FMO2^fl/fl^ and FMO2^△EC^ mice **(P)**. The percentage of Ki67^+^ pericytes of total pericytes were quantified in **(P)** (*n =* 6 per group). Scale bar, 50 μm.

**(Q)** Flow cytometry analysis of NG2^+^ pericyte proportion in CD31^-^CD45^-^ population from FMO2^fl/fl^ and FMO2^△EC^ gastrocnemius tissues post-HLI (*n =* 4 per group).

**(R-S)** Representative immunofluorescence images showing α-SMA (red) staining in gastrocnemius tissues post-HLI from FMO2^fl/fl^ and FMO2^△EC^ mice **(R)**, with the statistical analysis of α-SMA⁺ vessel density shown in **(S)** (*n =* 6 per group). Scale bar, 50 μm.

**(T-U)** Histological analysis of necrotic area fraction from FMO2^△EC^ and FMO2^fl/fl^ mice in gastrocnemius tissues post-HLI (*n =* 5 per group).

Quantified data are presented as mean ± SEM. Unpaired two-tailed Student’s *t*-test was conducted in **E**, **G**, **I**, **J**, **N**, **P**, **Q**, **S** and **U**. Two-way ANOVA followed by Tukey’s post hoc multiple comparisons was conducted in **B** and **L**. ns *P >* 0.05, * *P <* 0.05, ** *P <* 0.01, and *** *P <* 0.001.


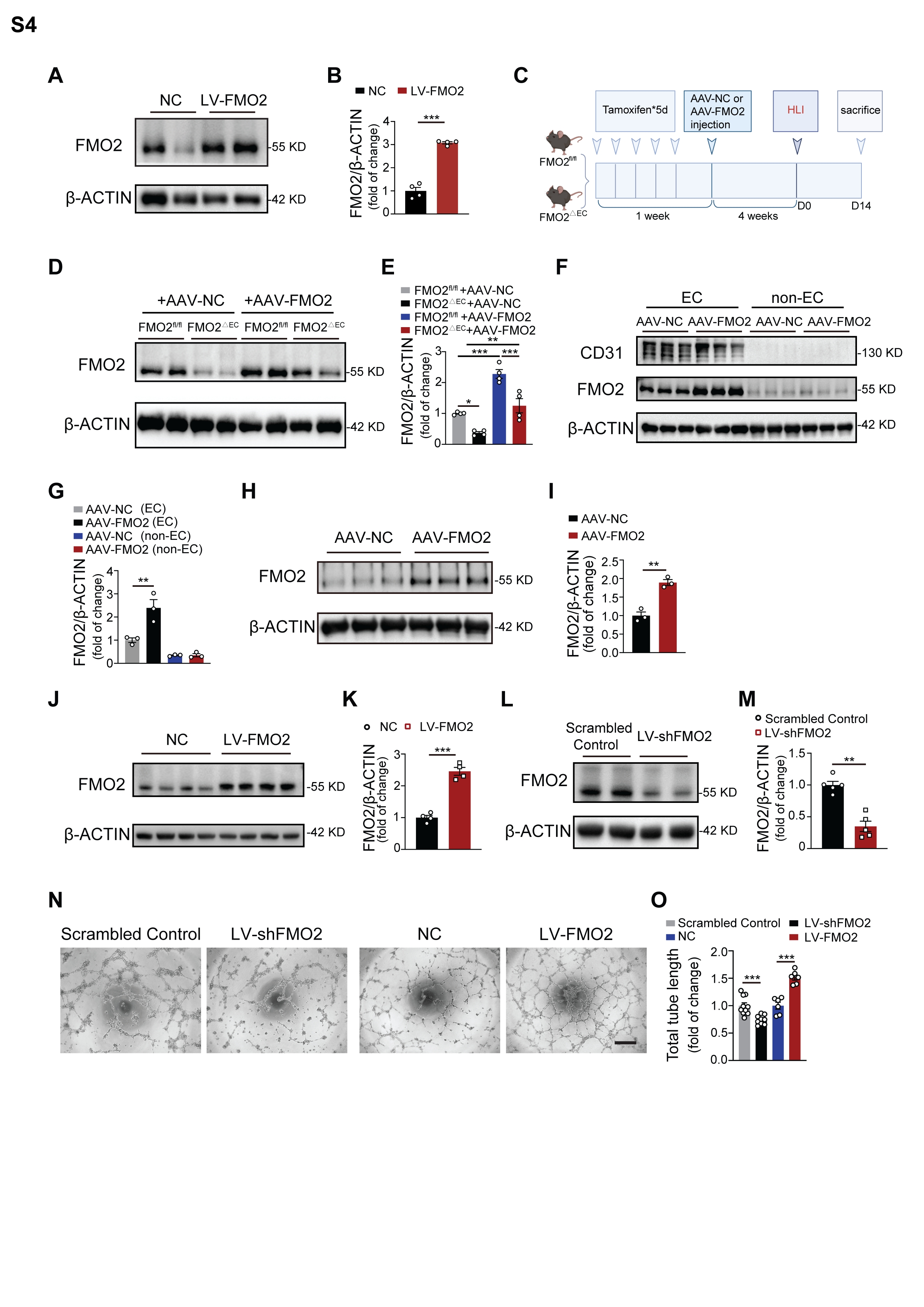


**Figure S4. Overexpression of FMO2 promotes angiogenesis**

**(A-B**) Western blot analysis of FMO2 expression in mouse hearts at 28 days after injection of the lentiviral vector overexpressing FMO2, and quantitative analysis were plotted in **(B)** (*n =* 4 per group).

**(C)** Schematic flow diagram of HLI model following AAV-NC or AAV-FMO2 injections in FMO2^fl/fl^ and FMO2^∆EC^ mice, respectively.

**(D-E)** Western blot analysis of FMO2 expression in ECs sorting from FMO2^fl/fl^ and FMO2^△EC^ mice after AAV-NC and AAV-FMO2 infection. Results of four groups were normalized to measurements of β-actin and then to the mean for FMO2^fl/fl^ +AAV-NC group (*n =* 4 per group).

**(F-G)** Western blot analysis of FMO2 expression in ECs and non-ECs sorting from AAV-NC and AAV-FMO2 injected muscle tissues (*n =* 3 per group).

**(H-I)** Western blot analysis of FMO2 expression in ECs from retinal tissues of AAV-NC and AAV-FMO2 groups (*n =* 3 per group).

**(J-K)** Western blot analysis of FMO2 expression in ECs from NC and LV-FMO2 groups (*n =* 4 per group).

**(L-M)** Western blot analysis of FMO2 expression in ECs from Scrambled Control and LV-shFMO2 groups (*n =* 5 per group).

**(N-O)** Representative images of tube formation and quantitative statistics of the total tube length of ECs infected by Scrambled Control (*n =* 10), LV-shFMO2 (*n =* 9), NC (*n =* 6), LV-FMO2 (*n =* 6). Scale bar, 200 μm.

Quantified data are presented as mean ± SEM. Unpaired two-tailed Student’s *t*-test was conducted in **B**, **I**, **K**, **M**, and **O**. Two-way ANOVA followed by Tukey’s post hoc multiple comparisons was conducted in **E** and **G**. ns *P >* 0.05, * *P <* 0.05, ** *P <* 0.01, and *** *P <* 0.001.


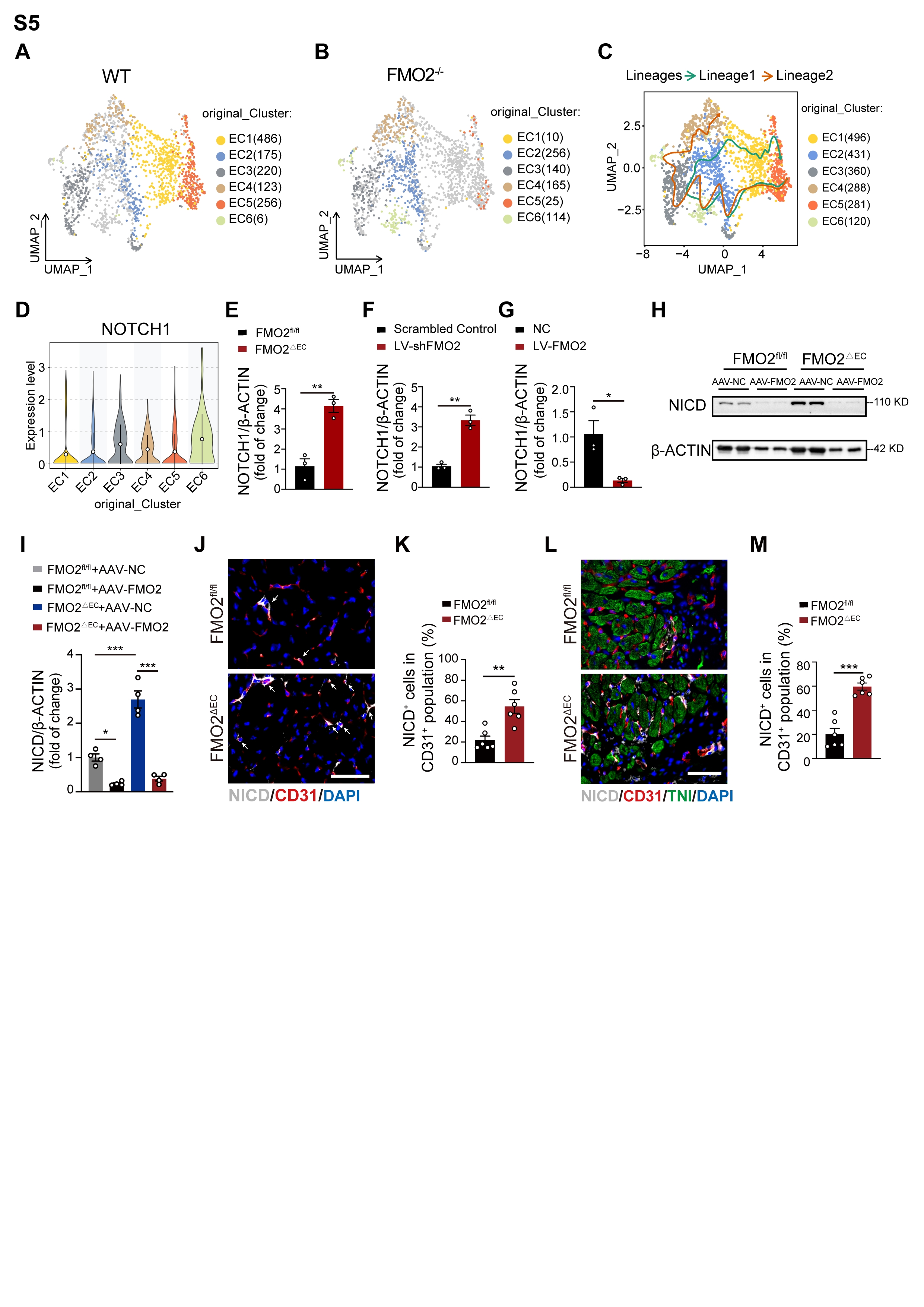


**Figure S5. FMO2 promotes angiogenesis by inhibiting NOTCH1**

**(A-B)** The UMAP of 6 clusters in WT group (A) and FMO2^-/-^ group (B).

**(C)** Pseudotime analysis of 6 clusters.

**(D)** The expression levels of *NOTCH1* in all clusters.

**(E)** mRNA level of *NOTCH1* in ECs sorting from FMO2^fl/fl^ and FMO2^△EC^ gastrocnemius muscle after HLI (*n =* 3 per group).

**(F)** mRNA level of *NOTCH1* in ECs infected with Scrambled Control or LV-shFMO2 (*n =* 3 per group).

**(G)** mRNA level of *NOTCH1* in ECs infected with NC or LV-FMO2 (*n =* 3 per group).

**(H-I)** Western blot analysis of NICD expression after AAV-NC or AAV-FMO2 injection in FMO2^fl/fl^ and FMO2^△EC^ mice (*n =* 4 per group).

**(J-K)** NICD and CD31 staining on gastrocnemius sections from FMO2^fl/fl^ and FMO2^△EC^ mice post-HLI, and nuclei were counterstained with DAPI. The proportion of CD31^+^ ECs with high NICD expression was quantified in **(K)** (*n =* 6 per group). Scale bar, 50 μm.

**(L-M)** NICD and CD31 staining on the border zone of MI hearts from FMO2^fl/fl^ and FMO2^△EC^ mice. Cardiomyocytes were visualized by staining for cardiac troponin I (TNI), and nuclei were counterstained with DAPI. The proportion of CD31^+^ ECs with high NICD expression was quantified in **(M)** (*n =* 6 per group). Scale bar, 50 μm.

Quantified data are presented as mean ± SEM. Unpaired two-tailed Student’s *t*-test was conducted in **E**, **F**, **G**, **K** and **M**. Two-way ANOVA followed by Tukey’s post hoc multiple comparisons was conducted in **I**. ns *P >* 0.05, * *P <* 0.05, ** *P <* 0.01, and *** *P <* 0.001.


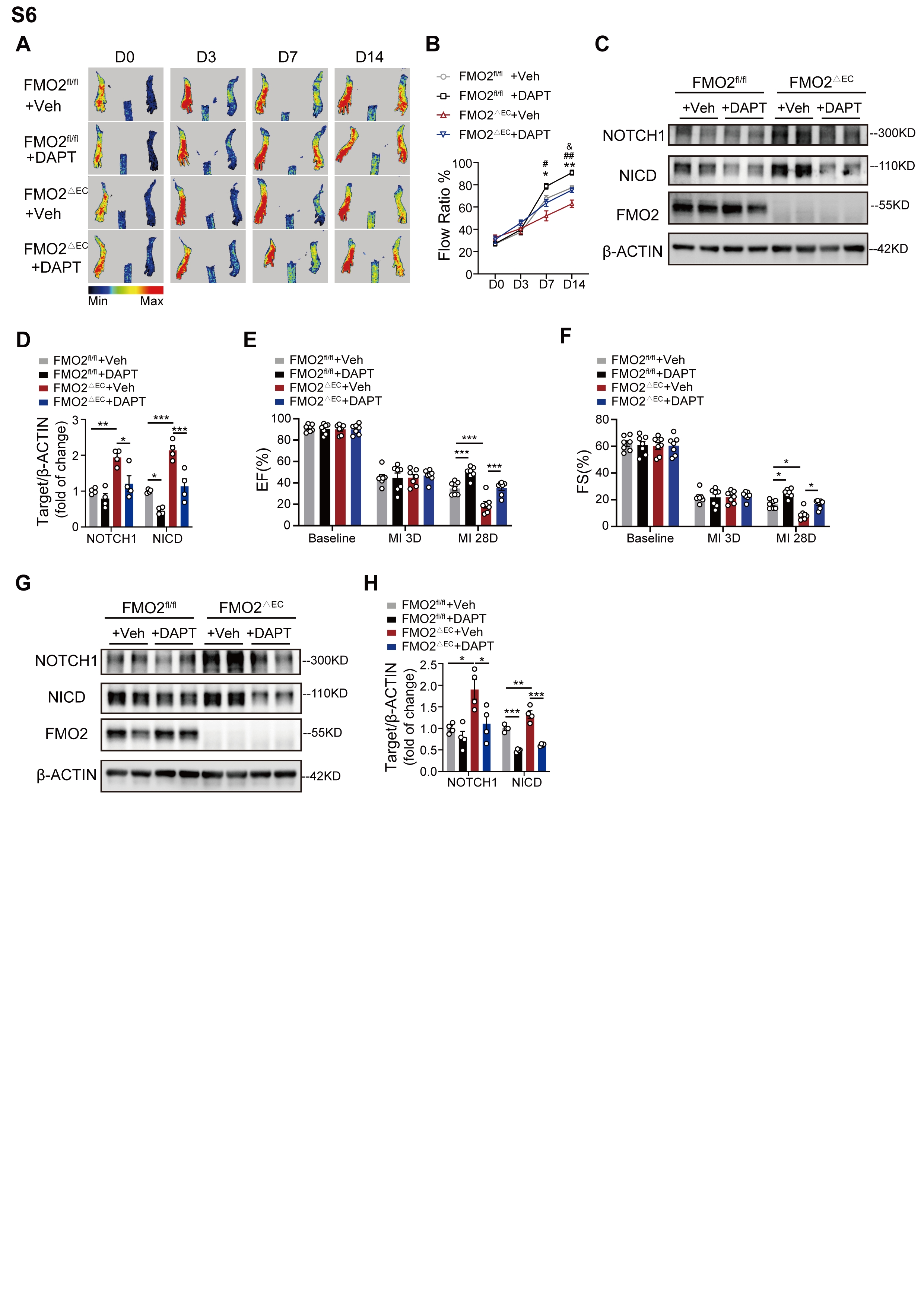


**Figure S6.** **DAPT** **restores angiogenesis in FMO2^△EC^ ischemic mice**

**(A-B)** Representative images showing Doppler flow images after injection of DAPT and vehicle in the FMO2^fl/fl^ and FMO2^∆EC^ groups, respectively. Quantitative summary of flow ratio in each group was shown in **(B)** (FMO2^fl/fl^+Veh group, *n =* 8; FMO2^fl/fl^+DAPT group, *n =* 8; FMO2^∆EC^+Veh group, *n =* 10; FMO2^∆EC^+DAPT group, *n =* 8) (* *P <* 0.05, ** *P <* 0.01, FMO2^fl/fl^+DAPT vs FMO2^fl/fl^+Veh; # *P <* 0.05, ## *P <* 0.01, FMO2^△EC^+Veh vs FMO2^fl/fl^+Veh; & *P <* 0.05, FMO2^△EC^+DAPT vs FMO2^△EC^+Veh).

**(C-D)** Western blot analysis of NOTCH1 and NICD levels in gastrocnemius ECs following DAPT or vehicle treatment in FMO2^fl/fl^ and FMO2^∆EC^ mice after HLI (*n =* 4 per group).

**(E-F)** Echocardiographic assessments of EF and FS were conducted at baseline, 3 days and 28 days after MI and DAPT or vehicle treatment in the FMO2^fl/fl^ and FMO2^∆EC^ groups, respectively (*n =* 7 per group).

**(G-H)** Western blot analysis of NOTCH1 and NICD levels in cardiac ECs following DAPT or vehicle treatment in FMO2^fl/fl^ and FMO2^∆EC^ mice after MI (*n =* 4 per group).

Quantified data are presented as mean ± SEM. Two-way ANOVA followed by Tukey’s post hoc multiple comparisons was conducted in **B**, **D**, **E**, **F** an **H**. ns *P >* 0.05, * *P <* 0.05, ** *P <* 0.01, and *** *P <* 0.001.


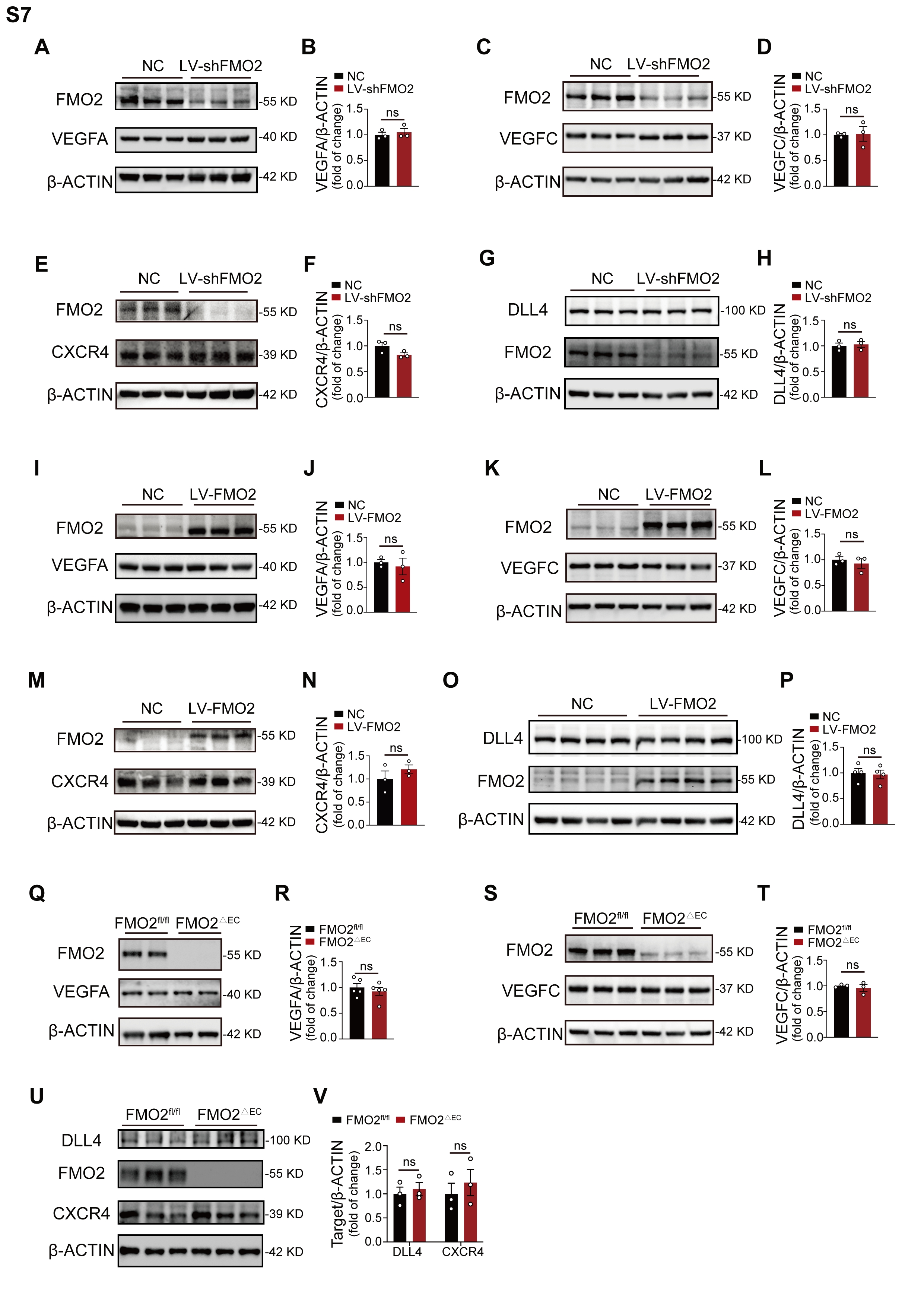


**Figure S7****. Expression of VEGFA, VEGFC, CXCR4 and DLL4 following FMO2 modulation both *in vivo* and *in vitro*.**

**(A-B)** Western blot analysis of VEGFA levels in ECs infected with NC or LV-shFMO2 (*n =* 3 per group).

**(C-D)** Western blot analysis of VEGFC levels in ECs infected with NC or LV-shFMO2 (*n =* 3 per group).

**(E-F)** Western blot analysis of CXCR4 levels in ECs infected with NC or LV-shFMO2 (*n =* 3 per group).

**(G-H)** Western blot analysis of DLL4 levels in ECs infected with NC or LV-shFMO2 (*n =* 3 per group).

**(I-J)** Western blot analysis of VEGFA levels in ECs infected with NC or LV-FMO2 (*n =* 3 per group).

**(K-L)** Western blot analysis of VEGFC levels in ECs infected with NC or LV-FMO2 (*n =* 3 per group).

**(M-N)** Western blot analysis of CXCR4 levels in ECs infected with NC or LV-FMO2 (*n =* 3 per group).

**(O-P)** Western blot analysis of DLL4 levels in ECs infected with NC or LV-FMO2 (*n =* 4 per group).

**(Q-R)** Western blot analysis of VEGFA levels in ECs from FMO2^fl/fl^ and FMO2^△EC^ mice (*n =* 5 per group).

**(S-T)** Western blot analysis of VEGFC levels in ECs from FMO2^fl/fl^ and FMO2^△EC^ mice (*n =* 3 per group).

**(U-V)** Western blot analysis of DLL4 and CXCR4 levels in ECs from FMO2^fl/fl^ and FMO2^△EC^ mice (*n =* 3 per group).

Quantified data are presented as mean ± SEM. Unpaired two-tailed Student’s *t*-test was conducted in **B**, **D**, **F**, **H**, **J**, **L**, **N**, **P**, **R**, **T** and **V**. ns *P >* 0.05, * *P <* 0.05, ** *P <* 0.01, and *** *P <* 0.001.


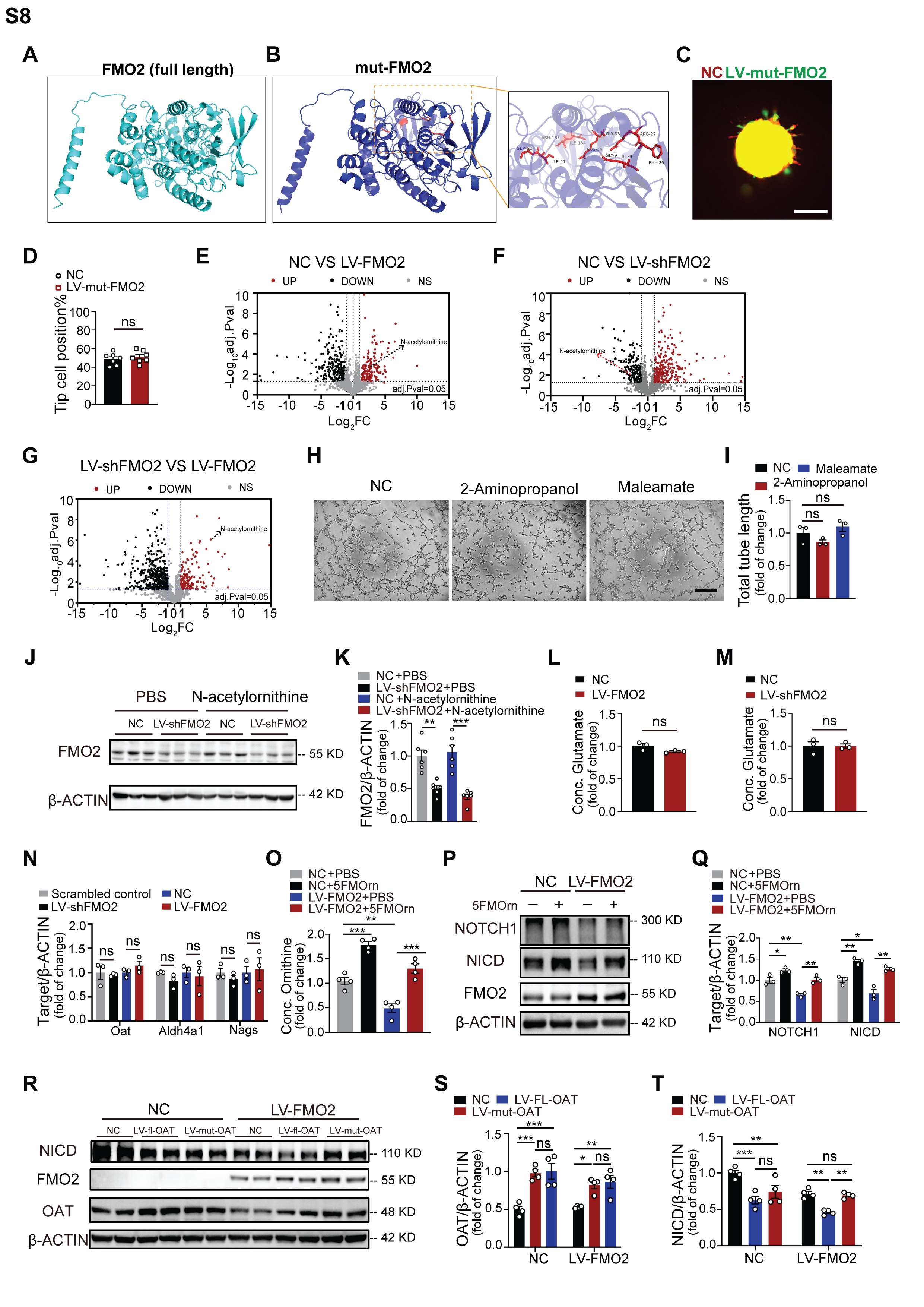


**Figure S8. FMO2 promotes angiogenesis via N-acetylornithine regulation**

**(A)** Overall structure of FMO2 (full length).

**(B)** Structural modeling of LV-mut-FMO2. The right panel shows the sites of enzyme activity mutations, with the residues immediately before and after the mutation sites represented as red sticks.

**(C-D)** Representative images and quantitative analysis of EC spheroids containing a 1:1 mixture of NC^RED^ and LV-mut-FMO2^GFP^ ECs showing a comparable fraction at the tip position (*n =* 7 per group).

**(E)** The volcano diagram showed differential metabolites detected by non-targeted metabolomics between NC and LV-FMO2 groups.

**(F)** The volcano diagram showed differential metabolites detected by non-targeted metabolomics between NC and LV-shFMO2 groups.

**(G)** The volcano diagram showed differential metabolites detected by non-targeted metabolomics between LV-FMO2 and LV-shFMO2 groups.

**(H-I)** Representative images of tube formation and quantitative statistics of the total tube length of ECs formed after treatment with different metabolites (2-Aminopropanol and Maleamate) and control (*n =* 3 per group). Scale bar, 200 μm.

**(J-K)** Western blot analysis of FMO2 expression in ECs following PBS or N-acetylornithine treatment after being infected with Scrambled Control or LV-shFMO2 (*n =* 6 per group).

**(L)** Quantitative analysis of glutamate levels in the LV-FMO2 and NC groups (*n =* 3 per group).

**(M)** Quantitative analysis of glutamate levels in the LV-shFMO2 and NC groups (*n =* 3 per group).

**(N)** The mRNA expression levels of *Oat*, *Aldh4a1* and *Nags* in the LV-FMO2 or LV-shFMO2 group (*n =* 3 per group).

**(O)** Quantitative analysis of Ornithine levels by ELISA in the NC+PBS, NC+5FMOrn, LV-FMO2+PBS, and LV-FMO2+5FMOrn groups (*n =* 4 per group).

**(P-Q)** Western blot analysis of NICD and NOTCH1 levels in ECs following PBS or 5FMOrn treatment after being infected with Scrambled Control or LV-FMO2 (*n =* 3 per group).

**(R-T)** Western blot analysis of OAT and NICD levels in ECs transfected with NC or LV-FMO2 and further infected with NC, LV-fl-OAT, or LV-mut-OAT (*n =* 4 per group).

Quantified data are presented as mean ± SEM. Unpaired two-tailed Student’s *t*-test was conducted in **D**, **L**, **M** and **N**. One-way ANOVA followed by Tukey’s post hoc multiple comparisons was conducted in **I**. Two-way ANOVA followed by Tukey’s post hoc multiple comparisons was conducted in **K**, **O**, **Q**, **S** and **T**. ns *P >* 0.05, * *P <* 0.05, ** *P <* 0.01, and *** *P <* 0.001.

**
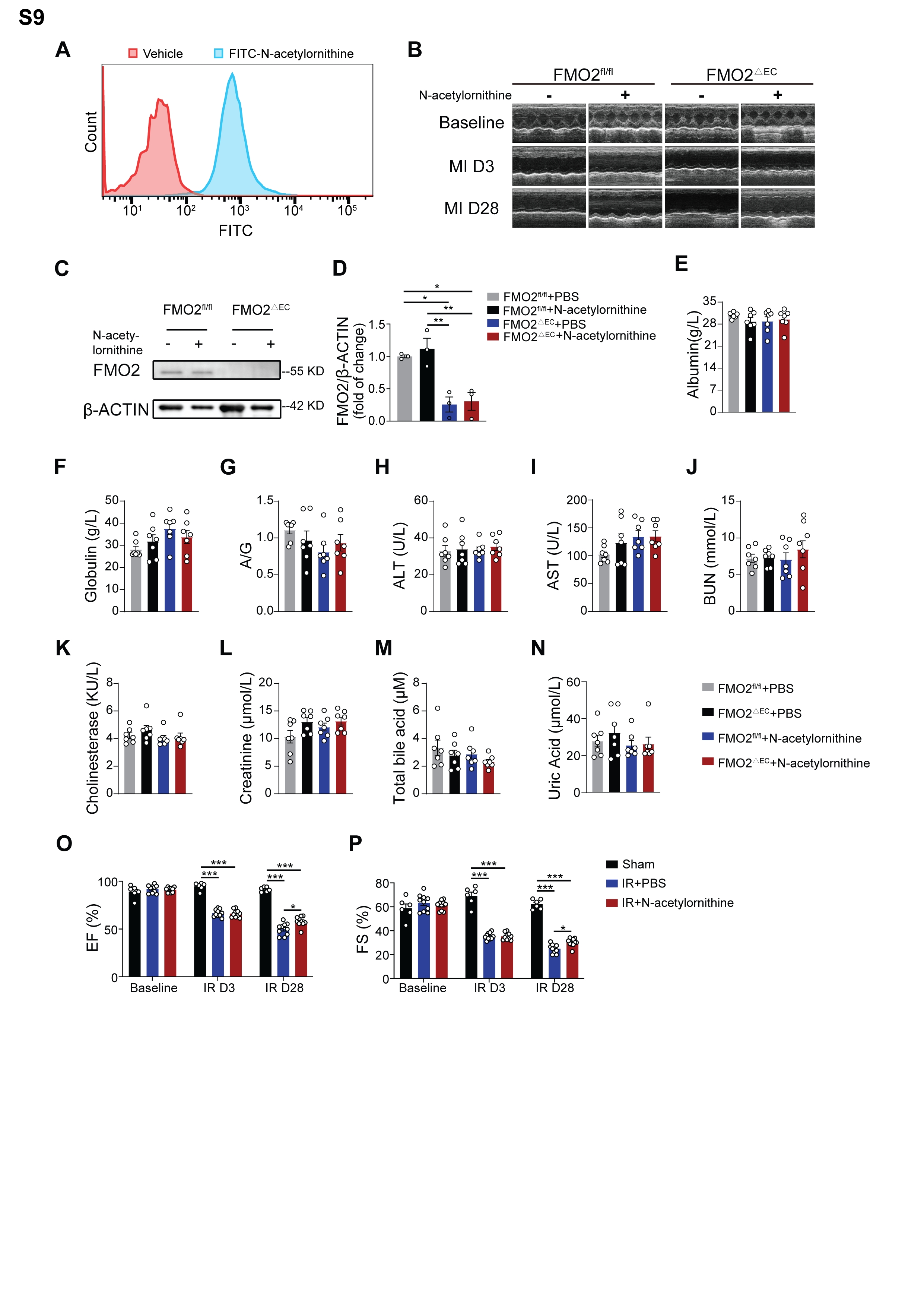
**

**Figure S9.** **N-acetylornithine promotes angiogenesis in ischemic diseases**

**(A)** Flow cytometry analysis of fluorescence intensity in ECs from the vehicle control and FITC-N- acetylornithine treatment groups.

**(B)** Representative cardiac ultrasound M-mode images of FMO2^△EC^ and FMO2^fl/fl^ mice following PBS or N-acetylornithine treatment at baseline, 3 and 28 days after MI.

**(C-D)** Western blot analysis of FMO2 expression in gastrocnemius ECs following PBS or N-acetylornithine treatment from FMO2^fl/fl^ and FMO2^∆EC^ mice after HLI (*n =* 3 per group).

**(E-N)** Liver and kidney functions of mice after exogenous injection of N-acetylornithine, represented by albumin, globulin, A/G, ALT, AST, BUN, Cholinesterase, Creatinine, Total bile acid and Unic Acid (*n =* 7 per group).

**(O-P)** Echocardiographic assessments of EF and FS were conducted at baseline, 3 and 28 days after IR surgery following PBS or N-acetylornithine treatment or Sham-operated mice. Sham group, *n =* 6; IR+PBS group, *n =* 10; IR+ N-acetylornithine, n=10.

Quantified data are presented as mean ± SEM. Two-way ANOVA followed by Tukey’s post hoc multiple comparisons was conducted in **D**, **E**, **F**, **G**, **H**, **I**, **J**, **K**, **L**, **M**, **N**, **O** and **P.** ns *P >* 0.05, * *P <* 0.05, ** *P <* 0.01, and *** *P <* 0.001.


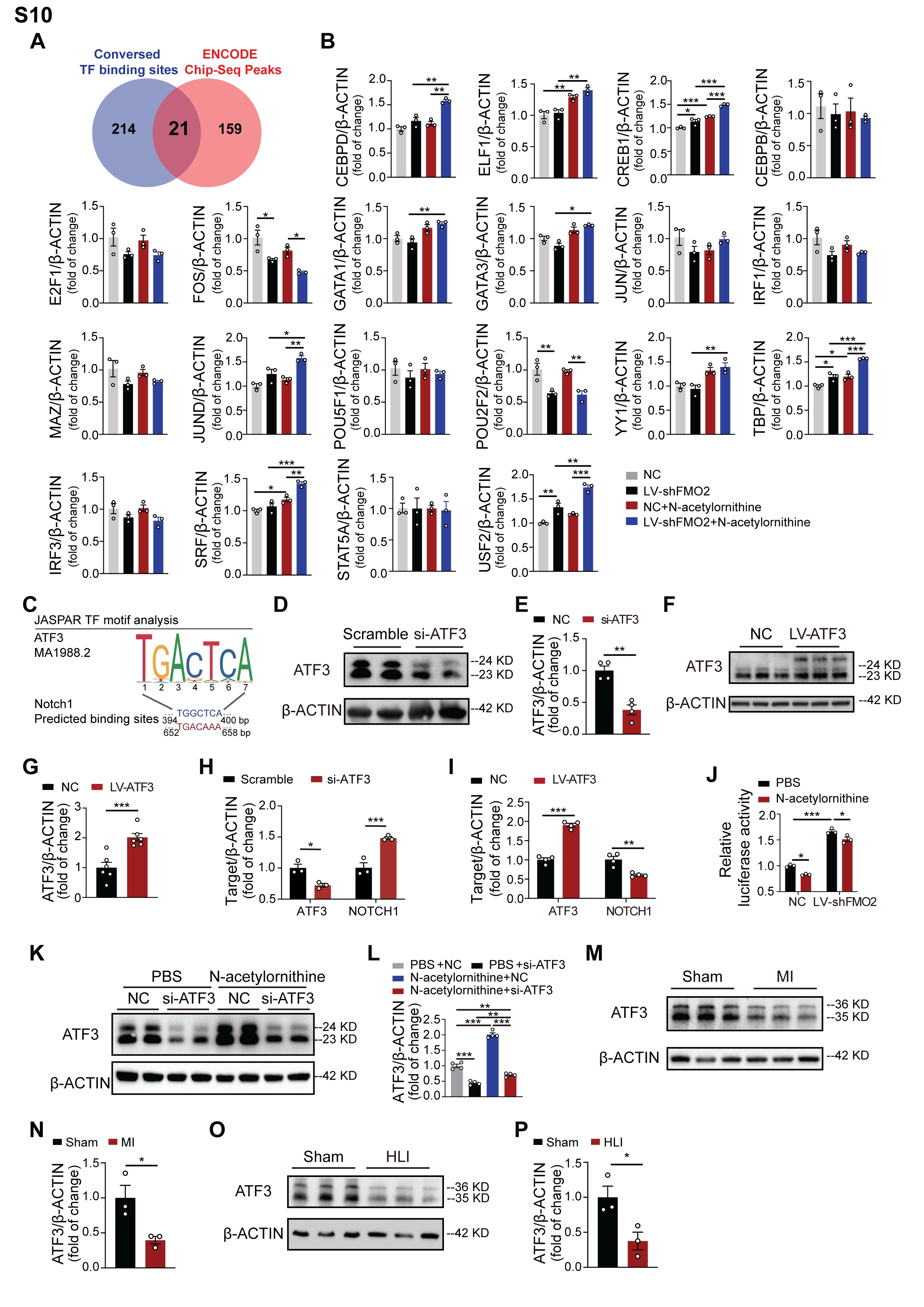


**Figure S10.** **N-acetylornithine inhibits NOTCH1 to promote angiogenesis by regulating ATF3**

**(A)** The Venn diagram represents the cross-over analysis of transcription factors predicted by conserved transcription factor binding sites and predicted by ENCODE chip-seq peaks.

**(B)** mRNA expression of the simulated transcription factors in ECs after adding N-acetylornithine treatment to LV-shFMO2 or NC groups, respectively (*n =* 3 per group).

**(C)** JASPAR motif analysis of ATF3 and predicted binding sites of *NOTCH1*.

**(D-E)** Western blot analysis of ATF3 expression in ECs after ATF3 silencing (*n =* 4 per group).

**(F-G)** Western blot analysis of ATF3 expression in ECs after ATF3 overexpressing (*n =* 6 per group).

**(H)** mRNA expression of *ATF3* and *NOTCH1* in ECs after ATF3 silencing (*n =* 3 per group).

**(I)** mRNA expression of *ATF3* and *NOTCH1* in ECs after ATF3 overexpressing (*n =* 4 per group).

**(J)** Comparison of the relative luciferase intensity of NOTCH1-promoter in ECs following PBS or N-acetylornithine treatment after FMO2 knockdown (*n =* 3 per group).

**(K-L)** Western blot analysis of ATF3 expression in ECs following ATF3 silencing and PBS or N-acetylornithine treatment (*n =* 4 per group).

**(M-N)** Western blot analysis of ATF3 expression in ECs isolated from sham-operated or MI hearts (*n =* 3 per group).

**(O-P)** Western blot analysis of ATF3 expression in ECs isolated from sham-operated or HLI gastrocnemius muscles (*n =* 3 per group).

Quantified data are presented as mean ± SEM. Unpaired two-tailed Student’s *t*-test was conducted in **D**, **F**, **G**, **I**, **N** and **P**. Two-way ANOVA followed by Tukey’s post hoc multiple comparisons was conducted in **B**, **J**, and **L**. ns *P >* 0.05, * *P <* 0.05, ** *P <* 0.01, and *** *P <* 0.001.


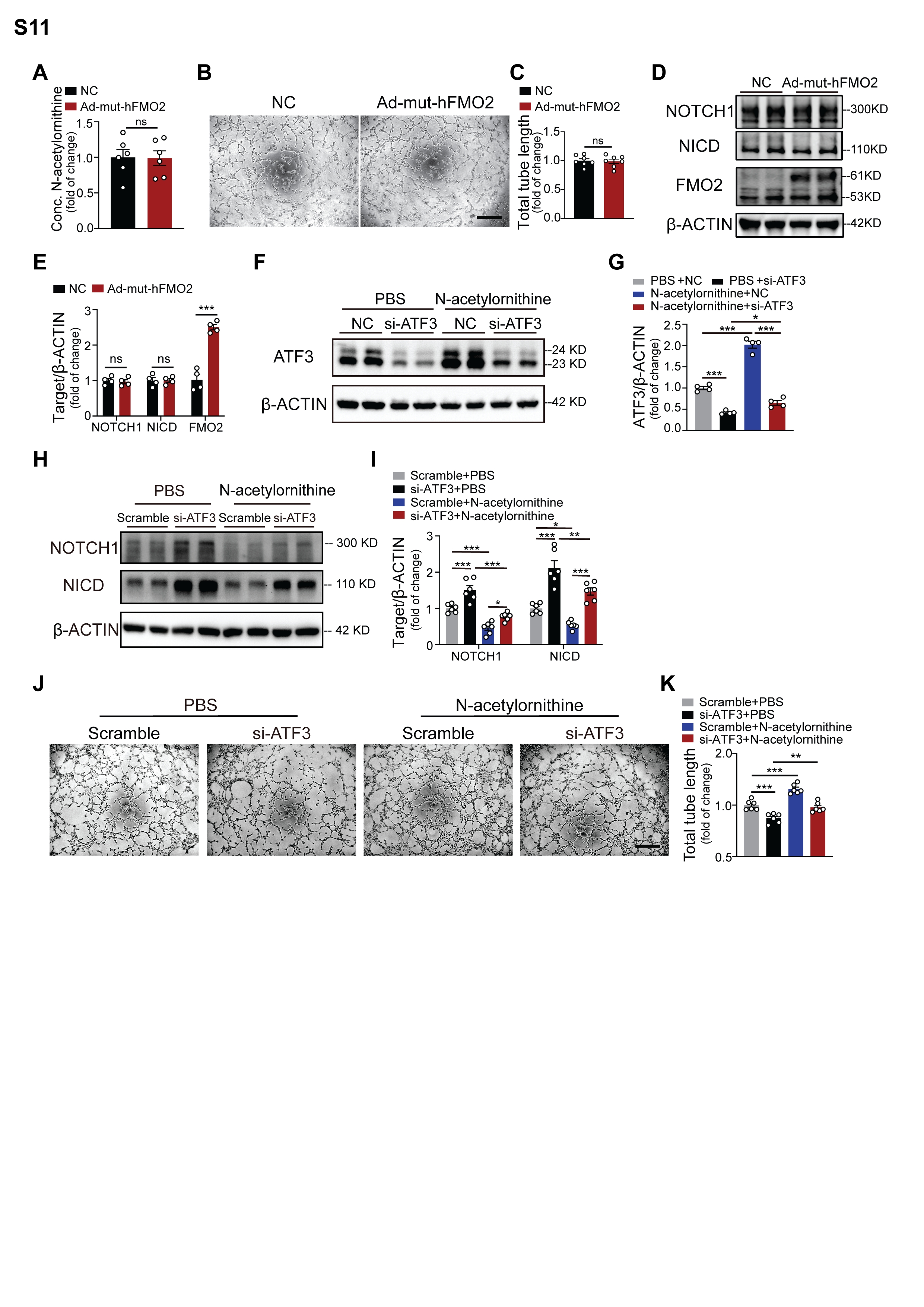


**Figure S11. Pro-angiogenic effect of FMO2 and N-acetylornithine in human ischemic diseases**

**(A)** Quantitative analysis of N-acetylornithine levels by UHPLC–MS/MS in primary HUVECs infected with NC or Ad-mut-hFMO2 (*n =* 6 per group).

**(B-C)** Representative images of tube formation and quantitative statistics of the total tube length of primary HUVECs infected with NC or Ad-mut-hFMO2 (*n =* 7 per group). Scale bar, 500 μm.

**(D-E)** Western blot analysis of FMO2, NICD and NOTCH1 expression in primary HUVECs infected with NC or Ad-mut-hFMO2 (*n =* 4 per group).

**(F-G)** Western blot analysis of ATF3 expression in primary HUVECs after ATF3 silencing and PBS or N-acetylornithine treatment (*n =* 4 per group).

**(H-I)** Western blot analysis of NICD and NOTCH1 expression in primary HUVECs after ATF3 silencing and PBS or N-acetylornithine treatment (*n =* 6 per group).

**(J-K)** Representative images of tube formation and quantitative statistics of the total tube length of primary HUVECs after ATF3 silencing and PBS or N-acetylornithine treatment (*n =* 6 per group). Scale bar, 200 μm.

Quantified data are presented as mean ± SEM. Unpaired two-tailed Student’s *t*-test was conducted in **A**, **C** and **E**. Two-way ANOVA followed by Tukey’s post hoc multiple comparisons was conducted in **G**, **I** and **K**. ns *P >* 0.05, * *P <* 0.05, ** *P <* 0.01, and *** *P <* 0.001.

**Table S1**

**Top 20** **downregulated genes in whole heart cells and ECs post MI**

|  | **All cells** | | | **ECs** | | |
| --- | --- | --- | --- | --- | --- | --- |
| **Rank** | **gene** | **avg_log_2_FC** | **p_val_adj** | **gene** | **avg_log_2_FC** | **p_val_adj** |
| 1 | Clec3b | -1.940146383 | <1E-300 | **Zbtb16** | **-1.957318479** | 1.07E-111 |
| 2 | Adamts5 | -1.929272263 | <1E-300 | Dapk1 | -1.704129627 | 5.58E-82 |
| 3 | Dpep1 | -1.856097373 | <1E-300 | P05Rik | -1.945932881 | 4.97E-72 |
| 4 | **Zbtb16** | **-1.786566042** | **<1E-300** | Klf9 | -1.224775699 | 1.57E-71 |
| 5 | Lpl | -1.771763121 | <1E-300 | Prkg1 | -2.028650456 | 7.87E-62 |
| 6 | Pcsk6 | -1.724889206 | <1E-300 | Tns1 | -1.273899875 | 1.40E-58 |
| 7 | Abca8a | -1.676659769 | <1E-300 | Sgms1 | -1.141752211 | 1.46E-56 |
| 8 | Smoc2 | -1.629600277 | <1E-300 | Ptpn14 | -1.089341881 | 1.27E-52 |
| 9 | Nid1 | -1.607228032 | <1E-300 | **Fmo2** | **-1.533937332** | 1.38E-48 |
| 10 | Lamb1 | -1.552466917 | <1E-300 | Tmtc1 | -1.412475206 | 1.64E-48 |
| 11 | Spon1 | -1.500009658 | <1E-300 | Wipf3 | -1.182949494 | 4.51E-45 |
| 12 | Aldh1a1 | -1.499676023 | <1E-300 | **Fkbp5** | **-1.207183393** | 3.27E-42 |
| 13 | Lama2 | -1.474806323 | <1E-300 | Apoe | -1.597903697 | 1.99E-41 |
| 14 | **Fmo2** | **-1.453585682** | **<1E-300** | Stxbp6 | -1.057127463 | 1.59E-40 |
| 15 | Tnxb | -1.423046302 | <1E-300 | Chrm3 | -1.274017106 | 6.24E-37 |
| 16 | Prex2 | -1.404928219 | <1E-300 | Cytl1 | -1.795641649 | 5.49E-34 |
| 17 | **Fkbp5** | **-1.400771092** | **<1E-300** | Nek7 | -1.040661078 | 1.95E-33 |
| 18 | Glul | -1.353945194 | <1E-300 | Cped1 | -1.111148439 | 9.35E-33 |
| 19 | Tsc22d3 | -1.344691809 | <1E-300 | Kcnt2 | -1.428221221 | 4.39E-31 |
| 20 | Sparcl1 | -1.341652461 | <1E-300 | Mir99ahg | -1.056375669 | 1.90E-30 |

**Table S2.**

**Flow ratio values in WT and FMO2^-/-^ mice subjected to HLI**

| Flow Ratio | WT (*n =* 9) | FMO2^-/-^ (*n =* 12) |
| --- | --- | --- |
| D0 | 30.48±2.2645 | 32.38±3.0551 |
| D3 | 48.92±3.9637 | 44.27±4.0760 |
| D7 | 51.98±5.1712 | 47.93±4.2728 |
| D14 | 72.12±4.5288 | 55.14±3.3338* |

N of each group was indicated. Quantified data are presented as mean ± SEM. * *P <* 0.05. FMO2^-/-^ vs WT.

**Table S3.**

**Echocardiography values in FMO2^△EC^ and FMO2^fl/fl^ mice subjected to MI**

| Parameters | FMO2^fl/fl^ (*n =* 10) | FMO2^△EC^ (*n =* 9) |
| --- | --- | --- |
| EF (%) Baseline | 80.01±1.1949 | 82.00±1.6295 |
| EF (%) MI 3D | 40.03±2.5964 | 40.68±2.0693 |
| EF (%) MI 28D | 31.48±2.0758 | 20.49±2.2896** |
| FS (%) Baseline | 47.12±1.2857 | 49.31±1.7761 |
| FS (%) MI 3D | 19.27±1.3895 | 19.70±1.1617 |
| FS (%) MI 28D | 14.79±1.0496 | 9.41±1.0786* |

N of each group was indicated. EF: Ejection fraction; FS: Fractional shortening. Quantified data are presented as mean ± SEM. * *P <* 0.05, ** *P <* 0.01. FMO2^△EC^ +MI vs FMO2^fl/fl^ +MI.

**Table S4.**

**Flow ratio values in FMO2^△EC^ and FMO2^fl/fl^ mice subjected to HLI**

| Flow Ratio | FMO2^fl/fl^ (*n =* 8) | FMO2^△EC^ (*n =* 10) |
| --- | --- | --- |
| D0 | 24.85±2.0423 | 24.55±2.8635 |
| D3 | 28.82±4.6356 | 28.07±3.3648 |
| D7 | 50.51±7.0547 | 42.00±4.4276 |
| D14 | 73.14±2.2387 | 47.46±5.3034*** |

N of each group was indicated. Quantified data are presented as mean ± SEM. *** *P <* 0.001. FMO2^△EC^ vs FMO2^fl/fl^.

**Table S5.**

**Flow ratio values in FMO2^△EC^ and FMO2^fl/fl^ mice following AAV-NC or AAV-FMO2 injection in HLI model**

| Flow Ratio | FMO2^fl/fl^+AAV-NC (*n =* 6) | FMO2^fl/fl^+AAV-FMO2 (*n =* 7) | FMO2^△EC^+AAV-NC (*n =* 9) | FMO2^△EC^+AAV-FMO2 (*n =* 6) |
| --- | --- | --- | --- | --- |
| D0 | 21.15±2.6592 | 25.20±2.5118 | 25.10±3.9415 | 23.42±2.4110 |
| D3 | 43.76±5.1358 | 45.47±5.1305 | 44.84±4.6344 | 43.88±6.1971 |
| D7 | 69.07±4.6776 | 73.02±5.5398 | 58.34±6.5114 | 72.69±6.5237 |
| D14 | 73.99±3.1299 | 93.49±1.4102* | 50.55±4.3969^##^ | 72.25±6.3810^&&^ |

N of each group was indicated. Quantified data are presented as mean ± SEM. * *P <* 0.05, FMO2^fl/fl^ +AAV-FMO2 vs FMO2^fl/fl^+AAV-NC; ## *P <* 0.01, FMO2^∆EC^+AAV-NC vs FMO2^fl/fl^+AAV-NC; && P<0.01, FMO2^∆EC^+AAV-FMO2 vs FMO2^∆EC^+AAV-NC.

**Table S6.**

**Flow ratio values in FMO2^△EC^ and FMO2^fl/fl^ mice following vehicle or DAPT treatment in HLI model**

| Flow Ratio | FMO2^fl/fl^+Veh  (*n =* 8) | FMO2^fl/fl^+DAPT (*n =* 8) | FMO2^△EC^+Veh  (*n =* 10) | FMO2^△EC^+DAPT (*n =* 8) |
| --- | --- | --- | --- | --- |
| D0 | 26.91±1.5251 | 27.11±1.9883 | 32.62±2.2655 | 30.79±3.1209 |
| D3 | 37.52±2.7146 | 39.66±4.0483 | 40.68±4.7076 | 45.88±2.5491 |
| D7 | 67.62±2.6566 | 78.63±2.4423* | 52.14±4.5045^#^ | 63.75±3.1821 |
| D14 | 77.39±1.6707 | 90.85±2.1889** | 62.91±3.3887^##^ | 75.20±2.1042^&^ |

N of each group was indicated. Quantified data are presented as mean ± SEM. * *P <* 0.05, ** *P <* 0.01, FMO2^fl/fl^+DAPT vs FMO2^fl/fl^+Veh; # *P <* 0.05, ## *P <* 0.01, FMO2^△EC^+Veh vs FMO2^fl/fl^+Veh; & *P <* 0.05, FMO2^△EC^+DAPT vs FMO2^△EC^+Veh.

**Table S7.**

**Echocardiography values in FMO2^△EC^ and FMO2^fl/fl^ mice following vehicle or DAPT treatment in MI model**

| Parameters | FMO2^fl/fl^ +Veh  (*n =* 7) | FMO2^fl/fl^ +DAPT  (*n =* 7) | FMO2^△EC^+Veh (*n =* 7) | FMO2^△EC^+DAPT (*n =* 7) |
| --- | --- | --- | --- | --- |
| EF (%) Baseline | 91.34±1.2609 | 90.38±1.7854 | 90.12±1.7412 | 90.15±1.7566 |
| EF (%)  MI 3D | 45.03±2.7894 | 44.69±4.5115 | 44.94±2.8991 | 46.85±2.0646 |
| EF (%)  MI 28D | 34.74±2.2565 | 49.62±2.0118*** | 19.53±3.0833*** | 35.36±2.4163^###^ |
| FS (%) Baseline | 61.97±2.0527 | 60.90±2.7059 | 60.13±2.5899 | 60.48±2.6928 |
| FS (%)  MI 3D | 22.01±1.6764 | 21.77±2.4804 | 21.91±1.7114 | 22.93±1.2051 |
| FS (%)  MI 28D | 16.59±1.1794 | 24.82±1.1860* | 8.99±1.5086* | 16.89±1.2365^#^ |

N of each group was indicated. EF: Ejection fraction; FS: Fractional shortening. Quantified data are presented as mean ± SEM. * *P <* 0.05, *** *P <* 0.001 vs FMO2^fl/fl^ +Veh; ^#^ *P <* 0.05, ^###^ *P <* 0.001 vs FMO2^△EC^+Veh.

**Table S8**

**List of compounds detected in LV-FMO2 or LV-shFMO2 groups through pairwise comparisons**

| **Rank** | **Compounds** | **NC vs LV-FMO2** | | **NC vs LV-shFMO2** | |
| --- | --- | --- | --- | --- | --- |
|  |  | Log_2_(Fold Change) | p_val_adj | Log_2_(Fold Change) | p_val_adj |
| 1 | N-acetylornithine | 2.8924 | 3.73E-06 | -2.7797 | 0.002368 |
| 2 | 2-Aminopropanol | 2.7439 | 2.17E-05 | -2.2385 | 7.13E-06 |
| 3 | Maleamate | 2.612 | 0.00024907 | -2.2948 | 0.002984 |
| 1 | D-Alanyl-D-Alanine | -1.6839 | 0.016209 | 1.8433 | 0.003139 |
| 2 | Ornithine | -1.3266 | 0.0017817 | 1.5004 | 0.045004 |
| 3 | L-Tyrosine | -1.2561 | 0.0023401 | 1.0982 | 0.048451 |

**Table S9.**

**Flow ratio values in FMO2^△EC^ and FMO2^fl/fl^ mice following PBS or N-acetylornithine treatment in HLI model**

| Flow Ratio | FMO2^fl/fl^+PBS  (*n =* 7) | FMO2^fl/fl^+N-acetylornithine (*n =* 9) | FMO2^△EC^+PBS  (*n =* 9) | FMO2^△EC^+ N-acetylornithine  (*n =* 9) |
| --- | --- | --- | --- | --- |
| D0 | 27.39±3.1953 | 24.48±1.0232 | 28.94±2.6689 | 27.42±3.4136 |
| D3 | 47.54±3.4779 | 52.38±4.1646 | 44.93±3.2763 | 49.61±6.5692 |
| D7 | 77.73±4.3049 | 80.90±2.4186 | 59.97±5.7038* | 72.16±4.2632 |
| D14 | 80.84±3.0338 | 95.90±1.1308^#^ | 61.39±4.7987** | 84.53±3.7620^&&&^ |

N of each group was indicated. Quantified data are presented as mean ± SEM. * *P* < 0.05, ** *P* < 0.01, FMO2^∆EC^+PBS vs FMO2^fl/fl^+PBS; # *P* < 0.05, FMO2^fl/fl^+N-acetylornithine vs FMO2^fl/fl^+PBS; &&& *P* < 0.001, FMO2^∆EC^+N-acetylornithine vs FMO2^∆EC^+PBS.

**Table S10**

**Echocardiography values in FMO2^△EC^ and FMO2^fl/fl^ following PBS or** **N-acetylornithine treatment in MI model**

| Parameters | FMO2^fl/fl^ +PBS  (*n =* 7) | FMO2^fl/fl^ +  N-acetylornithine  (*n =* 10) | FMO2^△EC^+PBS (*n =* 8) | FMO2^△EC^+  N-acetylornithine  (*n =* 9) |
| --- | --- | --- | --- | --- |
| EF (%) Baseline | 77.31±1.4300 | 80.46±1.1530 | 82.71±1.1968 | 81.52±1.2326 |
| EF (%)  MI 3D | 46.66±2.1288 | 45.04±1.9366 | 47.60±1.7223 | 49.17±2.6430 |
| EF (%)  MI 28D | 41.00±2.7184 | 44.11±3.4839 | 26.11±3.9305** | 38.12±4.4118^#^^#^ |
| FS (%) Baseline | 44.87±1.3693 | 48.19±1.2449 | 50.48±1.3329 | 48.73±1.2958 |
| FS (%)  MI 3D | 22.94±1.2522 | 21.91±1.0359 | 23.19±0.9364 | 24.12±1.4650 |
| FS (%)  MI 28D | 20.06±1.4648 | 22.07±1.9692 | 12.27±1.9883** | 18.70±2.4665^#^ |

N of each group was indicated. EF: Ejection fraction; FS: Fractional shortening. Quantified data are presented as mean ± SEM. * *P <* 0.05, ** *P <* 0.01 vs FMO2^fl/fl^ +PBS; ^#^ *P <* 0.05, ^##^ *P <* 0.01 vs FMO2^△EC^+PBS.

**Table S11**

**Baseline characteristics of patients with PAD subjected to amputation**

| **Patient** | **Sex** | **Age(years)** | **BMI** | **SBP/DBP** | **HR** | **Diagnosis** | **Comorbidities** |
| --- | --- | --- | --- | --- | --- | --- | --- |
| **1** | M | 73 | 22.86 | 139/80 | 79 | PAD | Hypertension; Diabetes; Prior stroke; Atrial fibrillation; |
| **2** | F | 63 | 20 | 116/60 | 97 | PAD | Hypertension; SLE; CAD |
| **3** | M | 79 | 27.55 | 121/72 | 78 | PAD | Hypertension |
| **4** | M | 86 | 19 | 124/76 | 99 | PAD | Hypertension; Diabetes; Atrial fibrillation |

PAD: Peripheral Arterial Disease

SLE: Systemic lupus erythematosus

CAD: Coronary Atherosclerotic Heart Disease

HR: Heart Rate

Sex (M: male; F: female)

**Table S12**

**Baseline characteristics of patients with PAD and healthy controls**

| **Patient** | **Sex** | **Age(years)** | **BMI** | **SBP/DBP** | **HR** | **PAD** | **Smoke** | **Drink** |
| --- | --- | --- | --- | --- | --- | --- | --- | --- |
| **PAD1** | M | 72 | 22.89 | 124/71 | 72 | Y | Y | N |
| **PAD2** | M | 76 | 25.51 | 145/68 | 108 | Y | N | N |
| **PAD3** | M | 74 | 23.77 | 151/79 | 64 | Y | N | N |
| **PAD4** | M | 80 | 22.74 | 141/74 | 88 | Y | Y | N |
| **PAD5** | F | 72 | 15.63 | 125/63 | 65 | Y | N | N |
| **PAD6** | M | 75 | 23.32 | 163/98 | 81 | Y | Y | Y |
| **PAD7** | M | 49 | 24.57 | 112/70 | 80 | Y | Y | N |
| **PAD8** | F | 77 | 20.13 | 124/62 | 61 | Y | N | N |
| **PAD9** | M | 63 | 24.05 | 136/74 | 85 | Y | N | N |
| **PAD10** | M | 72 | 20.08 | 114/55 | 78 | Y | N | N |
| **C1** | M | 66 | 20.24 | 156/78 | 69 | N | Y | Y |
| **C2** | F | 69 | 27.59 | 117/55 | 69 | N | N | N |
| **C3** | F | 60 | 16.73 | 113/58 | 61 | N | N | N |
| **C4** | F | 64 | 19.96 | 152/75 | 93 | N | N | N |
| **C5** | F | 51 | 26.45 | 133/83 | 70 | N | N | N |
| **C6** | F | 66 | 24.44 | 146/62 | 90 | N | N | N |
| **C7** | F | 54 | 45.01 | 133/92 | 70 | N | N | N |
| C8 | M | 76 | 25.02 | 138/69 | 60 | N | N | N |

PAD: Peripheral Arterial Disease; C: Control patients; HR: Heart Rate

Sex (M: male; F: female); PAD (Y: yes; N: no);

Smoke (Y: yes; N: no); Drink(Y: yes; N: no)

**Table S13**

**Baseline characteristics of patients with STEMI and healthy controls**

| **Group** | **Sex** | | **Age**  **(years)** | **BMI** | **HBP** | **smoker** | **Culprit vessels** |
| --- | --- | --- | --- | --- | --- | --- | --- |
|  | **Male** | **Female** |  |  |  |  |  |
| **Control** | 16 | 20 | 60.2±1.2 | 24.7±0.8 | 11 | 11 | — |
| **STEMI** | 18 | 21 | 59.6±1.0 | 24.0±0.6 | 18 | 14 | LAD: 9  LCX: 1  RCA: 5  LAD+LCX: 10  LAD+RCA: 5  LCX+RCA: 4  LCX+RCA+LAD: 5 |

Values of Age and BMI are the means±SD

STEMI: acute ST-segment-elevation myocardial infarction

HBP: high blood pressure

LAD: left anterior descending artery

LCX: left circumflex artery

RCA: right coronary artery

**Table S14 Primers**

| **Real time PCR primers Gene** | **Species** | **Primers** |
| --- | --- | --- |
| ***Fmo2*** | Mus musculus | Forward: CCCAACTTCCTGCACAACTC  Reverse: TCCTTTCCGTTGCTCTGAGT |
| ***Zbtb16*** | Mus musculus | Forward:  GGAGACCATCCAGGCATCTG  Reverse:  ATCGAGCCTTACGGTCCTCT |
| ***Fkbp15*** | Mus musculus | Forward:  GGAGGGATGGGTTGTGAAGG  Reverse:  ACCAGGGCTCTCAAACATGG |
| ***Notch1*** | Mus musculus | Forward:  TGCCTGAATGGAGGTAGGTGC  Reverse:  GCACAGCGATAGGAGCCGATC |
| ***Atf 3*** | Mus musculus | Forward:  TCTGCGCTGGAGTCAGTTAC  Reverse:  TTGTTTCGACACTTGGCAGC |
| ***Cebpb*** | Mus musculus | Forward: GAAGACGGTGGACAAGCTGA  Reverse:  GCTTGAACAAGTTCCGCAGG |
| ***Creb1*** | Mus musculus | Forward:  GAGCAGACAACCAGCAGAGT  Reverse:  TCTTCAATCCTTGGCACCCC |
| ***Cebpd*** | Mus musculus | Forward:  GACTCCTGCCATGTACGACG  Reverse:  GTTGAAGAGGTCGGCGAAGA |
| ***Elf1*** | Mus musculus | Forward:  TTTGCAAGTAACGGCATGGAG  Reverse:  GTACAGGAGGGCTGGGAGAAT |
| ***E2f1*** | Mus musculus | Forward:  CTAGGGAGTGCGCACAGTTG  Reverse:  AGTTCAGGTCAACGACACCG |
| ***Fos*** | Mus musculus | Forward:  TACTACCATTCCCCAGCCGA  Reverse:  GCTGTCACCGTGGGGATAAA |
| ***Gata1*** | Mus musculus | Forward:  GGAGCTGACTTTCCCAGTCC  Reverse:  CGCCAGAGTGTTGTAGTGGT |
| ***Gata3*** | Mus musculus | Forward:  GCTACGGTGCAGAGGTATCC  Reverse:  GCGGATAGGTGGTAATGGGG |
| ***Jun*** | Mus musculus | Forward:  TGGGCACATCACCACTACAC  Reverse:  TCTGGCTATGCAGTTCAGCC |
| ***Irf1*** | Mus musculus | Forward:  CCAGCCGAGACACTAAGAGC  Reverse:  GACTCCCATCAGGAGGTTTCC |
| ***Maz*** | Mus musculus | Forward:  TGAGGCAGCTTTTGCTACGA  Reverse:  CCTCACCAGTACCTTTGTTGC |
| ***Jund*** | Mus musculus | Forward:  TACGCAGTTCCTCTACCCGA  Reverse:  AAACTGCTCAGGTTGGCGTA |
| ***Pou5f1*** | Mus musculus | Forward:  CCCGGAAGAGAAAGCGAACT  Reverse:  CCAAGCTGATTGGCGATGTG |
| ***Pou5f2*** | Mus musculus | Forward:  GCCGAGAAGCAAAGTCTGGA  Reverse:  AGCCTTGATCTTGGTGCTGG |
| ***Yy1*** | Mus musculus | Forward:  GCCCTCATAAAGGCTGCACA  Reverse:  TGAGCTCTCAACGAACGCTT |
| ***Tbp*** | Mus musculus | Forward:  CCTATCACTCCTGCCACACC  Reverse:  TGCACGAAGTGCAATGGTCT |
| ***Srf*** | Mus musculus | Forward:  TACCCTAGTCCCCATGCAGT  Reverse:  GAACACCTGAGGGACACCAC |
| ***Stat5a*** | Mus musculus | Forward:  CACTCCTGTACTTGGTTCGTCA  Reverse:  TCAGGGTTGGGTGGGTACAT |
| ***Usf2*** | Mus musculus | Forward:  GACATACCGCGTAGTCCAGG  Reverse:  TGAATTACAGCCTGGGTCACA |
| ***Irf3*** | Mus musculus | Forward:  AGCCCTGAACCGGAAAGAAG  Reverse:  CCCAGATGTACGAAGTCCCG |
| ***Oat*** | Mus musculus | Forward:  CCATTAAACCAGGCGAGCAC  Reverse:  GGATAGCGCCCATCTTGTCT |
| ***Aldh4a1*** | Mus musculus | Forward:  AGCTGGTTGACAGCACTACC  Reverse:  CGTAGCCTCCTGGACAATCG |
| ***Nags*** | Mus musculus | Forward:  GCTAAGGGTCGTGTCCAGTC  Reverse:  GGCTTGTCCACCGAATGGTA |
| ***β-actin*** | Mus musculus | Forward:  GTACTCTGTGTGGATCGGTGG  Reverse:  AGGGTGTAAAACGCAGCTCAG |

**Table S15 Primary antibodies**

| **Antibodies** | **SOURCE** | **DILUTION** |
| --- | --- | --- |
| α-SMA | ab5694 (abcam, US) | 1:200 (IF) |
| CD31 | ab222783 (abcam, US) | 1:200 (IF) |
| CD31 | ab64543 (abcam, US) | 1:1000 (WB) |
| Cardiac Troponin I | ab56357 (abcam, US) | 1:200 (IF) |
| FMO2 | NBP1-85952 (novus, US) | 1:500 (WB) |
| NICD | 4147 (CST, US) | 1:1000 (WB) |
| NOTCH1 | 4380 (CST, US) | 1:1000 (WB) |
| ATF3 | 18665 (CST, US) | 1:1000 (WB) |
| OAT | DF4270 (Affinity Biosciences, China) | 1:1000 (WB) |
| Ki67 | 9129 (CST, US) | 1:200 (IF) |
| PDGFRβ | 14-1402-82 (Invitrogen™, US) | 1:100 (IF) |
| active Notch | ab52301 (abcam, US) | 1:100 (IF) |
| DLL4 | ER1706-29 (HUABIO, China) | 1:1000 (WB) |
| VEGFA | ab214424 (abcam, US) | 1:2000 (WB) |
| VEGFC | Sc-374628 (santa cruz biotechnology) | 1:1000 (WB) |
| CXCR4 | ab124824 (abcam, US) | 1:500 (WB) |
| CD11b | ab133357 (abcam, US) | 1:1000 (WB) |
| Alexa Fluor 488  anti-NG2 | ab306569 (abcam, US) | 1:100 (Flow cytometry) |
| APC-Cy™7 Rat  Anti-Mouse CD45 | 557659 (BD biosciences) | 1:200 (Flow Cytometry) |
| BUV395 Rat  Anti-CD11b | 563553 (BD biosciences) | 1:200 (Flow Cytometry) |
| CD31 Monoclonal Antibody, PE | 12-0311-83 (Invitrogen™, US) | 1:200 (Flow Cytometry) |
| β-ACTIN | KC-5A08  (KANGCHEN, Shanghai, China) | 1:5000 |

**Table S16 Chemicals and Reagents**

| **Chemicals and Reagents** | **SOURCE** |
| --- | --- |
| CellTracker Red | Cat# C34552 (Invitrogen™) |
| DAPT | Cat# D5942 (Sigma-Aldrich) |
| isolectin GS-IB4,  Alexa Fluor® 594 conjugate | I21413 (Invitrogen™) |
| Microfil solution | Cat# MV-122 (Flow Tech) |
| Methocel | Cat# M0512 (Sigma-Aldrich) |
| VEGF Recombinant Mouse Protein | Cat# PMG0114 (Life Technologies) |
| Collagen, Type I, Rat tail | Cat# 354236 (Corning) |
| Matrigel Matrix (GFR) | Cat# 354230 (Corning) |
| Collagenase type IV | Cat# 17104019 (GIBCO) |
| CD31 MicroBeads, mouse | Cat# 130-097-418 (Miltenyi Biotec) |
| CD45 MicroBeads, mouse | Cat# 130-052-301 (Miltenyi Biotec) |
| Endothelial Cell Growth Medium 2 | Cat# C-22011 (Sigma-Aldrich) |
| Endothelial Cell Medium | Cat# 1001 (advll) |
| Tamoxifen | Cat# T5648 (Sigma-Aldrich) |
| N-acetylornithine | Cat# HY-113080 (MedChemExpress) |
| N-acetylornithine-d2 | Cat# HY-113080S (MedChemExpress) |
| TRIzol™ Reagent | Cat# 15596018 (Invitrogen™) |
| Lipofectamine™ RNAiMAX | Cat# 13778150 (Invitrogen™) |
| Lipofectamine™ 3000 | Cat# L3000008 (Invitrogen™) |
| Triton X-100 | Cat# 9036-19-5 (Sigma-Aldrich) |
| DAPI | Cat# H-1200 (Vectorlabs) |
| 2-Aminopropanol | Cat# 192171 (Sigma-Aldrich) |
| Maleamate | Cat# 445495 (Sigma-Aldrich) |
| Ornithine aminotransferase activity detection kit | OAT-W96-N(1620) (mlbio) |
| Glutamate Assay Kit | Cat# MAK004 (Sigma-Aldrich) |
| ELISA Kit for Ornithine | CB11156-Mu (COIBO BIO) |
| Anti-Flag Magnetic Beads | Cat# B26101 (selleck) |
| 5-FMOrn dihydrochloride | Cat# HY-154021A (MedChemExpress) |
| SimpleChIP® Enzymatic Chromatin IP Kit (Magnetic Beads) | Cat# 9003 (CST) |
| Renilla-Firefly Luciferase Dual Assay Kit | HY-K1013 (MedChemExpress) |

**Table S17 Oligonucleotides**

| **Oligonucleotides** | **Sequence** |
| --- | --- |
| siRNA targeting sequence: Scrambled | Sense:  UUCUCCGAACGUGUCACGUTT  Antisense:  ACGUGACACGUUCGGAGAATT |
| siRNA targeting sequence: mouse-Atf3 | Sense:  CACCCUUUGUCAAGGAAGATT  Antisense:  UCUUCCUUGACAAAGGGUGTT |
| siRNA targeting sequence: human-ATF3 | Sense:  GGUUUGCCAUCCAGAACAATT  Antisense:  UUGUUCUGGAUGGCAAACCTT |
